# Chemically programmable bacterial probes for the recognition of cell surface proteins

**DOI:** 10.1016/j.mtbio.2023.100669

**Published:** 2023-05-23

**Authors:** Pragati K. Prasad, Noa Eizenshtadt, Inna Goliand, Liat Fellus-Alyagor, Roni Oren, Ofra Golani, Leila Motiei, David Margulies

**Affiliations:** a Department of Chemical and Structural Biology, Weizmann Institute of Science Rehovot, 7610001, Israel; b Life Sciences Core Facilities, Weizmann Institute of Science, Rehovot, 7610001, Israel; c Department of Veterinary Resources, Weizmann Institute of Science, Rehovot, 7610001, Israel

**Keywords:** Self-assembly, DNA nanotechnology, Cell surface engineering, Supramolecular chemistry, Fluorescent probes

## Abstract

Common methods to label cell surface proteins (CSPs) involve the use of fluorescently modified antibodies (Abs) or small-molecule-based ligands. However, optimizing the labeling efficiency of such systems, for example, by modifying them with additional fluorophores or recognition elements, is challenging. Herein we show that effective labeling of CSPs overexpressed in cancer cells and tissues can be obtained with fluorescent probes based on chemically modified bacteria. The bacterial probes (B-probes) are generated by non-covalently linking a bacterial membrane protein to DNA duplexes appended with fluorophores and small-molecule binders of CSPs overexpressed in cancer cells. We show that B-probes are exceptionally simple to prepare and modify because they are generated from self-assembled and easily synthesized components, such as self-replicating bacterial scaffolds and DNA constructs that can be readily appended, at well-defined positions, with various types of dyes and CSP binders. This structural programmability enabled us to create B-probes that can label different types of cancer cells with distinct colors, as well as generate very bright B-probes in which the multiple dyes are spatially separated along the DNA scaffold to avoid self-quenching. This enhancement in the emission signal enabled us to label the cancer cells with greater sensitivity and follow the internalization of the B-probes into these cells. The potential to apply the design principles underlying B-probes in therapy or inhibitor screening is also discussed here.

## Graphical abstract

Decorating bacteria with modified DNA duplexes is presented as a general strategy to obtain fluorescent bacterial probes (B-probes) that can selectively identify cancer cells. The high structural programmability of such systems enabled the creation of B-probes that can selectively label cancer cells and tissues with different emission colors and with enhanced brightness.

**Figure:**
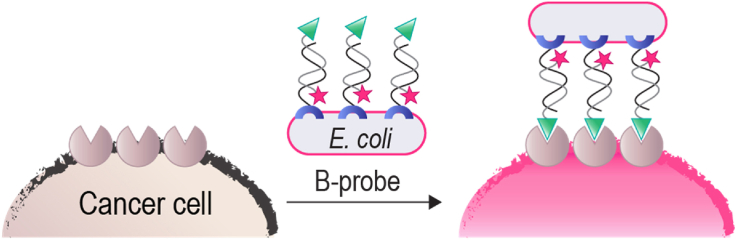

## 1 Introduction

**Fig. 1:**
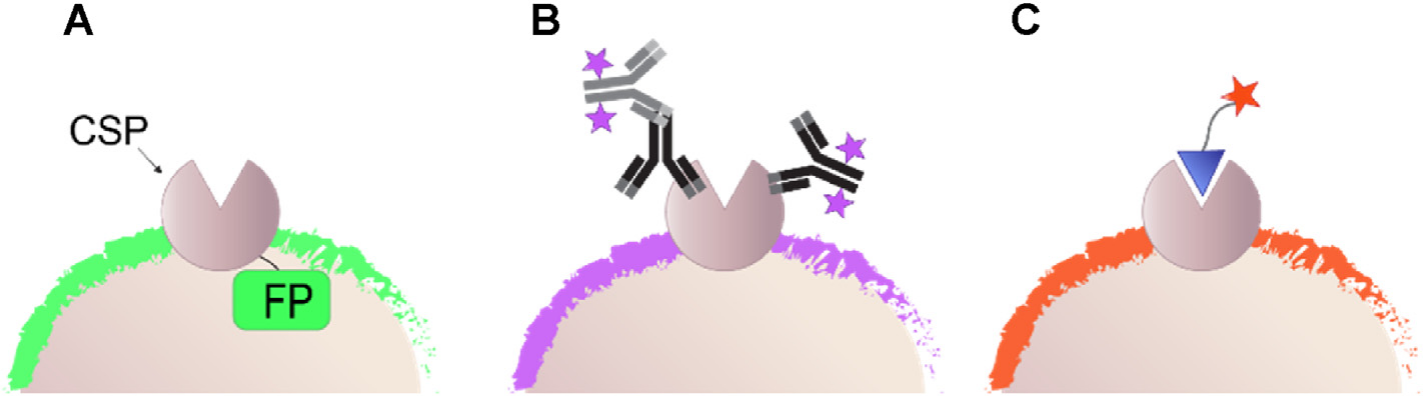
Schematic representation of fluorescent CSP labeling using (A) FP fusion, (B) IF, or (C) synthetic probes that target small-molecule binding sites of CSPs.

Fluorescence labeling is one of the most powerful analytical tools used to study protein expression and localization in intact cells [1]. A common method to label the proteins of interest (POIs) in a cellular environment is by fusing them to fluorescent proteins (FPs) (Fig. 1A) [2]. However, because this approach or similar approaches based on synthetic tags [[3], [4], [5], [6], [7]] require genetic engineering, they are not suitable for various medical diagnostic applications, which involve the classification of cells (e.g., cancer cells) according to the expression of specific cell surface proteins (CSPs) [8]. To detect, image, and classify CSPs of native (non-engineered) cells, immunofluorescence (IF) is commonly used (Fig. 1B) [9]. IF, however, is generally costly and laborious because it involves the manufacturing of non-homogeneous populations of fluorescently labeled monoclonal antibodies (Abs), as well as target identification via sequential incubation steps that frequently involve the use of both primary and secondary Abs (Fig. 1B). Moreover, because fluorescent Abs do not generally interact with small-molecule binding sites, they cannot be used to track the interaction between CSPs and small-molecule agonists or antagonists. An alternative approach to fluorescently label CSPs of non-engineered cells is by using molecular probes consisting of a fluorophore conjugated to a small-molecule- or a peptide-based ligand (Fig. 1C) [10].

Although this approach enabled the creation of homogeneous populations of structurally identical probes, which can be used to label and detect a wide range of POIs [[11], [12], [13], [14], [15], [16], [17], [18]], image CSPs in living cell [[19], [20], [21], [22], [23], [24], [25], [26], [27], [28], [29]], or perform live cell-based screening of CSP inhibitors [24], this labeling method has some limitations when compared to IF. One limitation is the difficulty to maintain the high binding affinity of a small-molecule ligand toward the POI following its conjugation to a fluorescent dye. This decrease in affinity often requires using a large excess of probes, which can lead to the generation of a strong background signal. Because removal of an excess of unbound probes by washing could lead to the dissociation of the probe-POI complex, efficient CSP labeling frequently requires developing unique sets of fluorescent molecular probes, for example, probes that covalently bind to their targets [19,21,25] or that can generate a ‘turn-on’ emission signal upon binding [24,27,29]. Therefore, unlike fluorescent Abs, which can be used to label various targets with a wide range of fluorescent dyes [30,31], obtaining synthetic probes that can label CSPs in living cells with high binding affinity, target versatility, and color variability remains challenging. The difficulty to obtain efficient CSP binding probes complicates using them in multiplexed protein detection, as well as complicates enhancing the intensity of their fluorescence signal by replacing the fluorescent reporter or by integrating several fluorophores into a single synthetic probe.

One way to enhance the binding affinity of synthetic agents to their biological targets is to utilize the multivalency effect [[32], [33], [34]]. With this strategy, multiple synthetic ligands are attached to a single scaffold to afford multivalent CSP binders that exhibit binding cooperativity and consequently, an increased affinity. In this context, we have recently shown that engineered bacteria decorated with a folate-bearing DNA duplex can bind to KB cancer cells overexpressing the folate receptor [35]. One advantage of synthetic protein binders generated from modified oligodeoxynucleotides (ODNs) [35], over Abs [8] (Fig. 1B) or binders based on unimolecular synthetic ligands [10] (Fig. 1C), is the structural modularity of synthetic ODNs and their ability to self-assemble into well-defined constructs [13,[36], [37], [38], [39], [40], [41], [42], [43], [44], [45], [46], [47], [48], [49], [50]]. We therefore hypothesized that by systematically modifying the structures of the modified DNA duplexes, it might be possible to diversify, optimize, and generalize the properties of chemically modified bacterial probes. We anticipated that such steps may enable the development of a wide range of fluorescent bacterial probes, termed herein B-probes, which can complement the current CSP labeling tools that mainly rely on Abs or small-molecule-based ligands (Fig. 1).

Herein we describe several innovations that were introduced to the bacterial-based fluorescent labeling strategy, which enabled the creation of B-probes that can bind to different CSPs and that can emit at different colors with enhanced brightness. These features enabled the probes to identify three types of cancer cells, even when the B-probes were combined in a single solution. Our results indicate the programmability, selectivity, and target versatility of B-probes, as well as the potential to use them to obtain rapid, multiplexed detection. The applicability of this labeling technology and the way it differs from labeling CSPs with fluorescently labeled small-molecule ligands (Fig. 1C), was further demonstrated by using B-probes to fluorescently label *ex-vivo* tissue derived from a human tumor-bearing mouse model, as well as by developing brighter, 2nd generation B-probes bearing multiple fluorophores. We show that by controlling the orientation of the dyes and the distances between them, self-quenching of fluorescence can be prevented, enabling the 2nd generation B-probes to generate stronger emission signals and be less susceptible to photobleaching. These properties enabled them to label cancer cells with higher sensitivity and to follow the internalization of the chemically modified bacteria into cancer cells. The high selectivity of the unnatural bacterium-cancer cell interactions presented here and the fact that, unlike with IF labeling (Fig. 1B), these interactions engage the small-molecule binding site of CSPs also indicate the potential to apply the design principles underlying B-probes in live cell-based inhibitor screening or to label CSPs for which there are no antibodies available. It can also be expected that these design principles would contribute to the future development of bacterial therapeutics [51] and to the emerging research direction in which bacteria decorated with synthetic DNA constructs [52] are used to create living materials, probes, and functional devices [[52], [53], [54], [55], [56]].

## 2 Results and discussion

### 2.1 Design of the *1*st generation B-probes intended to selectively label distinct cancer cells

**Fig. 2:**
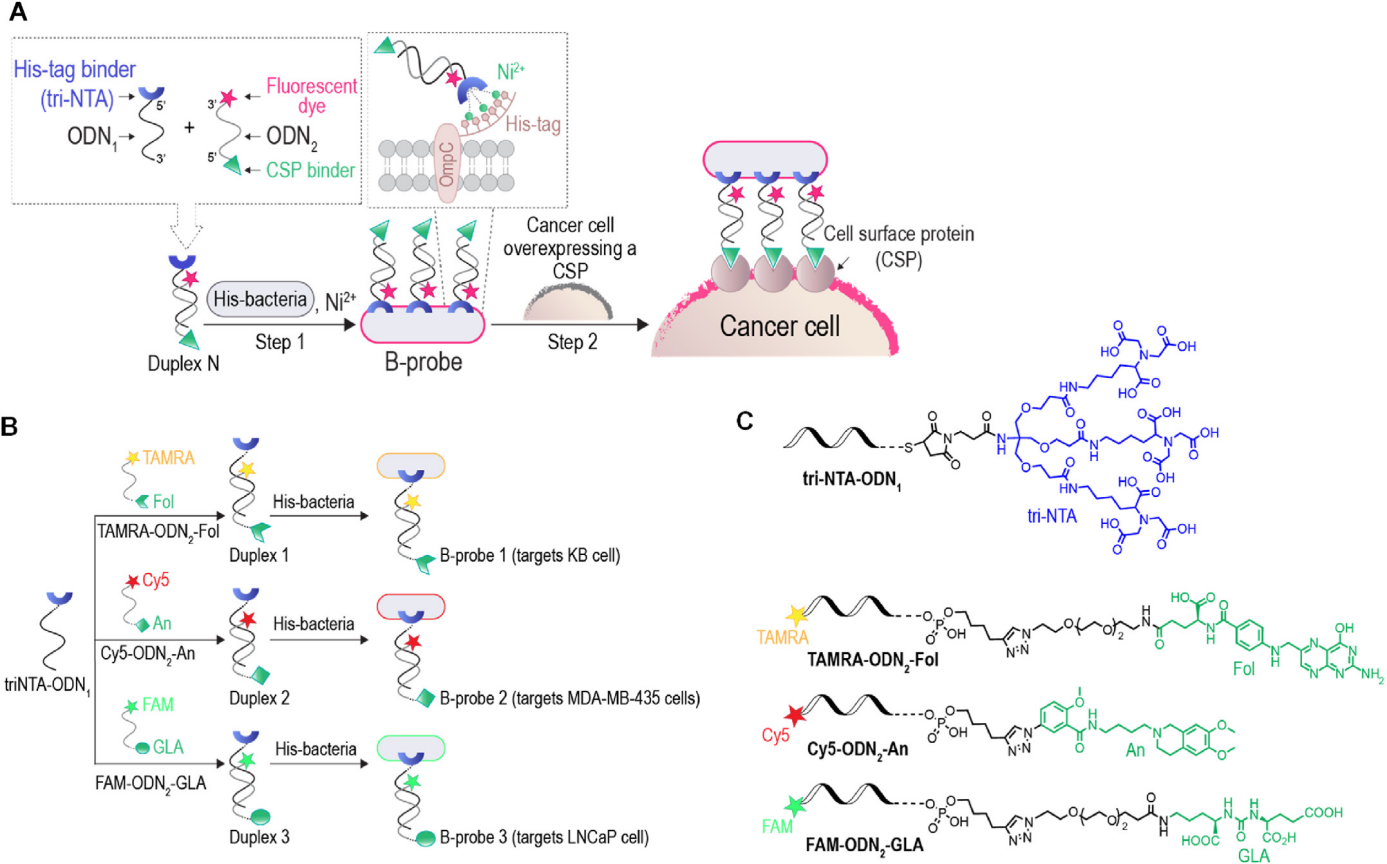
Design principles. (A) Schematic illustration of the way B-probes are created and utilized. Incubating His-bacteria with a DNA duplex appended with a fluorophore and binders for a His-tag and an overexpressed CSP (duplex N) affords a B-probe (step 1) that can label a specific class of cancer cells (step 2). (B) Schematic overview of the formation of B-probes 1–3 from modified DNA duplexes 1–3. B-probes 1–3 were designed to label cervical, melanoma, and prostate cancer cells, respectively. (C) The structures of the modified ODNs used to generate duplexes 1–3.

Fig. 2A schematically illustrates our approach to create a new class of fluorescent probes that, similar to fluorescent Abs, would be available in a wide range of colors and would be able to selectively label diverse types of CSPs. According to our design, these bacterial probes (B-probes) could be created by non-covalently linking a highly expressed bacterial CSP, outer membrane protein C (OmpC), to DNA duplexes appended with a fluorophore and two protein binders (Fig. 2A, duplex N). One binder interacts with a hexahistidine tag (His-tag) that is artificially fused to the OmpC, whereas the other binds to cancer cell CSP. The high efficiency by which the B-probes labeled the cancer cells (Fig. 2A, step 2) can be attributed to two main factors: The first is the large number of small-molecule ligands (binders of cancer cell CSPs) covering the bacterial scaffolds. This enables the B-probes to engage in multivalent interactions with the cancer cells, resulting in high-affinity binding. The second factor is the large number of fluorophores decorating each bacterium, enabling individual B-probes to generate a strong emission signal.

One of the ODNs constituting these duplexes is tri-NTA-ODN_1_, namely, a 19-base-long ODN (ODN_1_) that is modified at its 5′ terminus with a tri-nitrilotriacetic acid (tri-NTA) unit. The tri-NTA group is connected to ODN by a linker consisting of five sequentially arranged oligoethylene glycol (OEG) spacers (see the Supporting Information). In the presence of nickel ions, this tri-NTA group strongly and selectively interacts with the His-OmpC of *E. coli* [35,57]. The other ODN is a complementary DNA strand (ODN_2_) appended at its 3′ with a fluorescent dye and at its 5’ with a small-molecule- based ligand (i.e., a CSP binder) that can interact with a specific CSP on a cancer cell. The ligand is linked to ODN via two consecutive OEG spacers (see the Supporting Information). We expected that incubating the His-bacteria with the modified duplex and Ni^2+^ ions would afford a B-probe (Fig. 2A, step 1) that can selectively label a specific type of cancer cells (Fig. 2A, step 2).

His-OmpC-expressing bacteria were selected to scaffold these probes, owing to the high expression of His-OmpC [58] and the large surface area of bacterial cells. In nature, this combination (a large surface area and a high CSP density) enables the natural bacteria to bind to a variety of host cells in a multivalent fashion. In our system, similar design principles were applied to endow the B-probes with the ability to engage in unnatural bacteria-cancer cell interactions.

Based on these design principles, we created three modified DNA duplexes (Fig. 2B, duplexes 1–3) intended to afford three distinct B-probes (Fig. 2B, B-probes 1–3); each of them can label a specific cancer cell with a unique emission color. Duplexes 1–3 were generated by hybridizing the same His-tag binding strand (Fig. 2B, tri-NTA-ODN_1_) with distinctly modified ODN_2*s*_, namely, TAMRA-ODN_2_-Fol, Cy5-ODN_2_-An, or FAM-ODN_2_-GLA. The modified ODN_2*s*_ bear distinct fluorescent dyes: FAM (Ex/Em: 490/520 ​nm), TAMRA (Ex/Em: 545/580 ​nm), or Cy5 (Ex/Em: 630/670 ​nm). In addition, they are appended with distinct CSP binding molecules: folate (Fol), anisamide (An), or glutamate urea (GLA), known to selectively bind to the folate receptor (FR*α*) [59], sigma receptor (SR) [60], or the prostate-specific membrane antigen (PSMA) [61], respectively. We hypothesized that these CSP binders would enable B-probes 1, 2, and 3 to selectively label KB cells (cervical cancer cells), MDA-MB-435 (melanoma cells), and LNCaP (prostate cancer cells) overexpressing FR*α*, SRs, and PSMA, correspondingly [[62], [63], [64]].

The design of B-probes 1–3 (Fig. 2B) highlights the high modularity of this technology, which should provide the means to create a wide range of B-probes from the same His-tag binding strand (tri-NTA-ODN_1_) and His-OmpC-expressing bacteria. Specifically, it shows that diversification of the B-probes’ emission color and cancer cell targets can be achieved through a simple alteration of the fluorescent dyes and the small-molecule ligands that are linked to ODN_2_. The fact that a wide range of dyes can be incorporated in ODN_2_ during the automated DNA synthesis (by the proper selection of fluorescent phosphoramidites) further contributes to the high modularity of these probes. In this study, the TAMRA-ODN_2_-Fol, Cy5-ODN_2_-An, or FAM-ODN_2_-GLA was prepared simply by conjugating commercially available ODN_2_ derivatives (fluorophore- and alkyne-modified ODN_2*s*_) to distinct azide-modified small-molecule ligands, using the copper catalyzed ‘click’ chemistry (Scheme S5, Supporting Information).

### 2.2 Fluorescent labeling of cancer cells and tissues with the 1st generation B- probes

**Fig. 3:**
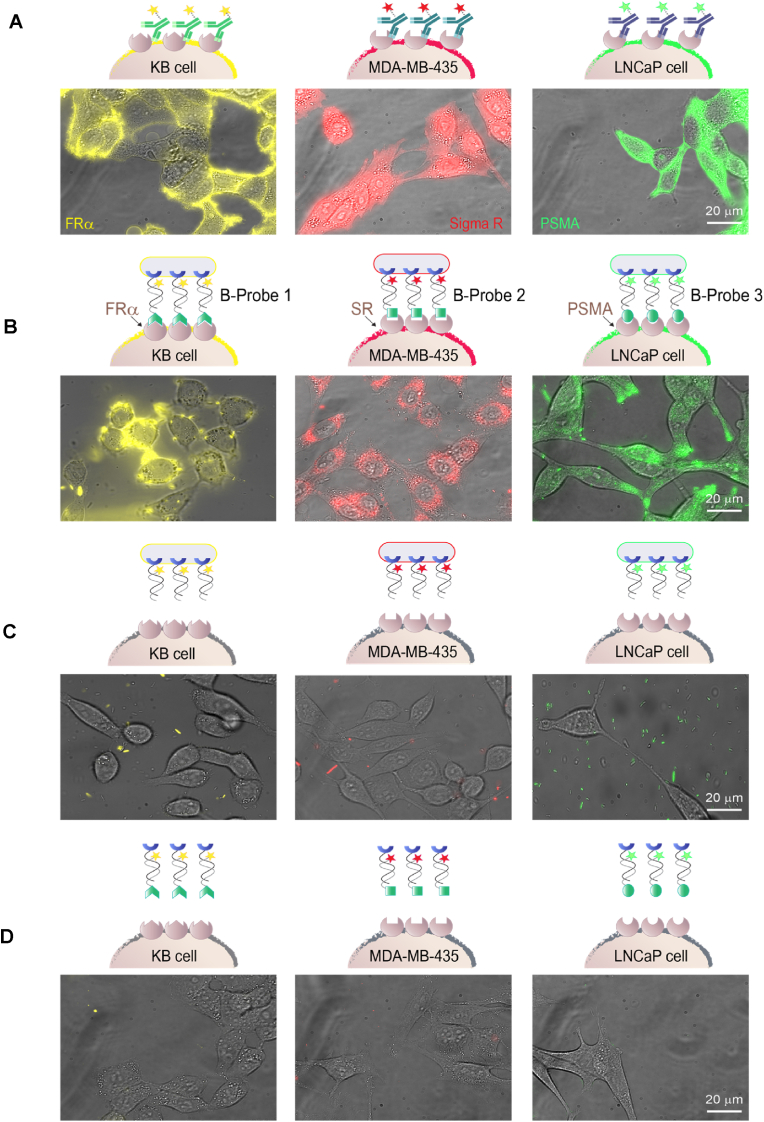
Merged bright-field and fluorescence images of KB cells (left), MDA-MB-435 ​cells (middle), and LNCaP cells (right) following incubation with (A) PE-anti-folR Ab (left), Alexafluor647-anti-sigmaR1 Ab (middle), and Alexafluor488 -anti-PSMA Ab (right), (B) B-probe 1 (left), B-probe 2 (middle), and B-probe 3 (right), (C) Bacteria linked to duplexes lacking the CSP binders, namely, duplexes generated from tri-NTA-ODN_1_ and TAMRA-ODN_2_ (left), Cy5-ODN_2_ (middle), or FAM-ODN_2_ (right), (D) 500 ​nM duplex 1 left), duplex 2 (middle), and duplex 3 (right) in the absence of His-bacteria. Cancer cells were subjected to the same incubation time and number of washes.

Before testing the ability of B-probes 1–3 to identify KB, MDA-MB-435, and LNCaP cells according to their unique CSP markers (FR*α*, SRs, and PSMA, respectively), we first labeled these CSPs with primary antibodies bearing different fluorophores (Fig. 3A and Fig. S1). As expected, the PE-anti-FolR Ab labeled the KB cells, the Alexafluor-647- anti-SigmaR Ab labeled MDA-MB-435 melanoma, and the Alexafluor-488-anti-PSMA Ab labeled the LNCaP cells. The results show that, as anticipated, each antibody selectively labeled one type of cancer cell (Fig. S1, Supporting Information) indicating the low or no expression of each CSP target in the other two cell lines. Another reason for performing this IF labeling experiment is to demonstrate how effective CSP labeling should look like. To determine whether efficient fluorescent cell labeling could also be obtained with the newly developed B-probes, we subjected the three cancer cell types to B-probes 1–3 (Fig. 3B). In this experiment, each cell type (KB, MDA-MB-435, or LNCaP) was incubated separately with each of the B-probes for 20 ​min, washed, and imaged using a fluorescence microscope. As negative controls, the cancer cells were also imaged following incubation with bacteria linked to duplexes that lack the CSP binders, namely, duplexes generated from tri-NTA-ODN_1_ and TAMRA-ODN_2_, Cy5- ODN_2_, or FAM-ODN_2_ (Fig. 3C). In addition, the cancer cells were imaged in PBS (Fig. S2) or following incubation with duplexes 1–3 in the absence of the His-bacteria (Fig. 3D). Inspecting the fluorescence images revealed that cancer cell labeling was only achieved when the cells were treated with the B-probes, namely, with His-bacteria decorated with fluorescent DNA duplexes bearing the specific CSP binders (i.e., Fol, An, or GLA) (Fig. 3B). The images also show that in the presence of cancer cells, it is often impossible to visualize intact bacteria on the cell membrane, indicating possible endocytosis (see section 2.4). The fact that no labeling was achieved in the absence of CSP binders (Fig. 3C) indicates the selectivity of the system, namely, that the labeling does not result from non-specific B-probe-cancer cell interactions. Moreover, the inability of duplexes 1–3 to label the cancer cells in the absence of bacteria (Fig. 3D) shows that the bacterial scaffold is essential for obtaining multivalent interactions with the CSP targets, as well as for enhancing the emission signal by integrating a large number of fluorophores into a single probe. To confirm the fact that, unlike fluorescent Abs, B-probes bind to CSPs at their small-molecule binding sites, we also showed that the KB-cells are not labeled with B-probe 1 following incubation with folic acid (1 ​μM) (Fig. S3, Supporting Information). This experiment in which the B-probe was displaced by a small-molecule ligand also indicates the potential of using the B-probe method to screen for small-molecule CSP agonists or antagonists, something that cannot be achieved with fluorescent Abs.

**Fig. 4:**
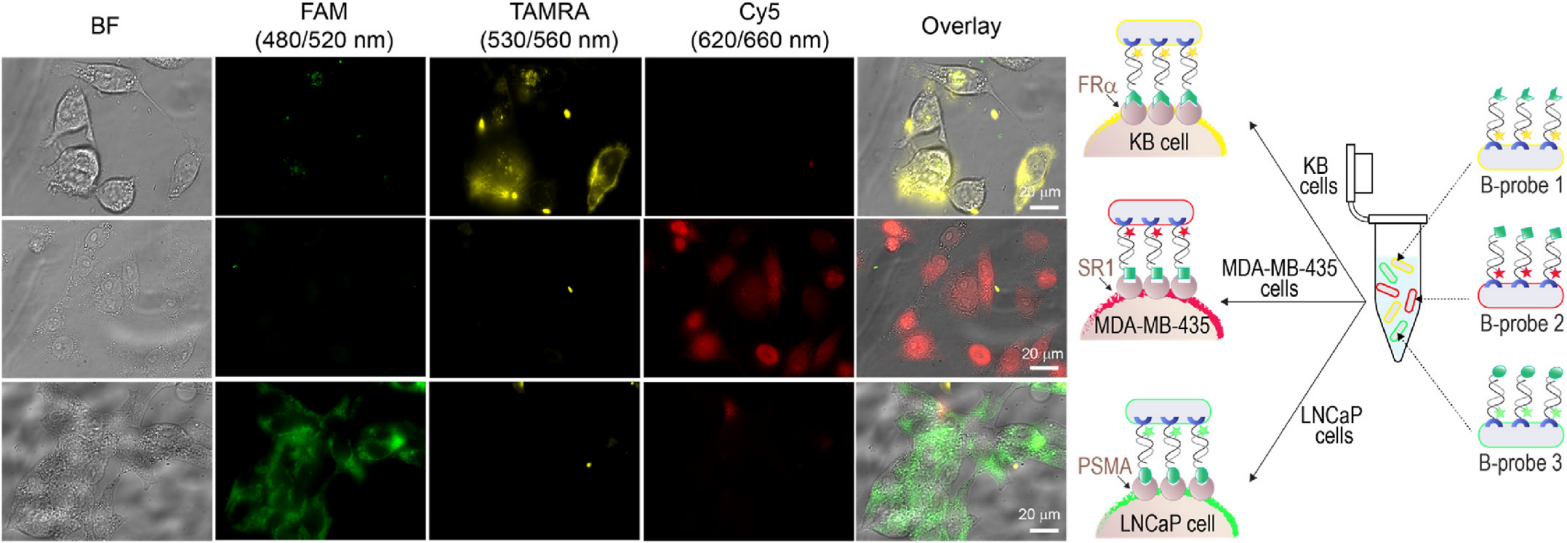
Selective labeling of cancer cells with a B-probe mixture. Right: Schematic illustration of an experiment in which a mixture of B-probes 1–3 was used to identify distinct cancer cell types, namely, KB cells (top), MDA-MB-435 ​cells (middle), and LNCaP cells (bottom). Left: The corresponding bright-field and fluorescence images of the labeled cells and their overlay. The cells were imaged using excitation and emission filters suitable for detecting each B-probe type.

A more efficient way to differentiate between the cancer cell types could be to subject the cells to a homogeneous mixture of B-probes 1–3 (Fig. 4, right). This may reduce incubation and washing steps, as well as minimize the number of samples needed for analysis. Because the fluorescent DNA duplexes are non-covalently bound to the His-bacteria, one may expect that combining different B-probes in a single solution would lead to an exchange of duplexes, which would lead to a mixed labeling of each cancer cell. Our expectation that such mixed labeling will not occur is based on a previous analysis showing that mixing His-bacteria covered with distinct fluorescent duplexes does not lead to the formation of new populations of labeled bacteria [35]. Fluorescence imaging of each cell type (under excitation and emission filters suitable for detecting FAM, TAMRA, and Cy5) following its incubation with the B-probe mixture (Fig. 4, left) revealed that labeling with the mixture did not lead to cross-reactivity. For example, the melanoma cells were primarily labeled in far-red, owing to the selective attachment of the Cy5-bearing B-probe (B-probe 2). Here, the yellow (TAMRA) and green (FAM) emitting B-probes 1 and 3 were washed off.

**Fig. 5:**
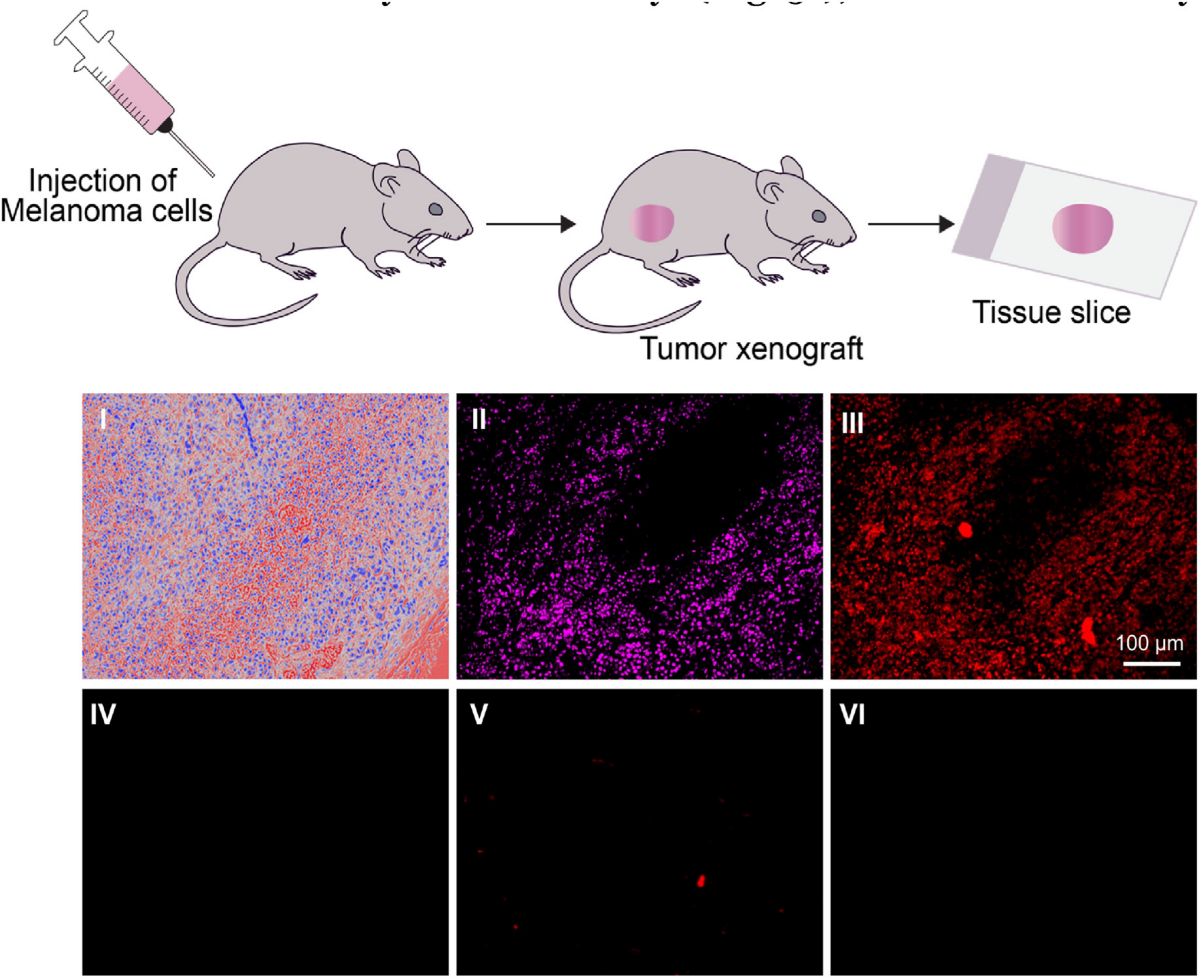
Bright-field (I) and fluorescence images (II-VI) of FFPE tissue sections from the MDA-MB-435 xenograft mouse model, following incubation with (I) Hematoxylin and Eosin stain, (II) Alexafluor647-anti- SigmaR1 Ab, (III) B-probe 2, (IV) Alexafluor647-IgG2b isotype control, (V) a derivative of B-probe 2 that lacks the CSP targeting unit (i.e., An), and (VI) duplex 2.

Similarly, the KB cells and LNCaP were exclusively labeled with B-probes 1 and 3, respectively. The specific labeling by the mixture further indicates the selectivity of the B-probes toward their targets, as well as the potential to use them in multiplexed labeling, something that is currently achieved with mixtures of fluorescent Abs [65]. The selectivity, target variability, as well as the ability to combine distinct B-probes in a single solution (Fig. 3, Fig. 4) indicate some similarities between the B-probe labeling method and IF. In addition to labeling proteins in cells, an essential application of IF lies in analyzing tissues obtained from biopsies [66,67]. The Ab-based fluorescence-staining patterns, which generally result from the labeling of overexpressed CSPs, can be used to diagnose the type of tumor, as well as determine to what extent it has spread [68]. To further highlight the resemblance between the B-probe method and IF, we set out to compare the *ex-vivo* tissue-labeling pattern obtained with fluorescent Abs and B-probes (Fig. 5). To this end, MDA-MB-435 Cell-Derived Xenografts (CDX) were induced in nude mice. After 4 weeks, the tumor (0.8 ​cm^3^) was extracted and processed into FFPE (Formalin-Fixed Paraffin- Embedded) blocks. Sections of 6 ​μm thickness from the FFPE tissue blocks were used for the staining experiments. One sample was stained with hematoxylin and eosin dye (Fig. 5I), which is commonly used in histology to localize nuclei and other cytosolic and extracellular proteins. The other samples were labeled with Alexafluor647-anti-SigmaR1 Ab or B- probe-2 (for details, see Fig. S4, and the Supporting Information) following the standard staining protocols. Fluorescence imaging of the Ab-treated slides (Fig. 5II) revealed that, as expected, the fluorescent Ab clearly stained the regions bearing antigen. Remarkably, despite the notable structural differences between fluorescent Abs and B-probes, staining the tumor tissue sample with B-probe 2 led to the generation of an almost identical fluorescence pattern (Fig. 5III). To validate the specificity of tissue labeling, we also showed that the tissue was not labeled following incubation with Alexafluor-647 conjugated normal mouse IgG2b isotype control (Fig. 5IV) or with His-bacteria covered with a duplex that lacks the A unit (Fig. 5V). Additionally, as was observed with the cell-labeling experiments (Fig. 3D), incubation of the cancer tissue with duplex 2 did not result in fluorescence labeling (Fig. 5VI).

### 2.3 Development of *2*nd generation B-probes that can produce stronger emission signals

The efficiency by which fluorescent probes label their protein targets not only depends on their binding affinity but also on the intensity of the emission signal that they can produce [69]. Because the fluorescence signal generally depends on the concentration of fluorophores in the medium, a rational approach to enhance the emission of CSP binding agents could be to link them to multiple fluorophores. Multi-dye conjugation, however, is generally unsuitable for improving the labeling efficiency of fluorescent Abs (Fig. 1B) or synthetic probes (Fig. 1C). The problem in using this method to enhance the emission of fluorescent Abs is that the fluorophores are generally non-specifically conjugated to lysine side chains on the Ab's scaffold (Fig. 1B). Hence, linking an Ab to more than a few dyes is expected to modify its antigen binding site, which will reduce its binding affinity. In contrast to Abs, synthetic probes (Fig. 1C) can be modified at well-defined positions, not at their protein-binding site. However, integrating several fluorophores into a single probe is synthetically challenging [12] and it could also lead to steric hindrance that would disrupt their interaction with the target protein. An additional problem that could be encountered when linking multiple dyes to both systems (i.e., fluorescent Abs and synthetic probes) is self-quenching that often occurs when fluorophores are located in proximity to each other [70].

**Fig. 6:**
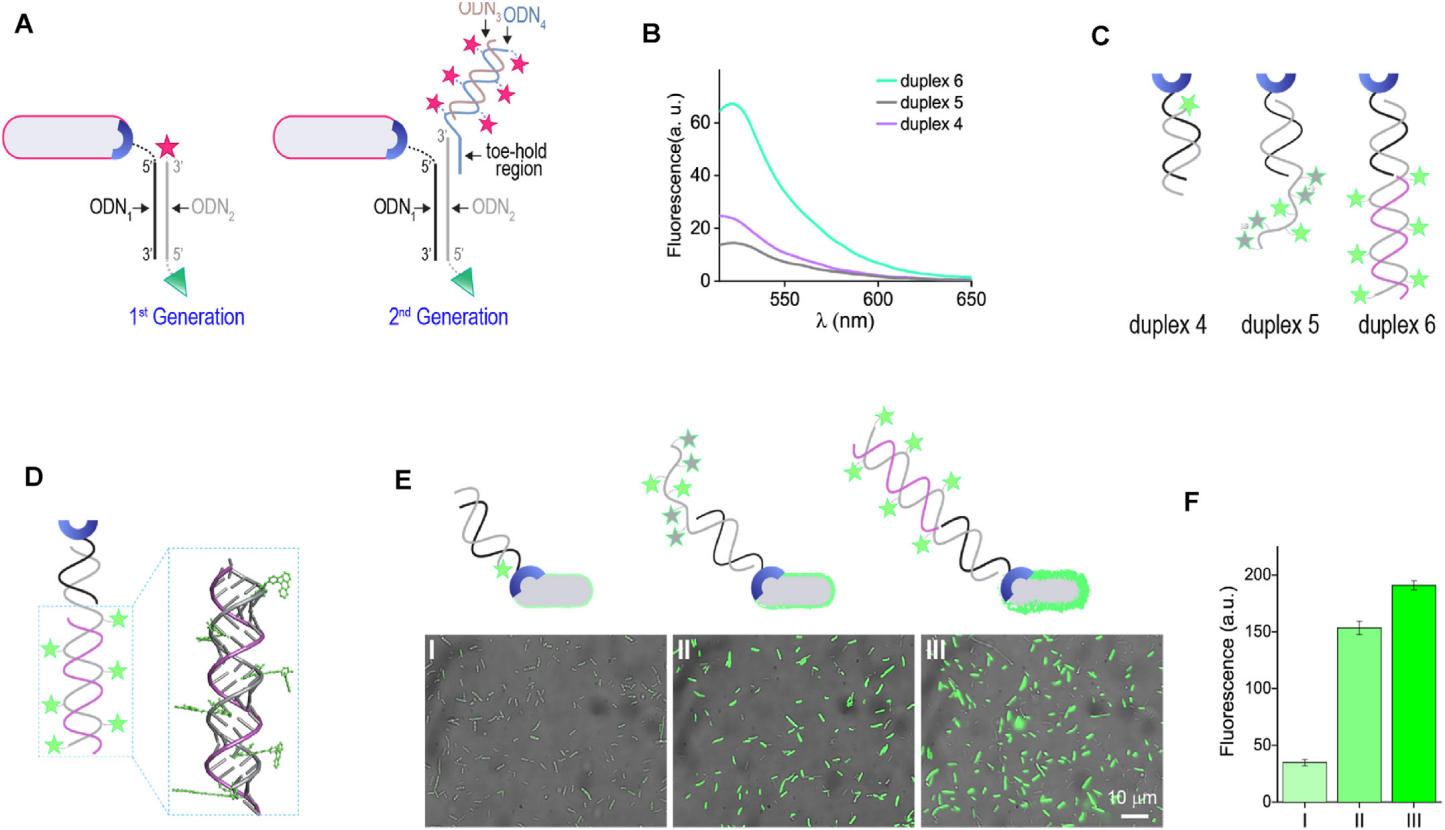
Studies towards the development of *2*nd generation probes. (A) Schematic illustration of the structure of the 1st (left) and the *2*nd generation (right) B-probes, which were designed to produce stronger emission signals. (B) Emission spectra generated by duplexes 4, 5, and 6. (C) Pictorial representation of these duplexes. (D) Model of the multi-dye-modified domain of duplex 6 shows that the six fluorescein molecules are spatially separated. (E) Merged bright-field and fluorescence images of His-bacteria labeled with duplex 4 (I), duplex 5 (II), and duplex 6 (III). (F) The corresponding average fluorescence intensity values generated from these labeled bacterial cells.

It occurred to us that by using the structural programmability of the B-probes’ DNA duplexes, it should be possible to create brighter B-probes in which the multiple fluorophores neither interact with each other nor hinder the probe-protein interactions (Fig. 6A, right). Specifically, we anticipated that brighter B-probes (Fig. 6A-right, *2*nd generation B-probes) could be obtained by replacing the single fluorescent dye incorporated in the duplexes constituting the *1*st generation probes (Fig. 6A, left) with a double-stranded DNA (dsDNA) bearing multiple fluorophores (Fig. 6A, right). Notably, the fluorophores in this duplex are spatially separated; hence, they cannot self-quench. To facilitate the preparation of the *2*nd generation B-probes, the multi-dye-modified dsDNA is attached to ODN_2_ via hybridization of two complementary hanging strands (or toe-holds). This should enable one to change the type and number of dyes simply by preparing a new, multi-dye-modified dsDNA from ssDNA building blocks (ODN_3_ and a multi-dye-modified ODN_4_) that can be readily synthesized on an automated DNA synthesizer.

Prior to creating the proposed *2*nd generation B-probes (Fig. 6A, right), we first used a model system (Fig. 6B–F) to evaluate two key hypotheses underlying our design. The first hypothesis is that fluorescence quenching resulting from dye-dye contacts could be prevented by controlling the orientation of the fluorophores on a multi-dye-modified DNA duplex. The second assumption is that His-bacteria decorated with such a duplex would emit more strongly than His-bacteria decorated with a duplex containing a single dye. To test the first hypothesis, we recorded the emission spectra of three modified DNA duplexes (Fig. 6B) whose structures are schematically depicted in Fig. 6C. Specifically, we compared the emission generated by a duplex that bears a single FAM dye (Fig. 6C, duplex 4) to the emission of two duplexes appended with six FAM units (Fig. 6C, duplexes 5 and 6). In duplex 5, ODN_2_ was elongated with 26 base-long strand containing six FAMs dyes. Duplex 6 was generated by hybridizing duplex 5 with a DNA strand that is complementary to the elongated sequences (ODN_3_). Because a right-handed *β*-helix has categorically 10 base pairs per turn, we expected that placing the dyes at positions i and i+5 would make the neighboring FAMs on duplex 6 project at distinct orientations. Inspecting the spatial orientation of the dyes (with BIOVIA Discovery Studio Visualizer) supported this assumption (Fig. 6D), as well as showed that FAM dyes located at positions i and i+10 should not interact with each other owing to the large distance between them. Inspecting the emission spectra generated by duplexes 4 and 5 (Fig. 6B) revealed that, despite having five additional FAMs, duplex 5 did not exhibit enhanced fluorescence when compared to duplex 4. In fact, a twofold reduction in emission was observed with respect to duplex 4, indicating a strong self-quenching effect. Duplex 6, on the other hand, in which the neighboring FAMs are separated, generated the strongest emission with a three-fold enhancement in fluorescence over duplex 4.

To determine whether the enhanced emission of duplex 6 would enable it to better label the His-bacteria, fluorescence images of the His-bacteria following incubation with 100 ​nM of duplexes 4–6 (in the presence of Ni^2+^) and washing were taken (Fig. 6E) and the average intensities of the fluorescence signals generated by individual bacterial cells were quantified (Fig. 6F). The results show that, as expected from the duplexes’ emission spectra (Fig. 6C), the His-bacteria were most effectively labeled with duplex 6. Interestingly, although the emission intensity of duplex 5 in solution was lower than that of duplex 4, it better labeled the His-bacteria than did duplex 4 (Fig. 6E, middle vs. left). This indicates that the binding of duplex 5 to the His-bacteria disrupts the undesired dye-dye interaction, presumably due to non-specific interactions with the bacterial cell surface. The fact that none of the duplexes labeled the His-bacteria in the absence of Ni^2+^ (Fig. S5, Supporting Information) confirms that duplex elongation and modification with additional dyes does not disrupt the specificity of such systems, namely, the binding of duplexes 5 and 6 to the bacterial His-OmpC is mediated by the highly selective interaction between the tri-NTA- Ni^2+^ complex and the His-tag.

**Fig. 7:**
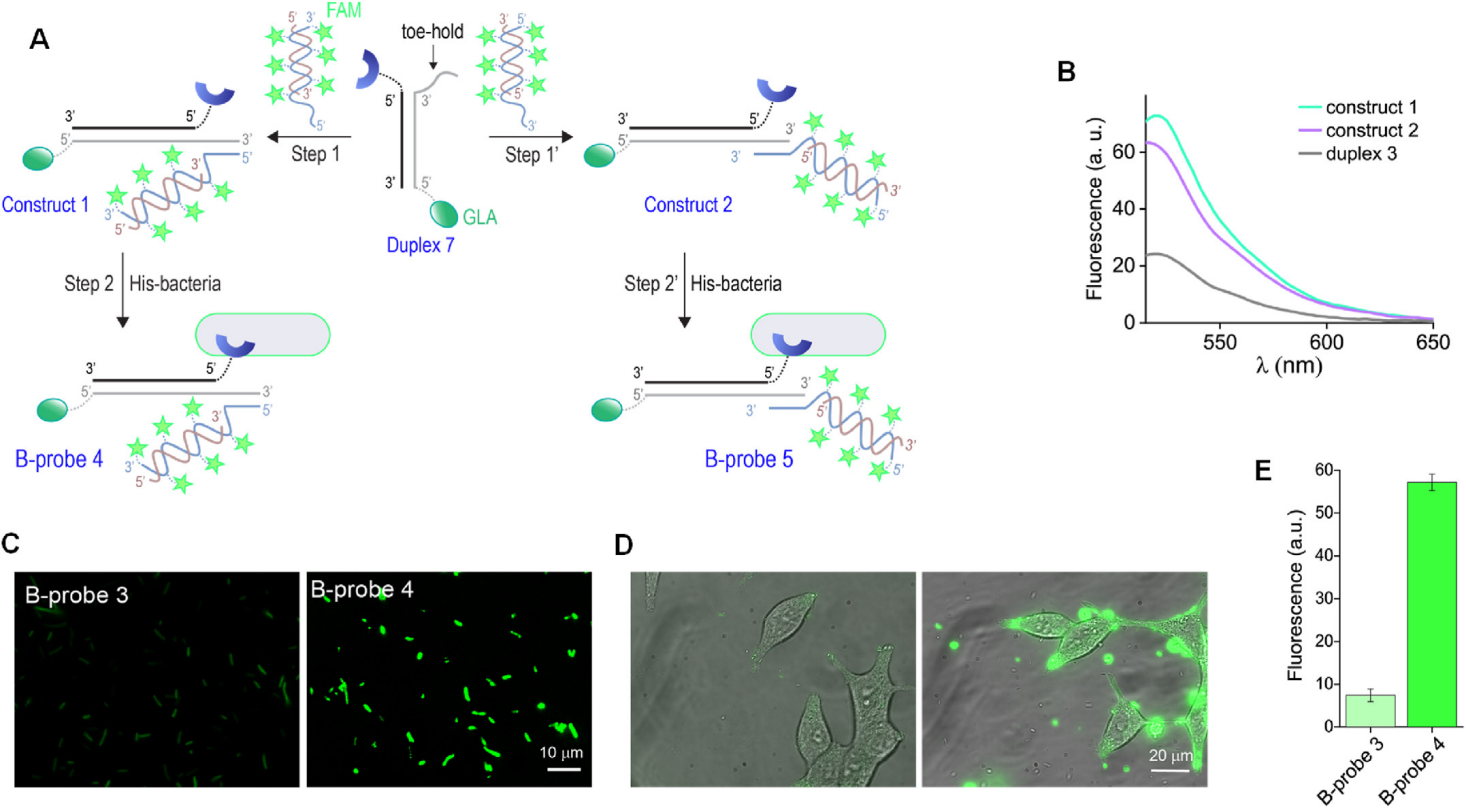
Development of brighter, *2*nd generation B-probes, intended to label cancer cells with higher efficiency. (A) Schematic representation showing the assembly steps used to form B-probes 4 and 5. (B) Emission spectra of duplex 3 (gray line), construct 1 (green line), and construct 2 (violet line) measured under an excitation wavelength of 495 ​nm. (C) Fluorescence images of His-bacteria following incubation with 100 ​nM of duplex 3 (B-probe 3, left) or construct 1 (B-probe 4, right) in the presence of Ni^2+^ (500 ​nM). (D) Merged bright-field and fluorescence images of LNCaP cells post-labeling with B-probe 3 (left) or B-probe 4 (right). (E) The corresponding average fluorescence intensity values. (For interpretation of the references to colour in this figure legend, the reader is referred to the Web version of this article.)

After demonstrating the feasibility of creating brighter chemically modified His-bacteria (Fig. 6E and F), we set out to determine whether similar design principles could be used to develop the brighter, *2*nd generation B-probes (Fig. 6A, right). Such probes should be able to label cancer cells more effectively than the *1*st generation B-probes (Fig. 6A, left). To this end, we created B-probes 4 and 5 (Fig. 7A). These *2*nd generation B-probes were intended to label prostate cancer cells with greater efficiency than B-probe 3 (Fig. 2B) whose DNA duplexes are appended with a single FAM dye. B-probes 4 and 5 were prepared as follows (Fig. 7A):

First, a DNA duplex integrating a tri-NTA unit, a GLA functionality, and a toe-hold was created (Fig. 7A, duplex 7); to this, another duplex appended with six FAM dyes and a complementary toe-hold was attached (Fig. 7A, step 1 or 1′) to afford DNA construct 1 (step 1) or 2 (step 1′) whose six FAM-appended duplex project to different orientations. The reason for altering the directionality of the multi-FAM duplex is to decrease the chance that it would interfere with the binding of the tri-NTA unit to His-OmpC. This change in directionality was achieved simply by altering the position of the hanging strand of the multi-FAM duplex from the 3′ to the 5’ terminus. After confirming the efficient self-assembly of DNA constructs 1 and 2 by gel electrophoresis (Fig. S6, Supporting Information), their emission spectra were recorded (Fig. 7B) and compared to the emission generated by single FAM-appended duplex 3 previously used to create the *1*st generation B-probe 3 (Fig. 2B and C). The measurements (Fig. 7B) showed that the fluorescence generated by 100 ​nM of constructs 1 and 2 is 3–3.5-fold larger than the emission of duplex 3 under the same concentration. As expected from these emission differences, fluorescence imaging of B-probe 3 (Fig. 7C, left), B-probe 4 (Fig. 7C, right), and B-probe 5 (Fig. S7, Supporting Information) showed that the *2*nd generation B-probes (B-probes 4 and 5) are 5-6-fold brighter than the *1*st generation B-probe 3. The fluorescence images also showed that constructs 1 and 2 afforded B-probes (B-probes 4 and 5) with similar brightness (Fig. S7, Supporting Information). Therefore, only one of them (B-probe 4) was used in the next experiments in which we used B-probe 4 to label prostate cancer cells (Fig. 7D and E).

### 2.4 Using the *2*nd generation B-probes to improve cancer cell labeling and to track B-probe internalization

**Fig. 8:**
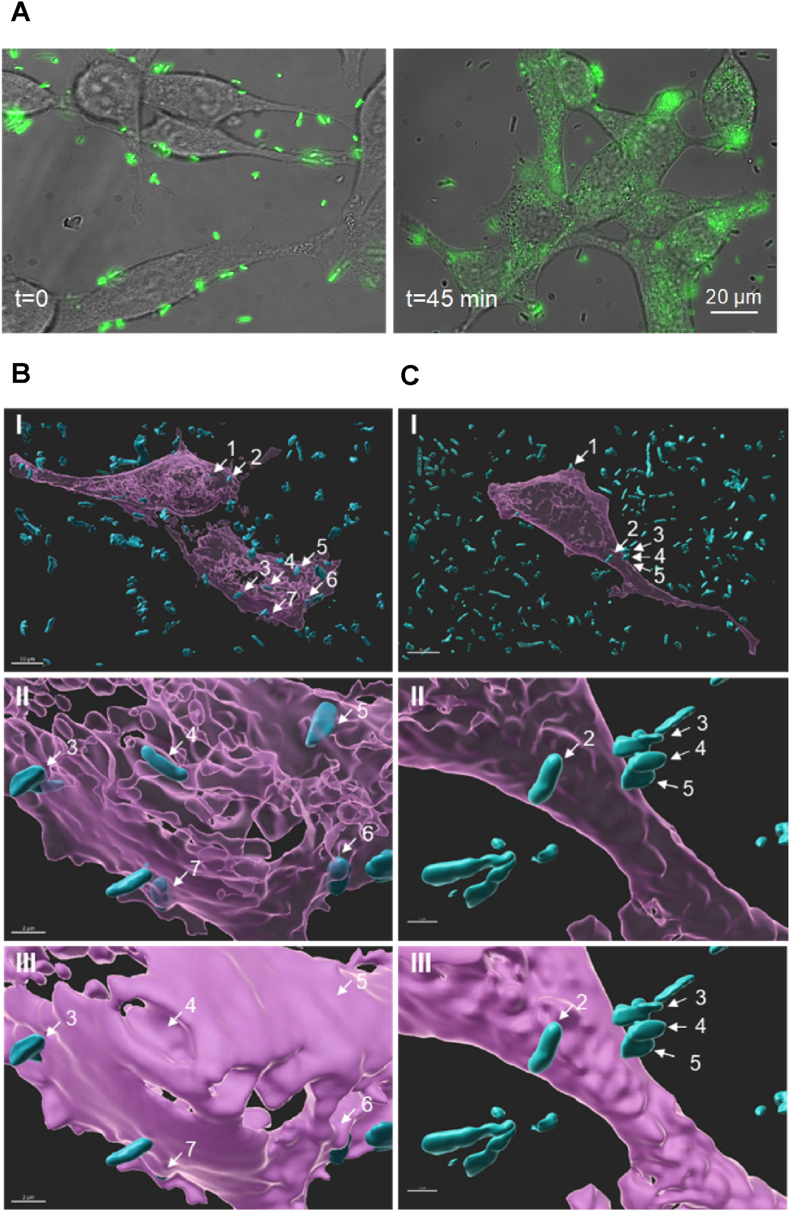
B-probes’ internalization into cancer cells. (A) LNCaP cells labeled with B-probe 3 ​at time t ​= ​0 (left) and t ​= ​45 ​min (right). (B) (I) Co-imaging of B-probe 4 (cyan) and LNCaP cells expressing mCherry-labeled membrane protein (pink). (II) zoom-in image, where the LNCaP cell membrane is visualized as transparent. (III) The same image in which the LNCaP cell membrane is viewed in an opaque mode. The images show that bacteria 3–7 are either entirely or partially engulfed in the cell. (C) Co-images obtained from a negative control experiment in which the same LNCaP cells were incubated with a derivative of B-probe 4 that lacks the GLA unit. (For interpretation of the references to colour in this figure legend, the reader is referred to the Web version of this article.)

To determine whether the stronger fluorescence generated by the *2*nd generation probes would enable them to label cancer cells more effectively than the *1*st generation probes, prostate cancer cells (LNCaP cells) were imaged following incubation with B-probe 3 (Fig. 7D, left) and B-probe 4 (Fig. 7D, right). Quantification of the emission signals (Fig. 7E) shows that, as expected from the stronger emission of the *2*nd generation B-probes, LNCaP cells subjected to B-probe 4 exhibited ∼6-fold enhancement in their emission when compared to LNCaP cells labeled with B-probe 3. After demonstrating more efficient labeling of cancer cells with the *2*nd generation probe 4, we aimed to determine whether the multiple fluorophores incorporated in its DNA duplex would facilitate imaging the chemically modified bacteria over time. An interesting phenomenon that we observed when imaging the *1*st generation B-probes (B-probes 1–3) post-incubation with cancer cells is that initially, intact fluorescently labeled bacteria (B- probes) are visualized on the surface of the cancer cells (Fig. 8A, left and Fig. S8), whereas after ∼45 ​min, large domains in the cancer cells become fluorescent (Fig. 8A, right and Fig. 3B). A probable explanation for this is a receptor-mediated endocytosis, which has been shown to induce the internalization of various small molecules and nano-carriers to these cells [[73], [72], [71]]. Such internalization and the consequent dissociation or degradation could also explain the uneven labeling of some cells. However, when trying to follow the *1*st generation B-probe-3 after binding to LNCaP cells, it was impossible to obtain clear images (Fig. S9, Supporting Information). A plausible explanation for the loss of a fluorescence signal is photobleaching that FAM dyes undergo under continuous illumination [74]. Therefore, we expected that a possible solution to this problem could be to repeat this imaging experiment with B-probe 4, which has a larger number of FAM dyes. This should make it less susceptible to photobleaching and thus, enable us to track the events that follow the initial binding of B-probe 4 to prostate cancer cells. Fig. 8B and the corresponding Supplementary movie (Fig. S10 and the Supplementary movie, Supporting Information) show the results of an experiment in which we imaged B-probe 4 in real time following incubation with LNCaP cells. To visualize the positioning of the bacterial probe (B- probe 4) with respect to the cancer cell membrane, LNCaP cells were transiently transfected with a plasmid encoding a mCherry CaaX-HRas protein, which resulted in the labeling of the boundary of the plasma membrane. Images were acquired using a live cell imaging system (Zeiss Cell Discoverer 7) with a high NA (1.2) objective, suitable for collecting Z stacks and producing 3D rendering of the cell. Co-imaging of B-probe 4 and the cancer cells’ membrane (Fig. 8B and S11) shown in transparent (Fig. 8B, I, and II) and opaque membrane masks (Fig. 8B, III) under ambient conditions (37 ​°C, 5% CO_2_) indicates that the bacteria imaged over the cancer cells are either partially (bacteria 3 and 6) or entirely (bacteria 4, 5, and 7) engulfed in the cell. As a control, we repeated this experiment with a derivative of B-probe 4, which lacks the GLA unit (Fig. 8C). The fact that under these conditions, bacterial internalization into the LNCaP cancer cells was not observed indicates that the targeting unit of B-probe 4 (GLA) mediates a selective interaction between B-probe 4 and prostate cancer cells, as well as the consequent receptor-mediated endocytosis. The ability to image both B-probe 4 (Fig. 8B) and its non-targeting derivative (Fig. 8C) under continuous irradiation is another important outcome of this experiment, showing how an increase in the number of fluorescent dyes provides a possible means to minimize undesired photobleaching effects.

## 3 Conclusion

In nature, bacterial infections are primarily mediated by the multivalent interaction between bacterial and host cell CSPs [75]. The high efficiency of this recognition event is attributed to the large surface area of the bacterial cells and the high density of the targeting CSPs [76] (e.g., adhesins [77]). Here, we have shown that these properties make living bacteria an excellent scaffold for creating fluorescent probes able to effectively label specific types of cancer cells and tissues. The high efficiency by which the B-probes labeled the cancer cells can be attributed to the large number of small-molecule ligands and fluorophores covering the bacterial scaffolds. The multiple ligands enable the B-probes to engage in strong multivalent interactions with the cancer cells whereas the multiple fluorophores, enable the B-probes to generate a strong emission signal. This combination of multiple targeting elements and multiple fluorescent dyes not only makes B-probes superior to most monovalent fluorescent probes (Fig. 1C)—it also enables using B- probes in applications generally achieved with IF. We have shown, for example, that, similar to CSP imaging with fluorescent Abs, B-probes can label CSPs overexpressed in cancer cells (KB, MDA-MB-435, and LNCaP cells). The ability to label these three cell types using a mixture of distinct B-probes, further demonstrates the resemblance of this technology to IF, since it indicates the high selectivity of such systems and the potential to apply them in multiplexed detection. We have also shown that B-probes can be used to identify carcinogenic tissues, providing labeling patterns similar to the ones obtained with fluorescence Abs.

Although the experiments mentioned above highlight some similarities between the B-probe labeling method and IF, the design, structure, and operating principles underlying B-probes and fluorescent Abs are fundamentally different. One key difference is the way fluorescent Abs and B-probes are constructed. Whereas fluorescent Abs are generated through covalent conjugation of fluorophores to a few randomly distributed amino acid side chains, B-probes are generated through self-assembly processes involving Ni^2+^ complexation and DNA hybridization. One advantage of self-assembly is that it makes B- probes more amenable to modification than fluorescent Abs. Whereas modifying amino acid side chains of Abs with additional fluorophores is likely to disrupt their function, we have shown that brighter B-probes (the *2*nd generation B-probes) can be readily created by non-disruptively integrating multi-dye-containing DNA duplexes into their structures. By controlling the orientation of the dyes, we could ensure that adjacent dyes are spatially separated and consequently, that self-quenching of fluorescence is prevented. This enabled the *2*nd generation B-probes to better label cancer cells and it has also made them less susceptible to photobleaching. The ability to place the multi-dye-modified duplexes far from the His-tag binder or the CSP ligand is another important advantage of the B-probes over Abs, because it ensures that the addition of dyes does not disrupt the binding of the B-probes to their protein targets. The observation that the B-probes undergo internalization following a highly selective interaction with cancer cells is another interesting aspect of this study because it indicates the potential to apply some design principles underlying B-probes in bacterial therapy [*51*]. Specifically, it suggests the possibility of improving the targeting capabilities of therapeutic bacteria through a systematic, chemical modification of their membranes.

Another critical difference between B-probes and fluorescent Abs lies in the CSP domains that they target. Whereas B-probes target small-molecule binding sites of CSPs, Abs target the CSPs’ surfaces. This difference may open the way to using B-probes to label CSPs that do not have suitable Abs [78]. In addition, as was exemplified in the folate displacement experiment (Fig. S3, Supporting Information), it may enable using B-probes to screen for CSP agonists or antagonists. Although optimizing such probes for these purposes may require additional modifications of their structures, this work has shown that B-probes are exceptionally simple to manufacture and modify. First, we have shown that distinct B- probes can be formed from the same basic components. One is a self-replicating bacterial scaffold, whereas the other is a His-tag binding strand. Second, we have shown that most structural modifications, such as changing the type and number of dyes, can be readily obtained through a rational choice of commercially available phosphoramidites used in the automated DNA synthesis. Note that, unlike small-molecule or Ab-based probes, the large size of B-probes prevents using them for imaging CSPs at a spatial and temporal resolution. However, this study shows that in terms of CSP characterization and consequent diagnostic applications, this approach could complement the use of fluorescently labeled small molecules or Abs, as well as offer several potential benefits. Considering the structural programmability, we expect that new classes of probes for detecting additional types of cancer cells with higher efficiencies will be developed, which would extend the fluorescence tool box currently used to detect CSPs in native (non-engineered) cells.

## 4 Experimental section

### 4.1 Materials and methods

All solvents and reagents were obtained from commercial suppliers and used without further purification. Dry solvents were purchased from Sigma Aldrich. Deuterated solvents were purchased from Cambridge Isotope Laboratories, Inc. (Andover, MA). Aluminium-backed silica plates (Merck silica gel 60 F254) were used for thin layer chromatography (TLC) to monitor solution-phase reactions. The ^1^H NMR and ^13^C NMR spectra were recorded on a Bruker Advance 300, 400 or 500 ​MHz spectrometer. The chemical shifts are represented in ppm on the *δ* scale down field from TMS as the internal standard. The following abbreviations were used to describe the peaks: br-broad, s-singlet, d-doublet, t-triplet, td-triplet of doublets, q-quartet, quin-quintet and m-multiplet. Oligodeoxynucleotides (ODNs) were obtained from W. M. Keck Foundation Biotechnology at Yale University. The mass spectrum was recorded by Waters SYNAPT-XS Q-TOF High Resolution mass spectrometer with an electrospray ionization (ESI) interface in the negative ion mode within a mass range from 400 to 5000 ​*m*/*z*. Matrix-assisted laser desorption ionization time-of-flight (MALDI-TOF) mass spectrometry was performed on an AB SCIEX 5800 system, equipped with an Nd: YAG (355 ​nm) laser with a 1 ​KHz pulse (Applied Biosystems), at the Weizmann Institute of Science mass spectrometry facility. For small molecules, the analytical reversed phase high-performance liquid chromatography (RP-HPLC) analysis was performed on an Agilent Technologies 1260 Infinity quaternary pump LC system, equipped with a diode-array detector using a C_18_ column. Preparative HPLC was carried out using an Agilent 218 purification system, equipped with an autosampler, a UV–Vis dual wavelength detector, and a 440-LC fraction collector operating under OpenLab ChemStation software. The purification of oligodeoxynucleotides was carried out on a Waters 2695 separation module HPLC system with a 2994 photodiode array detector using either a Waters XBridgeTM OST C_18_ column (2.5 ​μM, 4.6 ​mm ​× ​50 ​mm) or an XBridgeTM OST C_18_ column (2.5 ​μM, 10 ​mm ​× ​50 ​mm). Oligodeoxynucleotide samples were desalted using illustra MicroSpin G-25 Columns (GE Healthcare) according to the supplier's instructions. Concentrations of the oligodeoxynucleotides were quantified based on their respective electronic absorption at 260 ​nm and the molar extinction coefficient of the oligodeoxynucleotide at this wavelength. For gel electrophoresis, 6 ​× ​DNA loading dye (Thermo Scientific) and PCR-25 bp or PCR-20 bp ladder (Sigma Aldrich) were used. The molecular modelling of oligonucleotides was carried out on BIOVIA Discovery Studio Visualizer 2019. MCherry-CaaX HRAS (Addgene plasmid # 108,886; http://n2t.net/addgene:108,886;-RRID:Addgene-108,886) was a kind gift from Professor Rob Parton (university of Queensland, Australia). KB cell lines were obtained from Prof. Ronit Satchi-Fainaro's group (Tel Aviv university, Israel) while LNCaP and MDA-MB-435 were obtained from stem cell core facility and advanced cell technologies, life sciences core facilities, Weizmann Institute of Science. These cell lines were screened negative for mycoplasma using a PCR-based assay (EZ-PCR mycoplasma detection kit, Biological Industries). Nude female mice were procured from Veterinary Resources, Weizmann Institute of Science. Fluorophore conjugated antibodies were purchased from Bio Legend and Santa Cruz Biotechnology. Cell images were acquired using an Olympus IX51 fluorescent microscope equipped with a U-MNIBA3 fluorescence filter cube (excitation and emission filters of 470–495 ​nm, and 510–550 ​nm, respectively), a U-MNG2 fluorescence filter cube narrow-band (excitation and emission filters of 530–550 ​nm, and 590 ​nm, respectively) and a U-MF2 fluorescence filter cube (excitation and emission filters of 620–660 ​nm, and 700–775 ​nm, respectively). Tissue samples were imaged with Leica LAS X inverted microscope. Fluorescence was measured using a BioTek synergy H4 hybrid multiwall plate reader, in black flat-bottom polystyrene NBS 384-well microplates (Corning).

### 4.2 Synthesis of the modified ODNs

The detailed experimental procedures for preparation of the modified ODNs including their characterizations are included in Supporting Information.

### 4.3 Bacterial strain and growth conditions

*E. coli* K-12 strain KRX (Promega) was used for OmpC expression. The details of the expression of 3 copies of hexahistidine-tag at the 7th loop of the OmpC have been described in our previously published paper [*35*]. Transformed bacteria with His-OmpC constructs were cultured to saturation in LB medium supplemented with ampicillin (100 ​μg/mL) at 30 ​°C. Next, pre-cultured cells were diluted 1:100 in fresh LB medium supplemented with ampicillin, and incubated at 30 ​^°^C. Once the cells reached the mid-exponential phase (OD_600_ ​≈ ​0.6), protein expression was induced by the addition of 0.1% Rhamnose and 20 ​μM isopropyl-b-d-1-thiogalactopyranoside (IPTG) and incubated at 30 ​°C while shaking for ​≈ ​18 ​h. Then, the bacterial cells were harvested by centrifugation at 6000 ​g for 4 ​min.

### 4.4 Preparation of B-probes and their fluorescence imaging

The bacterial cells were collected by centrifugation at 6000 ​g for 4 ​min. Pellets were washed twice with PBS ​× ​1 buffer and re-suspended in the same buffer to an OD_600_ of 0.3. To a 100 ​μL sample of the bacteria suspension, a pre-incubated sample of DNA (500 ​nM) and NiCl_2_ (2.5 ​μM) was added, and the cells were incubated at room temperature for 1 ​h. Then the sample was washed twice with PBS, re-suspended in 200 ​μL PBS, and placed on a glass-bottom dish (P35G-1.5-14-C; MatTek) pre-coated with poly-l-lysine (Sigma Aldrich) and left to adhere for 1 ​h. Finally, the bacterial cells were vigorously washed with PBS three times and imaged using an Olympus IX51 fluorescent microscope. The samples were imaged using 100 ​× ​objective lens.

### 4.5 Fluorescence emission measurements of various ODN duplexes

Samples of ODN duplexes 3–6 or constructs 1–2 (20 ​μM) and NiCl_2_·6H_2_O (100 ​μM) were mixed in PBS buffer (pH ​= ​7.2) and allowed to stand at room temperature for 30 ​min. Then each duplex was diluted in PBS to a final concentration of 100 ​nM, 60 ​μL of each sample was added to the black flat bottom corning 384 well plate and the emission spectra was immediately recorded. The fluorescence responses were measured using the excitation wavelength of 495 ​nm. These experiments were performed in triplicates.

### 4.6 Fluorescence imaging of bacteria-cell interaction

Cells (15,000 ​cells/well) were seeded onto glass bottom culture dishes (MatTek) and allowed to adhere overnight. MDA-MB-435 ​cells were seeded in MEM medium supplemented with 5% charcoal dextran-stripped calf serum, 1% non-essential amino acids, 1% l-glutamine, and 1% penicillin/streptomycin. KB cells were plated in folate-depleted RPMI supplemented with 10% FBS and 1% penicillin/streptomycin while LNCaP were maintained in RPMI, 10% FBS, 1% l-glutamine, and 1% penicillin/streptomycin. Following day, medium was removed, rinsed twice with PBS and then the cells were incubated with B-probes **1**–**4** in a CO_2_ incubator (5% CO_2_) at 37C for 20 ​min. Finally, the excess/unbound bacteria were gently washed off with PBS containing Ca^2+^ ​and Mg^2+^ (3 ​× ​200 ​μL) and the cells were imaged using a fluorescence microscope with 60 ​× ​objective lens. Negative control experiments were performed similarly using bacteria decorated with a duplexes generated from tri-NTA-ODN_1_ and DNA strands (lacking the corresponding CSP binders) namely, TAMRA-ODN_2_, Cy5-ODN_2_ or FAM-ODN_2_.

### 4.7 Displacement experiment with small molecule that disrupt the bacteria-cell interaction

KB Cells maintained in folate-depleted RPMI supplemented with 10% FBS and 1% penicillin/streptomycin were seeded (15,000 ​cells/well) onto glass bottom culture dishes (MatTek) and allowed to attach overnight. The next day, medium was removed, and cells were rinsed with PBS (200 ​μL ​× ​3) followed by the incubation of cells with 1 ​μM of commercial folic acid in 200 ​μL PBS (taken from a stock of 5 ​mM in DMSO) for 15 ​min at 37C, 5% CO_2._ Next, the folic acid solution was pipetted out and the resulting KB cells were incubated with B-probe **1** (prepared according to the procedure described in earlier section) for 20 ​min. Finally, the excess/unbound bacteria were gently washed off with PBS containing Ca^2+^ ​and Mg^2+^ (3 ​× ​200 ​μL) and the cells were imaged using a fluorescence microscope with 60 ​× ​objective lens.

### 4.8 Fluorescence imaging of different cancer cells interacting with a B-probe mixture

KB, MDA-MB-435 ​cells, and LNCaP cells were maintained in their respective media as mentioned in the previous section. Cells (15,000 ​cells/well) were seeded onto glass bottom culture dishes (MatTek) and allowed to adhere overnight. The medium was removed from the cells and rinsed twice with PBS. On the other hand, B-probes **1**–**3** (prepared as described in the earlier section) were mixed (3 ​× ​200 ​μL). A mixture of this bacterial sample (200 ​μL) was incubated separately with each cell line at 37 ​°C under 5% CO_2_ condition. After 20 ​min, the unbound bacteria were gently washed off with PBS containing Ca^+2^ and Mg^+2^ (3 ​× ​200 ​μL) and the cells were imaged using a fluorescence microscope and a 60 ​× ​objective lens.

### 4.9 Immunofluorescence procedure

KB, MDA-MB-435 ​cells, and LNCaP cells (15,000 ​cells/well) were separately seeded on glass-bottom dish (P35G-1.5-14-C; MatTek). LNCaP and KB cells were fixed with 4% PFA for 10 ​min and blocked for 1 ​h at 37C with blocking buffer (2% fetal bovine serum in PBS containing). Alexa Fluor 488 anti-human PSMA (BLG-342505, BioLegend) or PE anti-FolR (BLG-908303, BioLegend) were incubated with the LNCaP and KB cells, respectively, in 1:200 dilution in PBS at 0C for 30 ​min. MDA-MB-435 ​cells, were first fixed with 4% PFA for 10 ​min, permeabilized using 0.01% triton for 2 ​min, blocked for 1 ​h at 37C with blocking buffer (2% fetal bovine serum in PBS). Then the fixed cells were incubated with Alexa Fluor 647 anti-sigmaR1 antibody (sc-166,392 AF647, Santa Cruz Biotechnology Inc.) at 1:200 dilution in PBS at 0 ​°C for 30 ​min. Then, all the three antibody stained cell samples were washed twice with 200 ​μL of PBS and imaged using an Olympus IX51 fluorescent microscope using 60 ​× ​magnification using excitation/emission wavelengths of 470–495/510–550 ​nm, 530–550/590 ​nm or 620–660/700-775 ​nm for imaging LNCaP, KB and MDA-MB-435 ​cells, respectively.

### 4.10 Fluorescence imaging to study internalization of B-probe into cancer cells

LNCaP cells were cultured as described above in RPMI medium containing 10% FBS, 1% l-glutamine, and 1% penicillin/streptomycin. The cells were plated at 20,000 ​cells/well in an eight-well chamber slide (Cellvis Cat #C8-1.5H–N, CA, USA) and transfected 24 ​h later with mCherry-CaaX Hras using Lipofectamine 2000 (Life Technologies, Carlsbad, CA) according to the manufacturer's guidelines. Next day, the transfected LNCaP cells were washed twice with PBS (300 ​μL), and B-probe **3** or **4** was added to the cells. Z-stacks (0.31 ​μm) of selected cells expressing mCherry-CaaX were acquired on the Carl Zeiss Ltd., cell discoverer 7 microscope (CD7). A Plan-Apochromat 50 ​× ​/1.2NA objective with 2 ​× ​tube lens (effective magnification of 100 ​× ​) was used for image acquisition, and detection was done on a 14bit Axiocam 702 CMOS camera (Carl Zeiss Ltd.). Imaging was performed using a combination of two LED modules simultaneously: 470 and 590 ​nm wavelengths with bandpass emission filters: 412–433; 501–547 and 617–758. The CD7 chamber was set to 37 ​°C with injected 5% CO_2_. Images acquired from CD7 microscope were deconvolved using ZEN Blue 3.1 software using the “excellent slow constrained iterative algorithm” method. 3D-deconvolved images were then segmented and visualized in Imaris 9.8 (Bitplane) software. The bacteria and the membrane were segmented using the Imaris 3D surfaces module. After the segmentation, the membrane mask was shown once as transparent and once as opaque at the same image to show the bacterial entry into cell. To determine the internalization of B-Probes into cancer cells we used the default parameters for bacteria segmentation (the smoothing of 2 pixels, automatic threshold). For cells we used the smoothing of 8 pixels and a manual intensity threshold to ensure that they appear as a closed structure. We measured the distance from each bacterium [center] to the cell border by calculating the distance transform inside and outside the cell. When the inside-distance transform is calculated in Imaris, all voxels outside the cell get a zero value and voxels inside the cell get a value based on their distance to the cell border. For outside-distance transforms, all voxels inside the cell get a zero value and voxels outside the cell get a value that equals their distance to the cell border. We classified bacteria as either inside-bacteria or outside-bacteria based on the median value of the outside-distance transform.

### 4.11 Tissue sections preparation and staining

All animal experiments were conducted in accordance with approved institutional animal care and use committee (IACUC) protocols. CD1-nude mice were purchased from Envigo RMS (Israel) Ltd. Mice were housed and handled in a specific-pathogen-free, temperature-controlled (22 ​°C ​± ​1 ​°C) mouse facility on a reverse 12/12 ​h light/dark cycle, Animals were fed a regular chow diet *et libitum*. MDA-MB-435 ​cells (5 ​× ​10^6^ per mouse) were subcutaneously injected in the right flank of 6 week-old female CD1 nude mice. Once tumor reached a volume of approximately 750 ​mm^3^, mice were euthanized and tumours were extracted for analysis. After fixation with 4% PFA, the tissues were processed to paraffin embedded blocks, and 6 ​μm sections were taken for immunohistochemistry or bacteria/DNA staining. Before proceeding with immunohistochemistry or addition of bacteria, sections were de-paraffinized in xylene and treated with decreasing concentrations of ethanol. Post-fixation was done using ice-cold acetone for 7 ​min, and heat mediated antigen retrieval was performed in Tris/EDTA buffer (pH 9) for 10 ​min. All the sections were blocked using 20% normal horse serum (Vector Laboratories, CA, USA) and 0.2% Triton-X, for 90 ​min. After staining, the samples were imaged with Leica Mi8 microscope equipped with a motorized stage and a Leica DFC365 FX camera. Single ​× ​20 magnification images were tiled to receive a full scan of the tumor section.

### 4.12 Fluorescence immunohistochemistry

Tissue sections were incubated with Alexa 647 conjugated sigma receptor (sc-166,392 AF647, Santa Cruz Biotechnology, 1:50) or Alexa Fluor 647 conjugated normal mouse IgG2b (sc-24638, Santa Cruz Biotechnology, 1:50) at room temperature overnight under dark humidified chamber. Sections were washed 3 times with PBS for 5 ​min, covered using aqua-poly/mount coverslip (cat. no. 18606–20, Polysciences Inc.) and imaged.

### 4.13 Staining of tissue sections with bacterial probes

PAP pen (Cat. No. H-4000, Vector Laboratories, CA, USA) was used to create a hydrophobic boundary around tissue sections. The tissue sections were separately incubated for 1 ​h with 200 ​μL of the following samples: B-probe 2 and control B-probe (prepared by decorating His-bacteria with duplex tri-NTA-ODN_1_: Cy5-ODN_2_). After washing off the unbound *E. coli* with PBS buffer, the sections were covered using aqua-poly/mount coverslip (cat. no. 18606–20, Polysciences Inc.) and imaged.

### 4.14 Gel electrophoresis for analysing the constructs 1 and 2

The 2% agarose gels were prepared by mixing 2% (w/v) of agarose in 50 ​mL Tris/Borate/EDTA (TBE). The mixture was heated in microwave until a clear solution was obtained. To the agarose gel, 2 drops of ethidium bromide was added and the solution was poured in the mould and allowed to solidify by cooling. Individual samples of ODNs to be tested were prepared in Tris buffer (12 ​mM, pH 7.5, 137.5 ​mM LiCl) at a final concentration of 12 ​μM each. The 12 ​μM samples in a volume of 10 ​μL was mixed with 2 ​μL loading dye (6 ​× ​) such that final concentration of samples is 10 ​μM each for running the gel. Electrophoresis was carried out at 100 ​V for 90 ​min using a Mini-PROTEAN Tetracell system (Bio-Rad, CA). Gels were visualized with the ChemiDoc XRS imaging system (Bio-Rad, CA).

## Author contributions

P. K. P.: methodology, investigation, data acquisition, analysis, visualization, writing original draft-review & editing; N.E.: methodology; I. G.: methodology; L. F. A.: methodology; R. O.: methodology; O. G.: methodology; L.M.: methodology, writing–review & editing, project administration, funding acquisition; D.M.: conceptualization, writing–review & editing, project administration, funding acquisition.

## Declaration of competing interest

The authors declare that they have no known competing financial interests or personal relationships that could have appeared to influence the work reported in this paper.

## Data Availability

In our submission we included supporting information file.
