# Supplementary material for: Chemically programmable bacterial probes for the recognition of cell surface proteins
Source: Mater Today Bio. 2023 May 23;20:100669. doi: 10.1016/j.mtbio.2023.100669 (PMC10275978; doi:10.1016/j.mtbio.2023.100669)
Supplement: Multimedia component 1 [file mmc1.pdf]

Supporting Information

Chemically Programmable Bacterial Probes for the Recognition of Cell Surface Proteins

Pragati K. Prasad et al.

Abbreviations. Acetonitrile (ACN), N,N'-Dicyclohexylcarbodiimide (DCC), Dichloromethane (DCM), N,N'-Diisopropylethylamine (DIPEA), 1,2-Dimethylethylenediamine (DMEDA), N,N'-Dimethylformamide (DMF), Dimethyl sulfoxide (DMSO), 1-Ethyl-3-(3-dimethylaminopropyl) carbodiimide **(**EDC), 1-[Bis(dimethylamino)methylene]-1H-1,2,3-triazolo[4,5-b]pyridinium3-oxide hexafluorophosphate (HATU), Formalin-fixed paraffin-embedded (FFPE), Nitrilotriacetic acid (NTA), Paraformaldehyde (PFA), Polyacrylamide gel electrophoresis (PAGE), Phosphate buffer saline (PBS), Reverse phase high-performance liquid chromatography (RP-HPLC), Sodium dodecyl sulfate (SDS), 2-(1H-6-chlorobenzotriazole-1-yl)-1,1,3,3-tetramethyluroniumtetrafluoroborate (TCTU), Trifluorocetic acid (TFA), Thin layer chromatograpy (TLC).

Synthetic Procedures:

Synthesis of Anisamide Derivative:

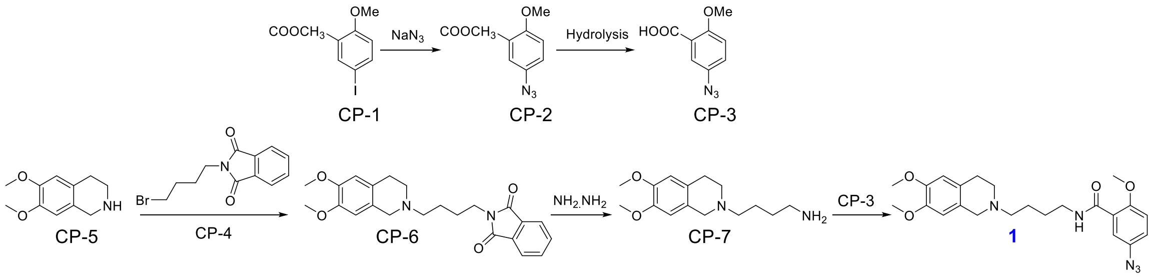

Scheme S1. Synthesis of anisamide derivative.

Synthesis of CP-2. To a stirred solution of CP-1 (1.0 g, 3.42 mmol) in 20 mL of dry DMF at room temperature, was added copper (I) iodide (650 mg, 3.42 mmol), DMEDA (45 mg, 0.51 mmol) and sodium ascorbate (34 mg, 0.17 mmol). This was followed by addition of sodium azide (450 mg, 6.84 mmol), the reaction mixture was allowed to stir at an elevated temperature of 80 °C. On completion of the reaction (as monitored by TLC), DMF was evaporated under reduced pressure while the residue was re-suspended in H_2O/EtOAc (1:1). The aqueous layer was extracted with ethyl acetate (3 × 25 mL). The organic layers were combined, dried over anhydrous sodium sulphate and evaporated under reduced

pressure. The resulting crude mixture was purified by flash column chromatography (EtOAc/hexane, 10:90) to yield an off-white solid (490 mg, 70%). ^{1}H NMR (400 MHz, $CDCl_3$): δ 3.89 (s, 3H), 3.90 (s, 3H), 6.97 (d, J = 9.0 Hz, 1H), 7.11 (dd, J = 9.0, 2.9 Hz, 1H), 7.47 (d, J = 2.9 Hz, 1H). ^{13}C NMR (100 MHz, $CDCl_3$): δ 52.2, 56.4, 113.6, 121.1, 121.9, 123.8, 132.2, 156.4, 165.7. ESI-MS (m/z): calcd. For $[M+H]^+$ 208.07; found: 208.09.

Synthesis of CP-3. To a stirred solution of CP-2 (250 mg, 1.2 mmol) in 5 mL of THF: MeOH: H_2O (3:1:1) at room temperature, was added lithium hydroxide monohydrate (255 mg, 6.0 mmol). The reaction mixture was stirred at room temperature for 4 h. On completion of the reaction (as monitored by TLC), the solvents were evaporated under reduced pressure while the resulting crude mixture was purified by flash column chromatography (MeOH/DCM, 5:95) to yield a colorless powder (200 mg, 86%).^{1}H NMR (500 MHz, DMSO-d_6): δ 3.81 (s, 3H), 7.17 (d, J = 8.9 Hz, 1H), 7.26 (dd, J = 8.9, 3.0 Hz, 1H), 7.31 (d, J = 3.0 Hz, 1H), 12.90 (br. s., 1H); ^{13}C NMR (125 MHz, DMSO-d_6): δ 56.2, 114.3, 120.8, 122.7, 123.4, 131.2, 155.4, 166.4. ESI-MS (m/z): calcd. For $[M+H]^+$ 194.06; found: 194.07.

Synthesis of CP-6. To a stirred solution of CP-5 (1.0 g, 4.36 mmol) in 70 mL of anhydrous ethanol at room temperature, was added 2-(4-bromobutyl)isoindoline-1,3-dione (1.35g, 4.80 mmol), followed by addition of K_2CO_3 (660 mg, 4.80 mmol). The reaction mixture was refluxed at 80 °C overnight. After completion of the reaction, the mixture was filtered and the filtrate was evaporated under reduced pressure to afford the crude product. The crude mixture was purified by flash column chromatography (MeOH/DCM, 3:97) to yield an off-white solid (1.5 g, 87%). ^{1}H NMR (400 MHz, $CDCl_3$): δ 1.52-1.63 (m, 2H), 1.66-1.76 (m, 2H), 2.47 (t, J = 7.3 Hz, 2H), 2.62 (t, J = 5.6 Hz, 2H), 2.73 (t, J = 5.6 Hz, 2H), 3.47 (s, 2H), 3.67 (t, J = 6.9 Hz, 2H), 3.76 (br. s, 6H), 6.44 (s, 1H), 6.51 (s, 1H), 7.63 (dd, J = 5.2, 3.2 Hz, 2H), 7.70-7.88 (m, 2H). ^{13}C NMR (100 MHz, DMSO-d_6): δ 23.9, 25.9, 28.3, 37.4, 50.7, 55.1, 55.4, 55.4, 57.1, 109.9, 111.7, 122.9, 125.9, 126.6, 131.6, 134.3, 146.8, 147.1, 167.9. ESI-MS (m/z): calcd. For $[M+H]^+$ 395.20; found: 395.19.

Synthesis of CP-7. To a stirred a solution of CP-6 (473 mg, 1.2 mmol) in 20 mL of anhydrous ethanol was added hydrazine monohydrate (750 mg, 15 mmol) and the solution was refluxed for 1 h at 80 °C. The reaction mixture was cooled and treated with an

additional 20 mL of ethanol and concentrated HCl (1.3 mL). The reaction mixture was then refluxed for another 4 h and left overnight in a refrigerator. It resulted in formation of fine crystals, the solution was filtered. The residue was dissolved in n-hexane (20 mL) and NH_4OH (15 mL). The solution was extracted with $CHCl_3$ (3 × 15 mL), the organic layer was dried over anhydrous K_2CO_3, and the solvents were evaporated to give CP-7 (colourless powder, 250 mg, 79%) which was used without further purification. ^{1}H NMR (400 MHz, DMSO-d_6): δ 1.61-1.71 (m, 2H), 1.84-1.94 (m, 2H), 2.81 (t, J = 7.2 Hz, 2H), 3.16 (t, J = 7.2 Hz, 2H), 3.72 (s, 3H), 3.73 (s, 3H), 6.77 (s, 1H), 6.80 (s, 1H). ESI-MS (m/z): calcd. For $[M+H]^+$ 265.19; found: 265.20.

Synthesis of 1. To a stirred solution of CP-4 (25 mg, 0.129 mmol) in 5 mL of DCM:THF (1:1) mixture, under argon atmosphere, was added DCC (40 mg, 0.1932 mmol), followed by the addition of Et_3N (15.6 mg, 0.1546 mmol). After 10 min., CP-7 (40 mg, 0.1546 mmol) was added. The reaction mixture was stirred under argon at room temperature for 24 h. After completion of the reaction, the solvent was evaporated under reduced pressure and the crude was purified by RP-HPLC to yield pure compound **1** (colourless oil, 42 mg, 74%). ^{1}H NMR (500 MHz, ACN-d_3): δ 1.67 (quin, J = 7.0 Hz, 2H), 1.82-1.90 (m, 2H), 2.88- 3.00 (m, 1H), 3.10-3.16 (m, 1H), 3.17-3.28 (m, 4H), 3.43 (q, J = 6.5 Hz, 2H), 3.62-3.69 (m, 1H), 3.76 (s, 3H), 3.78 (s, 3H), 3.94 (s, 3H), 4.06 (dd, J = 14.9, 4.7 Hz, 1H), 4.43 (d, J = 14.9 Hz, 3H), 6.67 (s, 1H), 6.76 (s, 1H), 7.10- 7.14 (m, 1H), 7.15-7.19 (m, 1H), 7.64 (d, J = 2.8 Hz, 1H), 8.09 (br. s., 1H). ^{13}C NMR (125 MHz, ACN-d_3): δ 22.0, 25.3, 27.5, 39.3, 50.5, 53.1, 56.1, 56.4, 56.5, 57.3, 110.6, 112.5, 114.7, 120.5, 122.4, 124.1, 133.8, 149.4, 150.1, 156.1, 165.5. ESI-MS (m/z): calcd. For $[M+H]^+$ 440.23; found: 440.23.

Synthesis of Glutamate Urea:

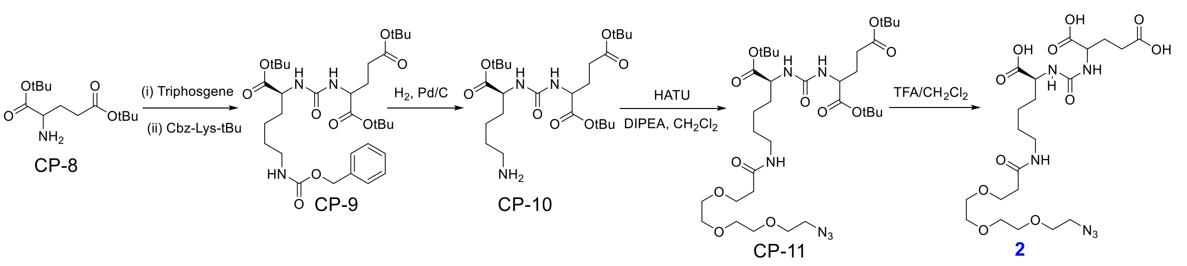

Scheme S2. Synthesis of Glutamate urea derivative.

Synthesis of CP-9. Triphosgene (400 mg, 1.35 mmol) was added dropwise to a stirred solution of CP-8 (1.2 g, 4.05 mmol) in dry DCM under argon at 0 °C. After addition of DIPEA (1.5 g, 11.62 mmol), the mixture was stirred for 4 h at 0 °C. After 4 h, the reaction was brought to room temperature and lysine derivative, *tert*-butyl N^6-((benzyloxy)carbonyl)-*L*-lysinate hydrochloride (1.0 g, 2.70 mmol) was added. The reaction mixture was further stirred for 1 h at room temperature, followed by evaporation of the solvent at reduced temperature and purification by column chromatography to yield CP-9 (580 mg, 69%) as a colourless oil. ^{1}H NMR (500 MHz, $CDCl_3$): δ 1.23- 1.36 (m, 2H), 1.45 (s, 9H), 1.47 (s, 9H), 1.48 (s, 9H), 1.50-1.58 (m, 2H), 1.66-1.76 (m, 1H), 1.78-1.86 (m, 1H), 1.86-1.94 (m, 1H), 2.05 (dq, J = 13.8, 6.7 Hz, 1H), 2.35-2.41 (m, 2H), 3.18 (t, J = 6.7 Hz, 2H), 4.21-4.33 (m, 2H), 5.11 (s, 2H), 7.29-7.38 (m, 5H). ^{13}C NMR (125 MHz, $CDCl_3$): δ 22.2, 27.8, 27.8, 27.8, 28.1, 29.1, 31.4, 32.4, 40.5, 52.6, 53.1, 66.2, 80.2, 81.3, 81.9, 127.7, 127.8, 128.2, 136.6, 156.5, 157.1, 172.1, 172.5, 172.8. ESI-MS (m/z): calcd. For $[M+H]^+$ 622.37; found: 622.37.

Synthesis of CP-10. Compound CP-9 (460 mg, 0.74 mmol) was dissolved in 30 mL MeOH and purged with argon. Next, 10% Pd/C (40 mg, 0.037 mmol) was added and the reaction was stirred overnight under H_2 (836 Torr). The mixture was filtered over celite and the solvent was removed under reduced pressure to yield CP-10 as a yellow viscous oil (322 mg, 90%). ^{1}H NMR (400 MHz, MeOH-d_4): δ 1.19-1.35 (m, 2H), 1.45 (s, 9H), 1.47 (s, 9H), 1.48 (s, 9H), 1.57-1.69 (m, 2H), 1.75-1.86 (m, 2H), 2.02-2.10 (m, 1H), 2.27-2.38 (m, 2H), 2.85 (t, J = 7.0 Hz, 1H), 3.35 (br. s, 2H), 4.14- 4.22 (m, 2H). ^{13}C NMR (100 MHz, MeOH-d_4): δ 23.8, 28.5, 28.5, 29.1, 32.6, 33.2, 54.2, 54.7, 61.6, 81.8, 82.7, 82.8, 160.0, 173.5, 173.8, 173.8. ESI-MS (m/z): calcd. For $[M+H]^+$ 488.33; found: 488.34.

Synthesis of CP-11. 3-(2-(2-(2-azidoethoxy)ethoxy)ethoxy)propanoic acid (30 mg, 0.1232 mmol) was dissolved in dry DCM (5 mL) and stirred in a reaction vial at room temperature. Then, HATU (78 mg, 0.206 mmol) and DIPEA (27 mg, 0.206 mmol) were sequentially added. After stirring the reaction mixture for 10 minutes, CP-10 (50 mg, 0.103 mmol) dissolved in DCM was added dropwise to the reaction mixture via a syringe. The reaction was continued for 18h (until completion), followed by evaporation of the solvent and purification of the resulting crude by RP-HPLC to yield pure compound CP-11 (56 mg,

76%). ^{1}H NMR (500 MHz, ACN-d_3): δ 1.31-1.37 (m, 2H), 1.42 (s, 9H), 1.43 (s, 9H), 1.43 (s, 9H), 1.44-1.50 (m, 2H), 1.53-1.63 (m, 1H), 1.66-1.80 (m, 2H), 1.96-2.02 (m, 1H), 2.18-2.32 (m, 2H), 2.37 (t, J = 6.1 Hz, 2H), 3.12- 3.18 (m, 2H), 3.37 (t, J = 4.9 Hz, 2H), 3.56 (d, J = 2.3 Hz, 4H), 3.59 (br. s., 4H), 3.61-3.64 (m, 2H), 3.66 (t, J = 5.9 Hz, 2H), 4.05 (br. s, 1H), 4.14 (br. s, 1H), 5.44 (br. s, 1H), 5.50 (br. s, 1H), 6.77 (br. s, 1H). ^{13}C NMR (125 MHz, ACN-d_3): δ 23.4, 28.3, 28.3, 28.4, 28.9, 29.8, 32.2, 32.6, 37.4, 39.6, 51.6, 54.0, 54.7, 67.9, 70.6, 71.0, 71.1, 71.2, 71.2, 81.0, 81.9, 82.3, 158.5, 172.6, 173.0, 173.1, 173.4. ESI-MS (m/z): calcd. For $[M+H]^+$ 717.44; found: 717.44.

Synthesis of 2. TFA (1 mL) was added to a cooled solution (0 °C) of CP-11 (50 mg, 0.061 mmol) in DCM (3 mL). The reaction mixture was warmed up to room temperature and stirring was continued for another 3 h. After the reaction was complete, DCM and TFA were evaporated. The traces of TFA were removed by co-evaporation with DCM. The crude compound was dissolved in 3 mL ACN/H_2O (1:1), frozen with liquid nitrogen and lyophilized under high vacuum to afford a colourless viscous oil as product (30 mg, 90%). ^{1}H NMR (500 MHz, DMSO-d_6): δ 1.22-1.30 (m, 2H), 1.33-1.41 (m, 2H), 1.45-1.55 (m, 1H), 1.58-1.67 (m, 1H), 1.67-1.77 (m, 1H), 1.91 (td, J_1 = 14.1 Hz, J_2 = 6.5 Hz, 1H), 2.15-2.32 (m, 4H), 3.00 (dt, J_1 = 5.4 Hz, J_2 = 16.0 Hz, 2H), 3.39 (t, J = 4.8 Hz, 2H), 3.45-3.62 (m, 12H), 4.00- 4.06 (m, 1H), 4.06-4.12 (m, 1H), 6.28 (d, J = 8.1 Hz, 1H), 6.32 (d, J = 8.3 Hz, 1H), 7.80 (t, J = 5.4 Hz, 1H). ^{13}C NMR (125 MHz, DMSO-d_6): δ 22.6, 27.5, 28.8, 29.9, 31.8, 36.1, 38.3, 50.0, 51.6, 52.2, 66.9, 69.2, 69.5, 69.7, 69.7, 69.8, 157.3, 169.8, 173.7, 174.1, 174.5. ESI-MS (m/z): calcd. For $[M+H]^+$ 549.25; found: 549.25.

Synthesis of Folate Derivative:

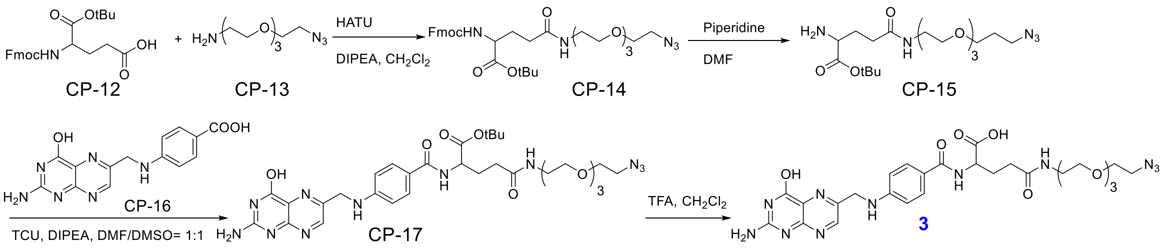

Scheme S3. Synthesis of folate derivative.

Synthesis of CP-14. To a solution of Fmoc-Glu-OtBu (CP-12) (212 mg, 0.5 mmol) in dry CH_2Cl_2 (10 mL), HATU (380.0 mg, 1.0 mmol) and DIPEA (130 mg, 1.0 mmol) were added. After 20 min stirring at room temperature, 11-azido-3,6,9-trioxaundecan-1-amine (CP-13) (145.0 mg, 0.65 mmol) was added and the mixture was stirred at room temperature overnight. After completion of the reaction (monitored by TLC), the reaction mixture was concentrated under reduced pressure and purified by flash column chromatography (MeOH:CH_2Cl_2, 5:95). The product CP-14 was obtained as a yellowish viscous oil (280 mg, 90%). ^{1}H NMR (300 MHz, $CDCl_3$): δ 1.47 (s, 9H), 1.60 (m, 4H), 2.25 (m, 2H), 3.32-3.42 (m, 2H), 3.42-3.50 (m, 2H), 3.50-3.59 (m, 2H), 3.59-3.71 (m, 10H), 4.18-4.28 (m, 2H), 4.33-4.46 (m, 2H), 5.68 (d, J = 7.7 Hz, 1H), 7.29-7.36 (m, 2H), 7.36-7.46 (m, 2H), 7.62 (d, J = 6.4 Hz, 2H), 7.78 (d, J = 7.5 Hz, 2H).^{13}C NMR (75 MHz, $CDCl_3$): δ 27.9, 28.6, 32.4, 39.2, 47.1, 50.6, 54.1, 66.9, 69.7, 69.9, 70.1, 70.4, 70.5, 70.6, 82.3, 119.9, 125.1, 127.0, 127.6, 141.2, 143.7, 143.9, 156.2, 171.1, 172.0. ESI-MS (m/z): calcd. For $[M+H]^+$ 626.73, found: 626.67.

Synthesis of CP-15. Compound CP-14 (250 mg, 0.4 mmol) was dissolved in dry DMF (6.0 mL) and piperidine (6.9 mg, 0.08 mmol, 8 µL) was added. The reaction mixture stirred for 2h at room temperature. After completion of the reaction, the reaction mixture was concentrated under reduced pressure. The crude mixture was purified by flash column chromatography (MeOH:CH_2Cl_2, 10:90). The amine product CP-15 was obtained as a colourless oil (120 mg, 75%). ^{1}H NMR (300 MHz, $CDCl_3$): δ 1.45 (s, 9H), 1.70-1.73 (m, 2H), 2.00-2.20 (m, 2H), 2.27-2.38 (m, 2H), 3.25-3.50 (m, 5H), 3.51-3.58 (m, 2H), 3.58-3.75 (m, 10H), 5.30 (d, J = 4.2 Hz, 2H), 6.53 (br. s, 1H). ESI-MS (m/z): calcd. For $[M+H]^+$ 404.49, found: 404.52.

Synthesis of CP-17. To a suspension of pteroic acid (CP-16) (68 mg, 0.218 mmol) in a mixture of dry DMF/DMSO (1:1, 3.0 mL), TCTU (112 mg, 0.273 mmol) and DIPEA (35.4 mg, 0.273 mmol) were added. After 30 minutes stirring at room temperature, a solution of CP-15 (110 mg, 0.273 mmol) in dry DMF/DMSO (1:1, 4 mL) was added to the reaction vessel, and the mixture was stirred at room temperature for 12 h. After completion of the reaction (monitored by TLC), the reaction mixture was diluted with water (20 mL) and extracted with EtOAc (3 × 20 mL). The organic layers were combined and washed with

saturated brine (2 × 20 mL). The crude product was purified by flash column chromatography (MeOH:CH_2Cl_2, 10:90). The pure product was obtained as a dark yellow solid (98 mg, 65%). ^{1}H NMR (300 MHz, DMSO-d_6): δ 1.23 (t, J = 7.3 Hz, 2H), 1.38 (s, 9H) 1.75- 2.06 (m, 2H) 2.06-2.27 (m, 2H) 3.17-3.67 (m, 16H), 4.10-4.27 (m, 1H) 4.48 (d, J = 5.0 Hz, 2H) 6.63 (m, J = 8.5 Hz, 2H) 6.97 (t, J = 5.6 Hz, 1H) 7.64 (m, J = 8.5 Hz, 2H) 7.93 (t, J = 5.1 Hz, 1H) 8.20 (d, J = 7.3 Hz, 1H) 8.64 (s, 1H). ESI-MS (m/z): calcd. For $[M+H]^+$ 698.76, found 698.71.

Synthesis of 3. CP-17 (69.7 mg, 0.1 mmol) was dissolved in $CHCl_3$ (2.8 mL) and TFA (0.70 mL) and stirred at room temperature for 3 h. After completion of the deprotection, the solvents were evaporated under vacuum and the solid residue was washed with cold diethyl ether (3 × 3mL). The crude compound was then dissolved in 3 mL ACN/H_2O (1:1), frozen with liquid nitrogen and lyophilized under high vacuum to afford compound **3** as a yellow fluffy powder (25 mg, 40%). ^{1}H NMR (500 MHz, DMSO-d_6): δ 1.85-1.95 (m, 1H) 1.99-2.08 (m, 1H), 2.13-2.26 (m, 2H), 3.17 (q, J = 5.7 Hz, 2H), 3.33-3.40 (m, 3H), 3.43-3.63 (m, 11H), 4.22-4.31 (m, 1 H), 4.50 (s, 2H), 6.64 (d, J = 8.5 Hz, 2H), 7.15 (br. s., 2H), 7.65 (d, J = 8.5 Hz, 2H), 7.88 (t, J = 5.3 Hz, 1H), 8.19 (d, J = 7.3 Hz, 1H), 8.66 (s, 1H). ^{13}C NMR (125 MHz, DMSO-d_6): δ 26.5, 31.9, 38.5, 45.9, 50.0, 52.2, 69.1, 69.2, 69.5, 69.7, 69.7, 69.8, 111.0, 111.2, 121.4, 127.9, 128.9, 131.0, 148.4, 149.2, 150.7, 153.5, 159.8, 160.7, 166.2, 171.7, 173.8. ESI-MS (m/z): calcd. For $[M+H]^+$: 642.65, found 642.64.

Synthesis of Tri-Nitrilotriacetic acid (4):

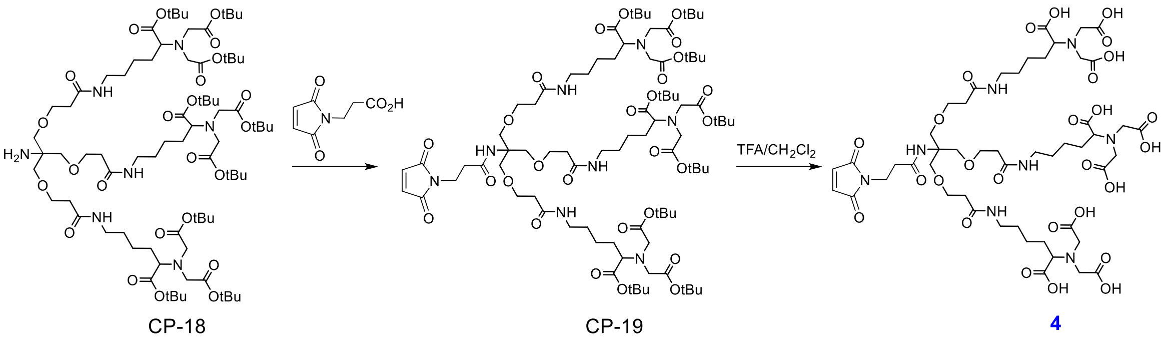

Scheme S4. Synthesis of tri-nitrilotriacetic acid.

CP-18 was synthesized according to previously reported literature from our group.[1]

Synthesis of CP-19. EDC (22 mg, 0.118 mmol) and HOBt (7.8 mg, 0.058 mmol) were added to a stirred solution of 3-maleimidopropionic acid (20 mg, 0.118 mmol) in 10 mL dry THF under argon at 0 °C. Then triethylamine (16 µL, 0.118 mmol) was added and pH of the resulting reaction mixture was adjusted to 7. After 15 min, CP-18 (140 mg, 0.09 mmol) was added and the reaction mixture was left to stir for 24 hours under argon at room temperature. After completion of the reaction, THF was removed under vacuum and the residue was purified by RP-HPLC to yield the product (95 mg, 61%). ^{1}H NMR (400 MHz, DMSO-d_6): δ 1.12-1.43 (m, 93H), 1.51 (m, 6H), 2.26 (t, J = 6.35 Hz, 6H), 2.35 (t, J = 7.48 Hz, 2H), 3.00 (m, 6H), 3.22 (t, J = 7.41 Hz, 3H), 3.34 (d, J =17.13, 6H), 3.42 (d, J =17.17, 6H), 3.45-3.56 (m, 14H), 6.25 (br s, 1H), 6.99 (s, 2H), 7.24 (s, 1H), 7.78 (t, J = 5.37 Hz, 3H); ^{13}C NMR (100 MHz, DMSO-d_6): δ 22.92, 27.77, 27.78, 28.90, 29.76, 33.95, 34.28, 35.95, 38.42, 53.28, 59.62, 64.59, 67.34, 68.20, 79.99, 80.31, 134.56, 169.85, 170.08, 170.70, 171.59, HRMS-ESI$^+$ (m/z): calcd. for [M+H]$^+$ 1726.0468; found 1726.0532; calcd. for [M+Na]$^+$ 1748.0348; found 1748.0352.

Synthesis of Compound 4. The tert-butyl ester groups were deprotected by adding TFA (1.00 mL) to a solution of CP-19 (80.0 mg, 0.046 mmol) in DCM (2 mL) at 0 °C. The reaction mixture was warmed up to the room temperature and stirring was continued for another 3.5 h. After the reaction was completed, DCM and TFA were evaporated. The traces of TFA were removed by co-evaporation with DCM. The crude compound was washed twice with cold diethyl ether, followed by dissolution in 3 mL ACN/H_2O (1:1), freezing with liquid nitrogen, and lyophilization under high vacuum to afford an off-white powder as product (48 mg, 85%). ^{1}H NMR (300 MHz, DMSO-d_6): δ 1.38 (br. s., 12H) 1.44- 1.76 (m, 6H) 2.27 (t, J = 5.7 Hz, 6H) 2.36 (t, J = 7.3 Hz, 2H) 2.93-3.09 (m, 6H) 3.40 (t, J = 7.0 Hz, 3H) 3.44- 3.65 (m, 26H) 6.98 (s, 2H) 7.25 (s, 1H) 7.81 (br. s., 3H); ^{13}C NMR (100 MHz, D_2O): δ 23.01, 26.73, 28.02, 34.21, 34.90, 36.13, 38.79, 54.34, 60.12, 66.97, 67.48, 68.49, 134.44, 169.94, 171.37, 172.54, 172.99, 173.95; HRMS-ESI$^+$ (m/z): calcd. for [M+H]$^+$ 1221.4902; found 1221.4898.

Scheme for the synthesis of ODN-ligand conjugates

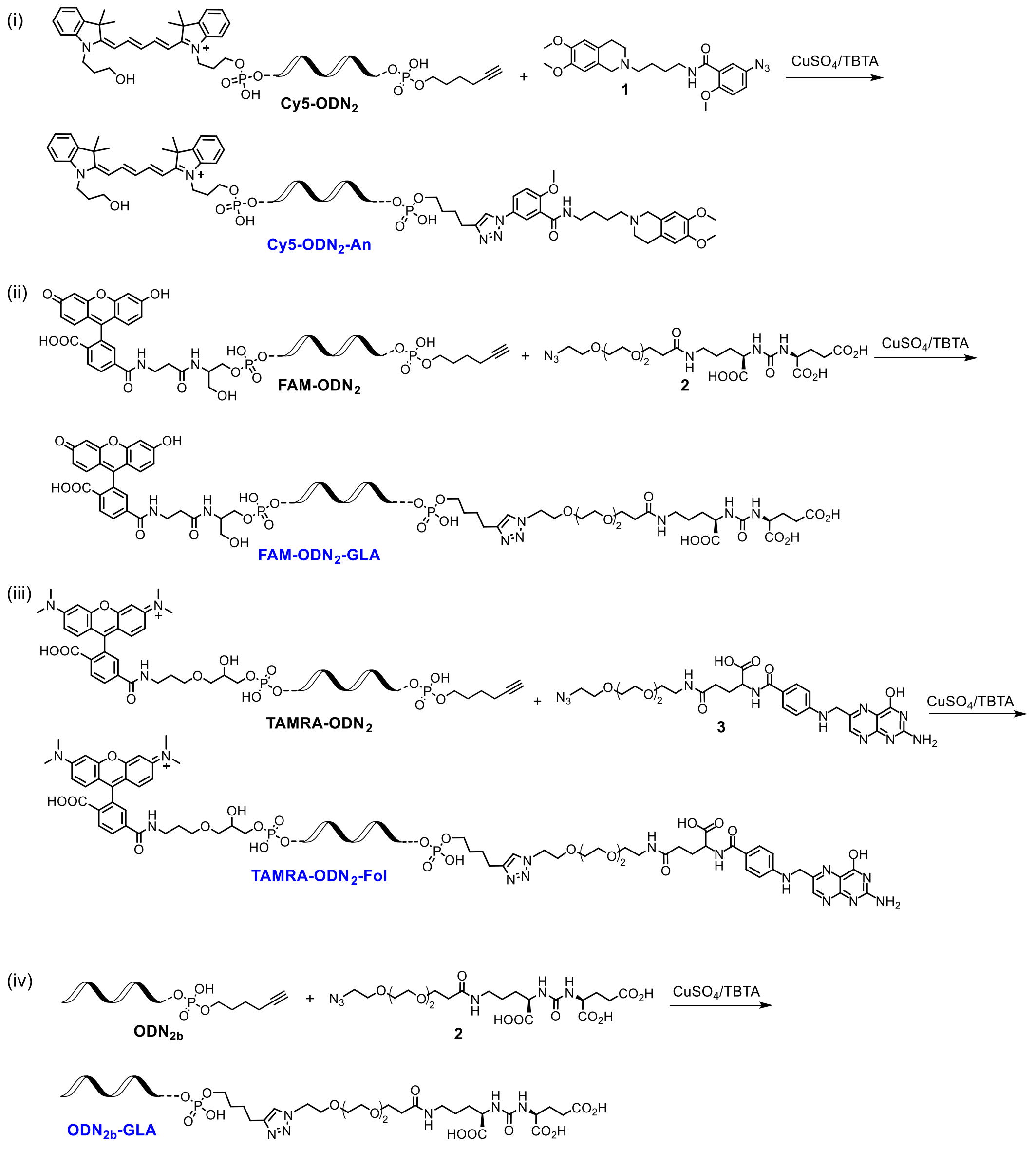

Scheme S5. Synthesis of ODN-conjugates, namely; (i) Cy5-ODN$_2$-An, (ii) FAM-ODN$_2$-GLA, (iii) TAMRA-ODN$_2$-Fol, and (iv) ODN$_{2b}$-GLA using copper catalyzed azide-alkyne coupling.

General procedure for the synthesis of ODN-ligand conjugates. 150 nmol alkyne modified oligodeoxynucleotide (Cy5-ODN$_2$, FAM-ODN$_2$, or TAMRA-ODN$_2$, or ODN$_{2b}$) was dissolved in 160 µL MQ water, followed by the addition of ascorbic acid (20 µL, 0.9 µmol), TEAA buffer (40 µL, 2M, pH = 7), and Cu-TBTA (80 µL, 0.9 µmol). The mixture

was purged with argon and finally 1.5 µmol azide modified ligand (**1, 2,** or **3**) in DMSO (200 µL) was added. The mixture was purged with argon again, sealed and placed on a shaker for 18 h. The progress of the reaction was monitored by analytical RP-HPLC. After completion of reaction, the crude was purified by RP-HPLC and characterized by ESI-MS. ESI (m/z): **Cy5-ODN$_2$-An:** calcd. $[M+Na^+]^+$ 9589.93; found 9589.66, **TAMRA-ODN$_2$-Fol:** calcd. $[M-H^+]^-$ 9839.0688; found 9839.0791, **FAM-ODN$_2$-GLA:** calcd. $[M+K^+]^+$ 9748.86; found 9748.45, **ODN$_{2b}$-GLA:** calcd. $[M-H^+]^-$ 12218.3678; found 12218.3818.

Scheme for the synthesis of tri-NTA-ODN$_1$

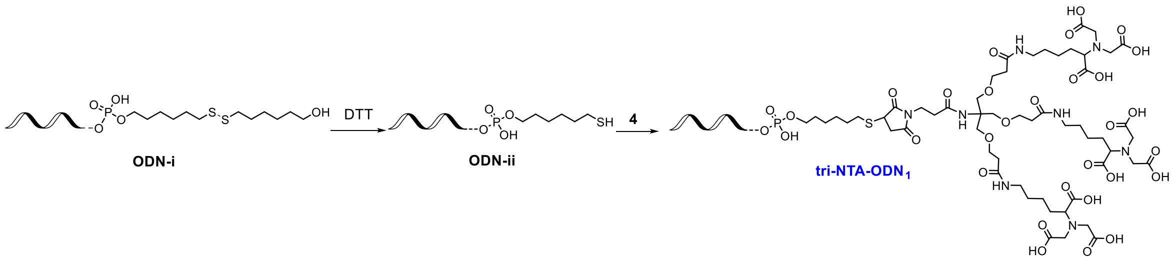

Scheme S6. Synthesis of ODN-conjugate namely tri-NTA-ODN$_1$.

Procedure for the synthesis of tri-NTA-ODN$_1$. DTT (10 µL, 1.0 M solution in water) was added to a solution of ODN-i (200 nmol) in 200 µL Tris buffer (50 mM, pH 8.3) and stirred for 1 hour. The reduced oligodeoxynucleotide (ODN-ii) was then desalted on a Sephadex™ G-25 column and dried under reduced pressure. ODN-ii was added to a solution of **4** (8 mg) in concentrated PBS ×10, pH 7. The reaction was stirred overnight, and then the product was purified using RP-HPLC. MALDI-TOF MS (m/z): ESI-MS (m/z): calcd. For $[M+H^+]^+$: 8877.14; found 8877.25.

DNA sequences

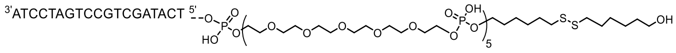

ODN-i

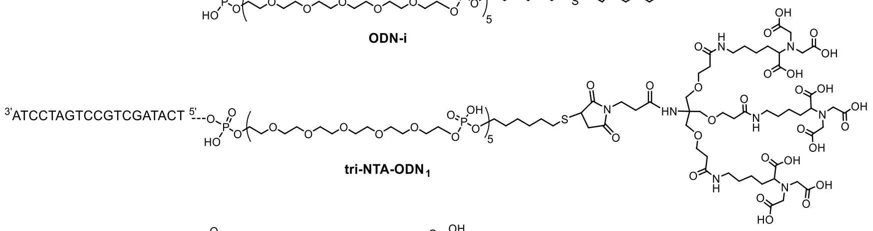

tri-NTA-ODN$_1$

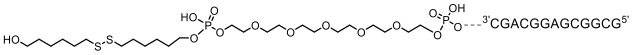

ODN$_{1a}$

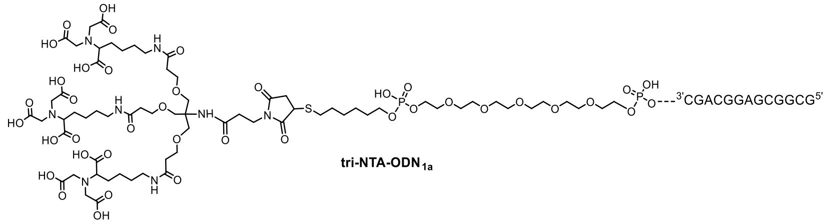

tri-NTA-ODN$_{1a}$

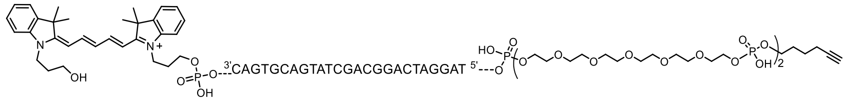

Cy5-ODN$_2$

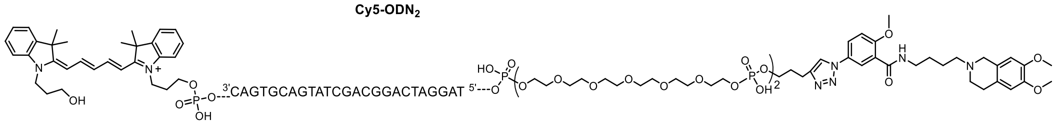

Cy5-ODN$_2$-An

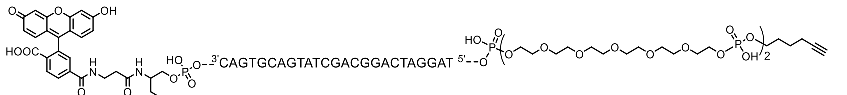

FAM-ODN$_2$

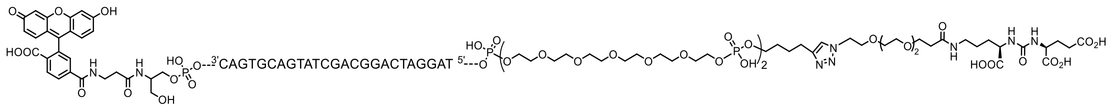

FAM-ODN$_2$-GLA

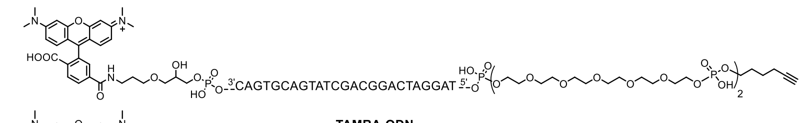

TAMRA-ODN$_2$

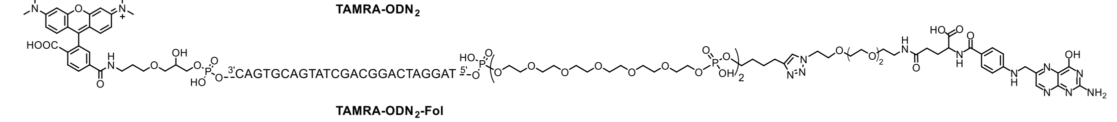

TAMRA-ODN$_2$-Fol

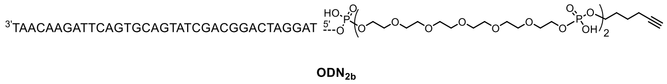

ODN$_{2b}$

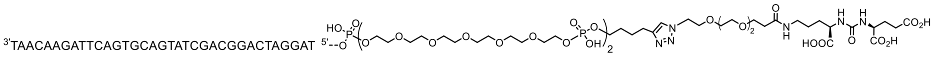

ODN$_{2b}$-GLA

3'YGCTCYCGTGYGACCYTGACYACCTYCGCCGCTCCGTCG5'   Y= Fluorescein dT

ODN_{4a}

3'YGCTCYCGTGYGACCYTGACYACCTYCGCGCACTGAATCTTGTTA 5'   Y= Fluorescein dT

ODN_{4b}

3'GCACTGAATCTTGTTA YGCTCYCGTGYGACCYTGACYACCTYCGC5'   Y= Fluorescein dT

$ODN_{4b'}$

3'GCGAAGGTAGTCAAGGTCACACGAGAGCA 5'

ODN_3

Supplementary figures

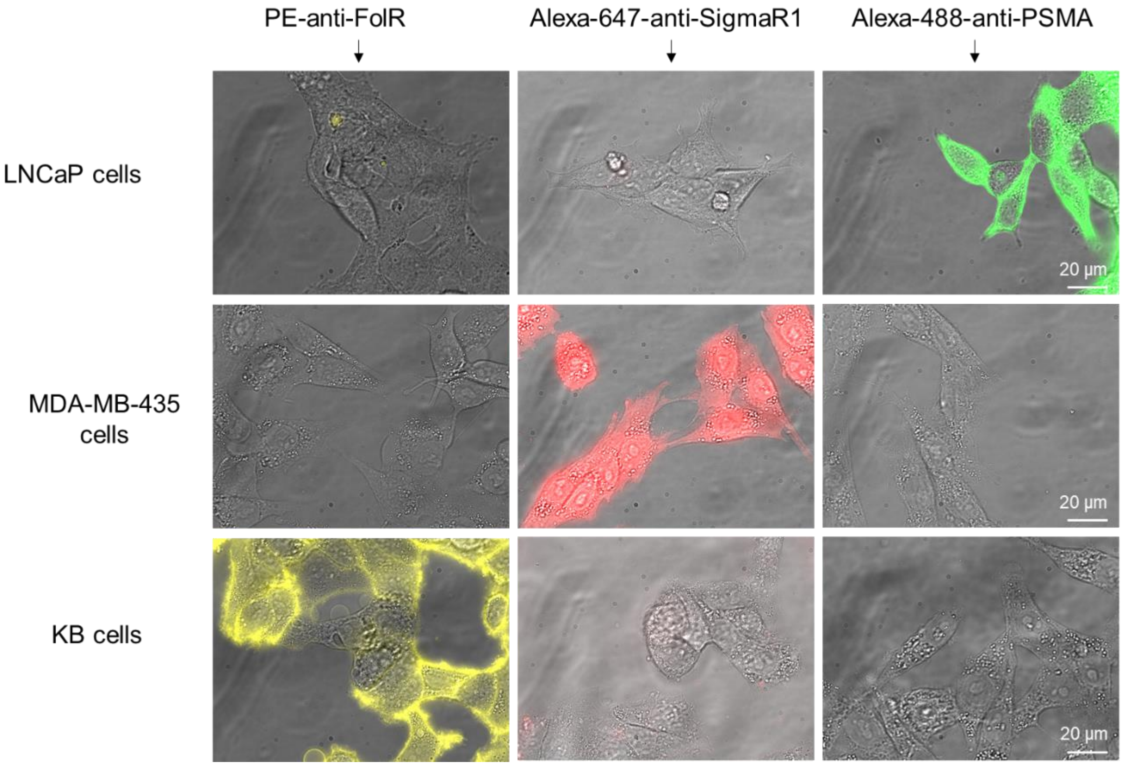

Figure S1. Immunofluorescence studies. Merged bright-field and fluorescence images of (top) LNCaP cells, (middle) MDA-MB-435 cells, and (bottom) KB cells after incubation with PE-anti-FolR, Alexafluor647-anti-sigmaR1, and Alexafluor488-anti-PSMA Abs.

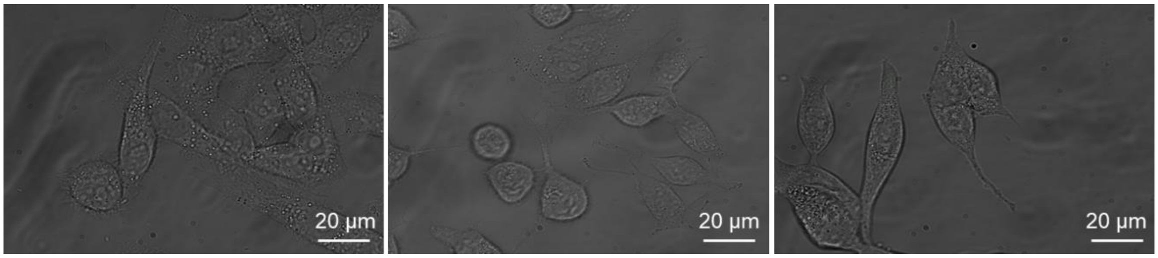

Figure S2. Merged bright-field and fluorescence images of control PBS buffer-treated KB cells (left), MDA-MB-435 cells (middle), and LNCaP cells (right).

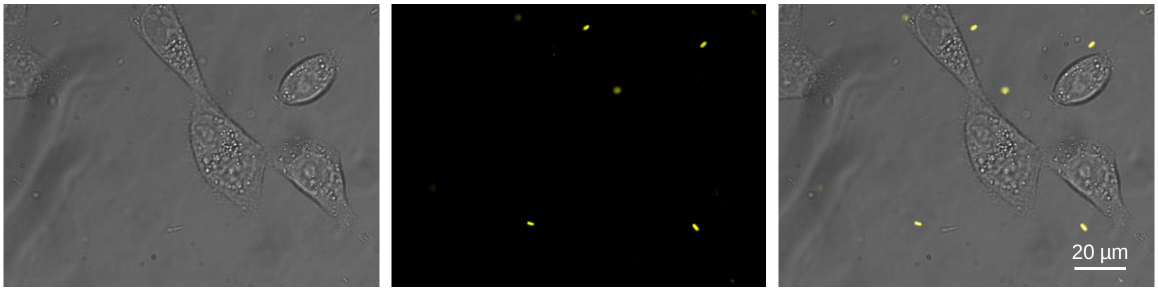

Figure S3. Displacement studies with small molecules. (Left) Bright-field, (middle) fluorescence, and (right) overlay images of KB cells after incubation with 1µM folic acid followed by incubation with B-probe 1.

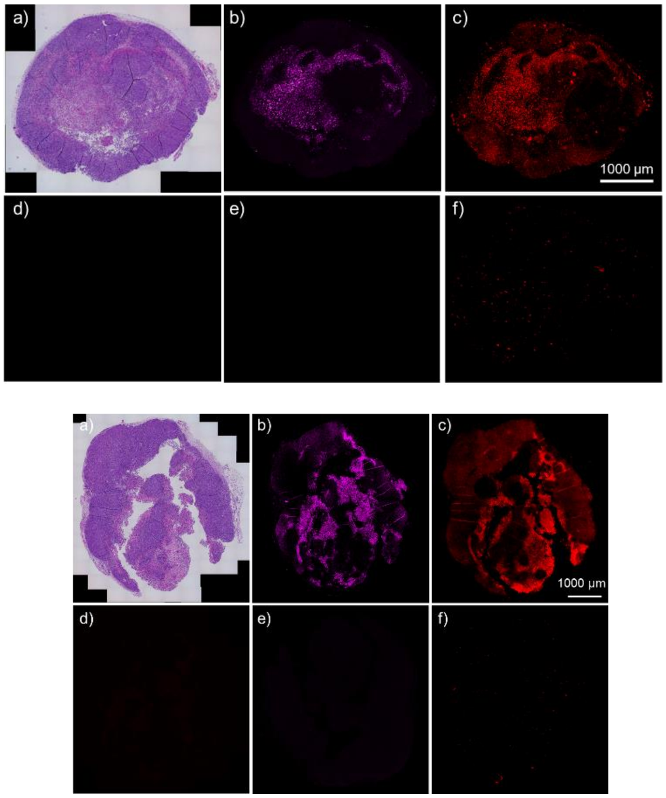

Figure S4. (Top and bottom) two representative set of images (20× magnification) showing the whole tumor section stained with **a**) hematoxylin and eosin stain, **b**) Alexafluor-647-anti-SigmaR1 Ab, **c**) B-probe 2, **d**) duplex 2, **e**) Alexafluor647-IgG2b isotype control, and **f**) bacteria modified with duplex generated from tri-NTA-ODN_1 and Cy5-ODN_2 (lacking anisamide).

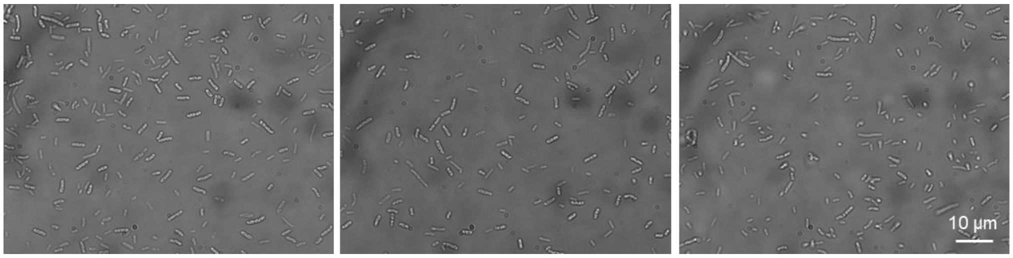

Figure S5. Merged bright-field and fluorescence images of His-bacteria upon incubation with 100 nM of (left) duplex 4, (middle) duplex 5, and (right) duplex 6 in the absence of Ni^{+2}.

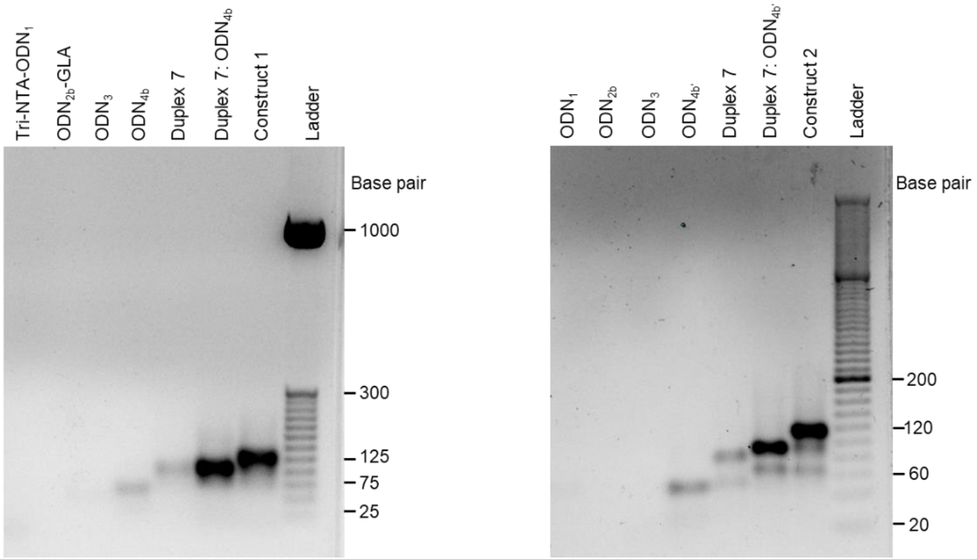

Figure S6. Native agarose gel electrophoresis stained with ethidium bromide showing the formation of the (left) construct 1, and (right) construct 2.

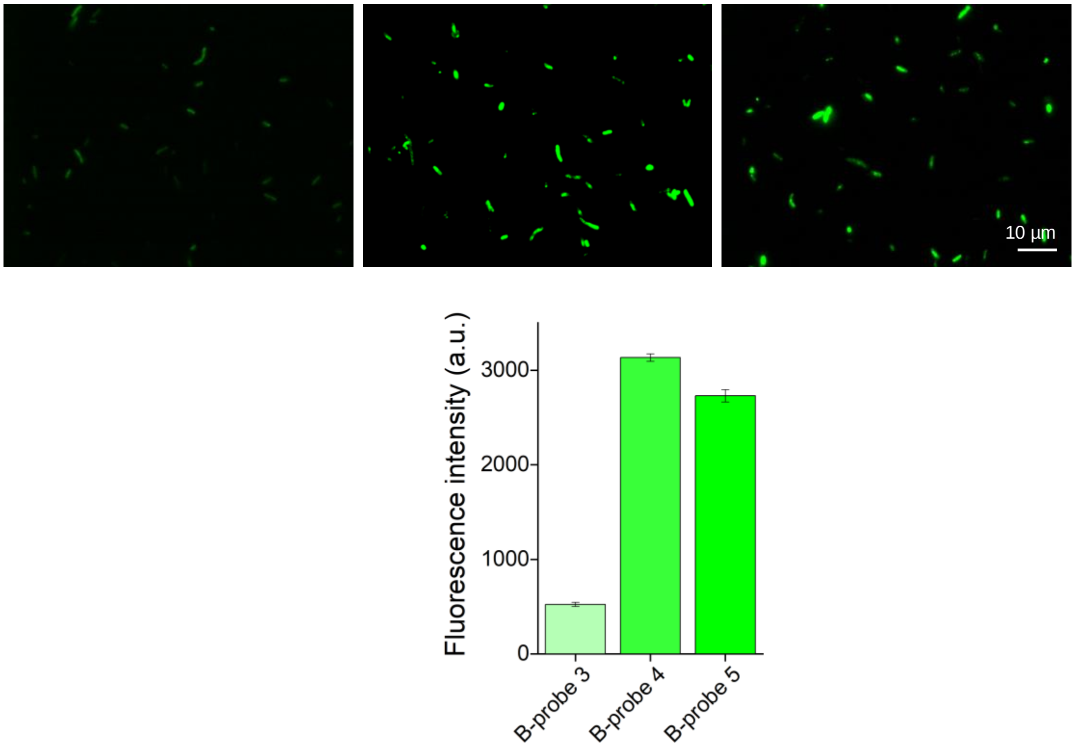

Figure S7. (Top) fluorescence images of (left) B-probe 3, (middle) B-probe 4, and (right) B-probe 5 formed upon incubation with duplex 3, construct 1 and construct 2, respectively. B-probes were prepared following incubation of bacteria with 100 nM of each duplex and 500 nM of $NiCl_2$. (Bottom) the graph bars represent the fluorescence intensities obtained from these images.

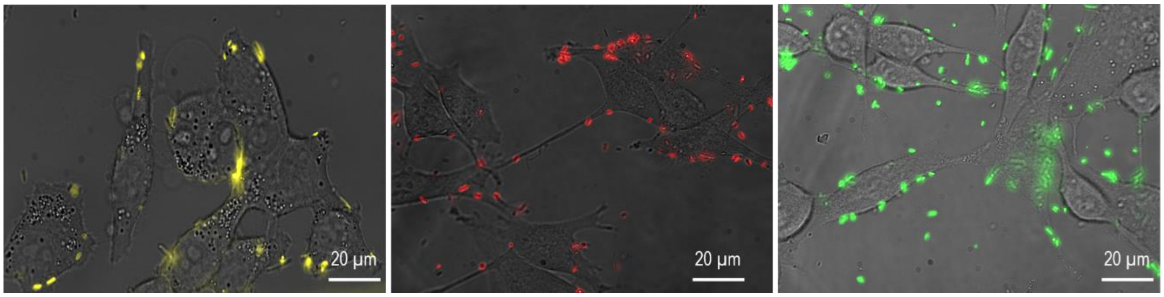

Figure S8. Merged bright-field and fluorescence images of KB cells (left), MDA-MB-435 cells (middle), and LNCaP cells (right) labelled with B-probe 1, B-probe 2, and B-probe 3, respectively, at time t=0 .

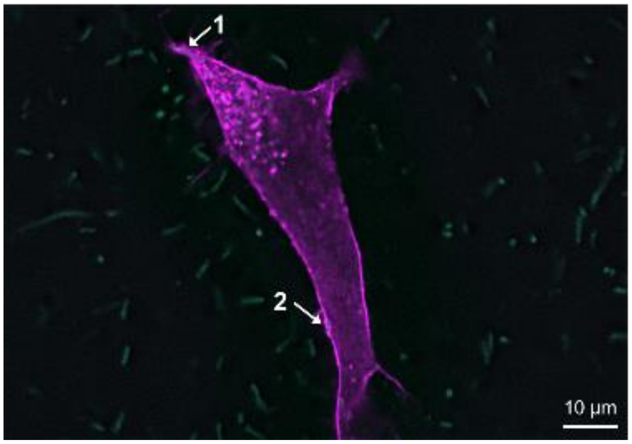

Figure S9. Live LNCaP cells expressing mCherry-CaaX incubated with B-probe 3. Arrows indicate two bacteria, which are photo-bleached.

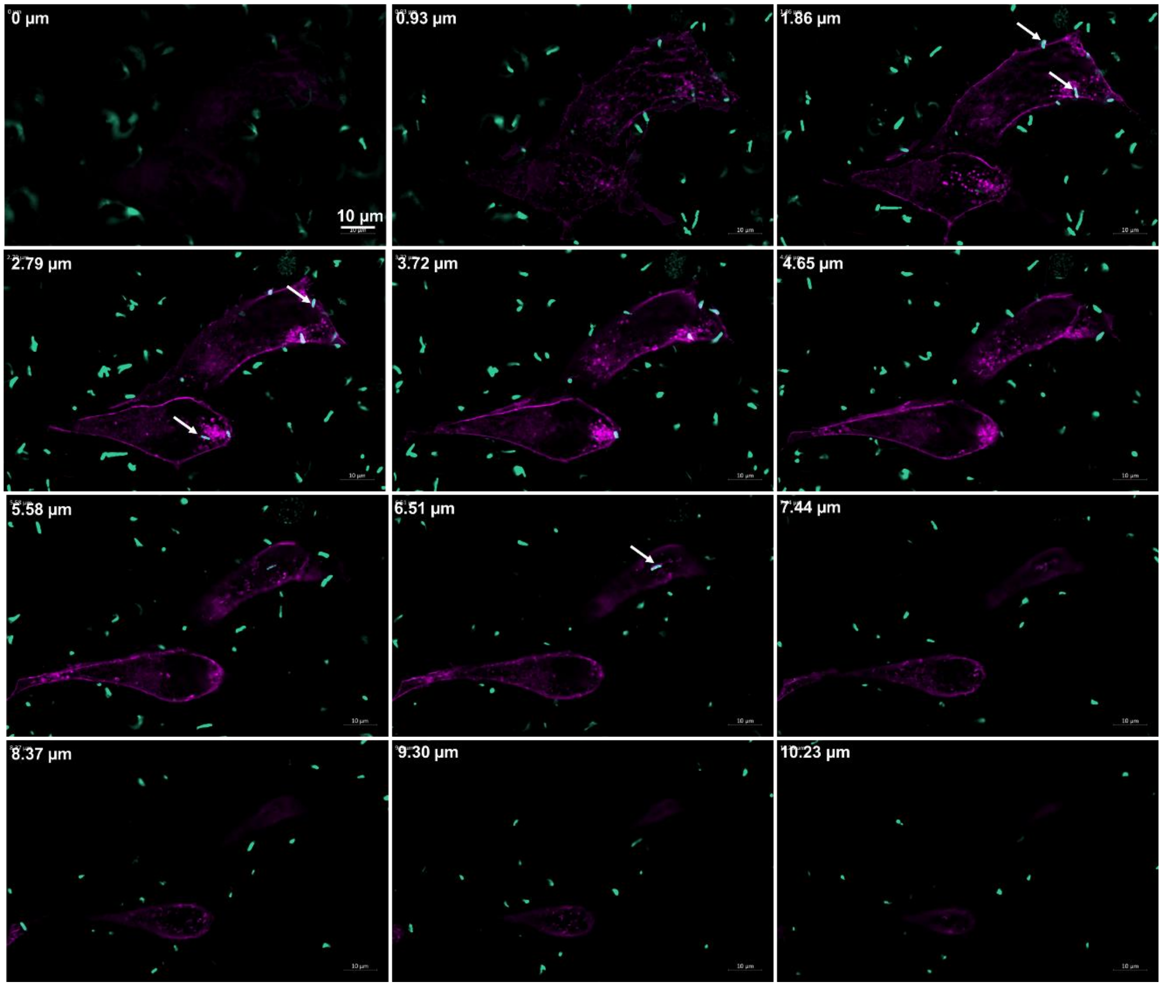

Figure S10. Z-stacks of 0.93 µm increments of selected live LNCaP cells expressing mCherry-CaaX incubated with B-probe 4.

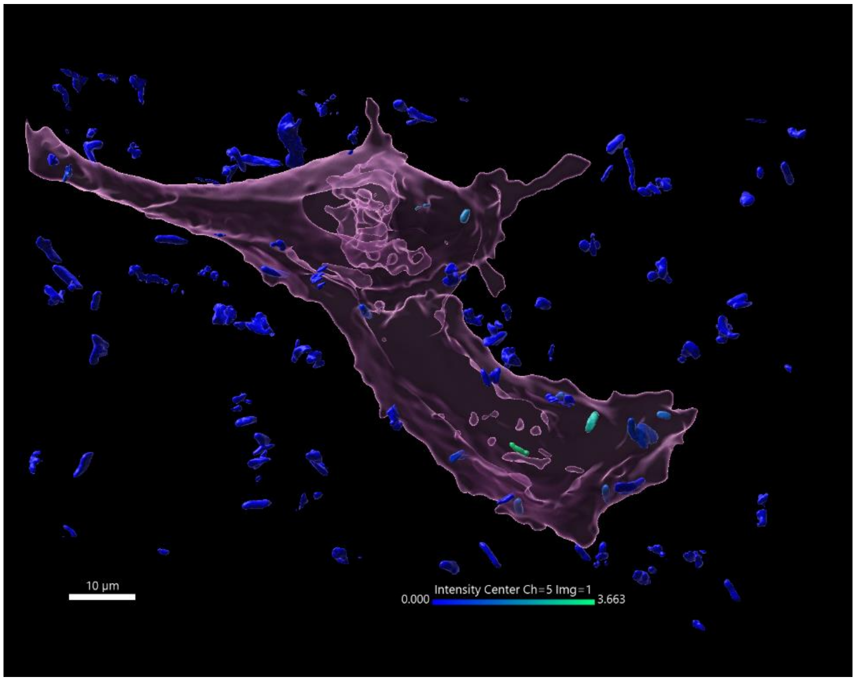

Figure S11. Inside-distances of bacteria center relative to the cell border. Bacteria located outside the cell get a zero value (deep blue), whereas bacteria located inside the cell are denoted in brighter colors (cyan/green) indicating their distance to the cell border.

The representative HPLC chromatograms of Cy5-ODN$_2$-An, FAM-ODN$_2$-GLA, TAMRA-ODN$_2$-Fol, ODN$_{2b}$-GLA, tri-NTA-ODN$_1$, ODN$_{4b}$ and ODN$_3$.

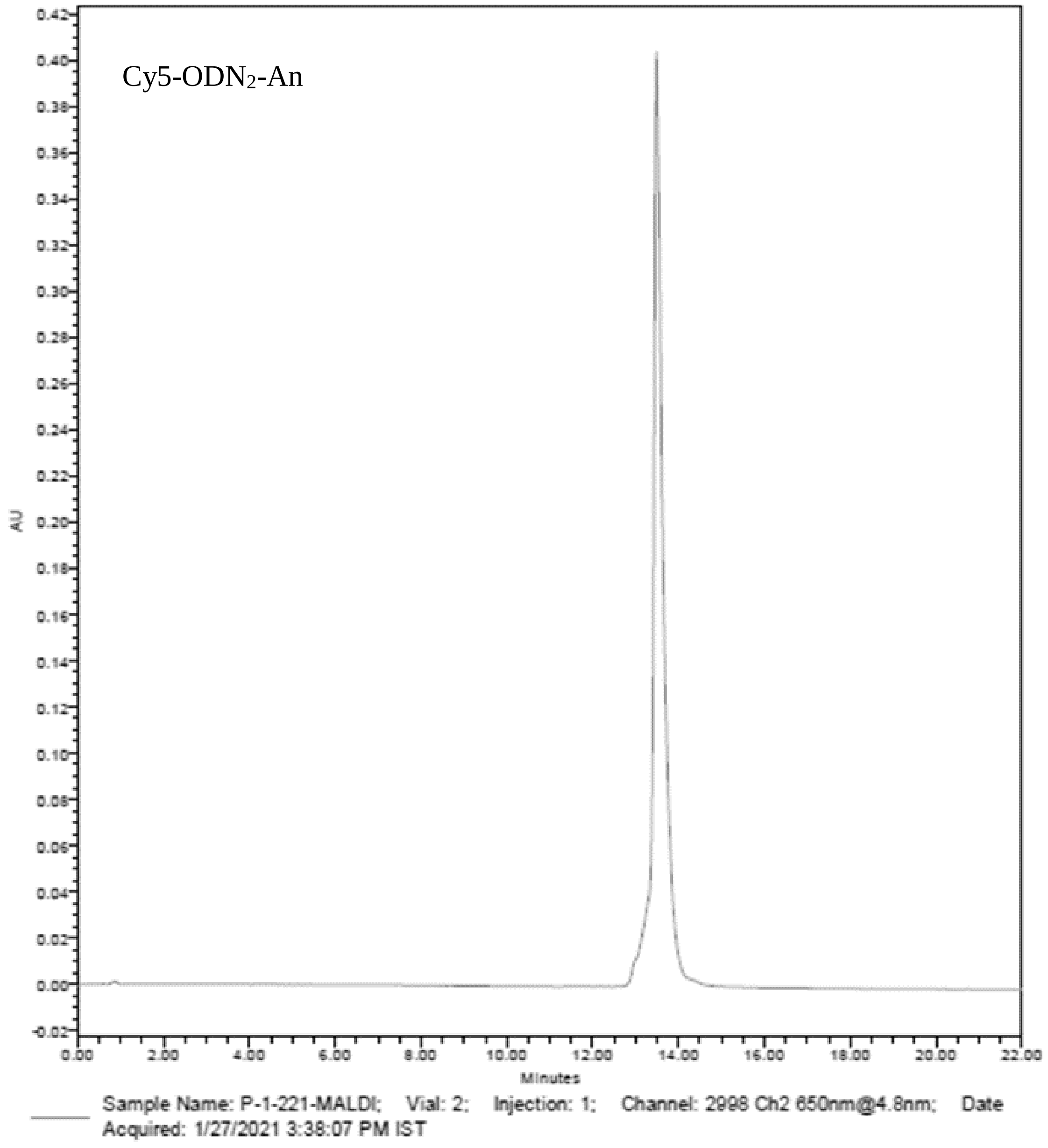

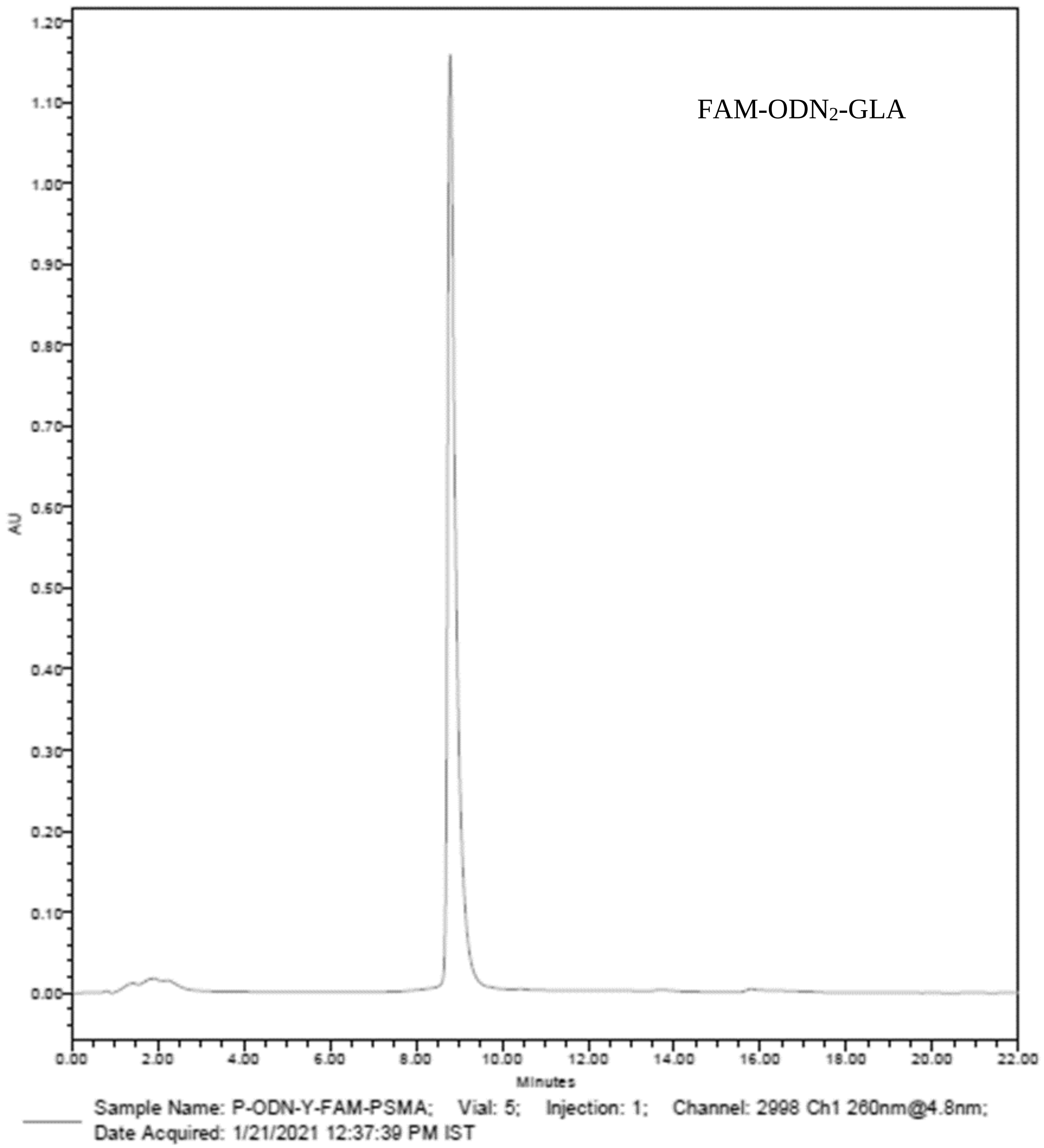
FAM-ODN2-GLA
AU
Minutes
Sample Name: P-ODN-Y-FAM-PSMA; Vial: 5; Injection: 1; Channel: 2998 Ch1 260nm@4.8nm;
Date Acquired: 1/21/2021 12:37:39 PM IST

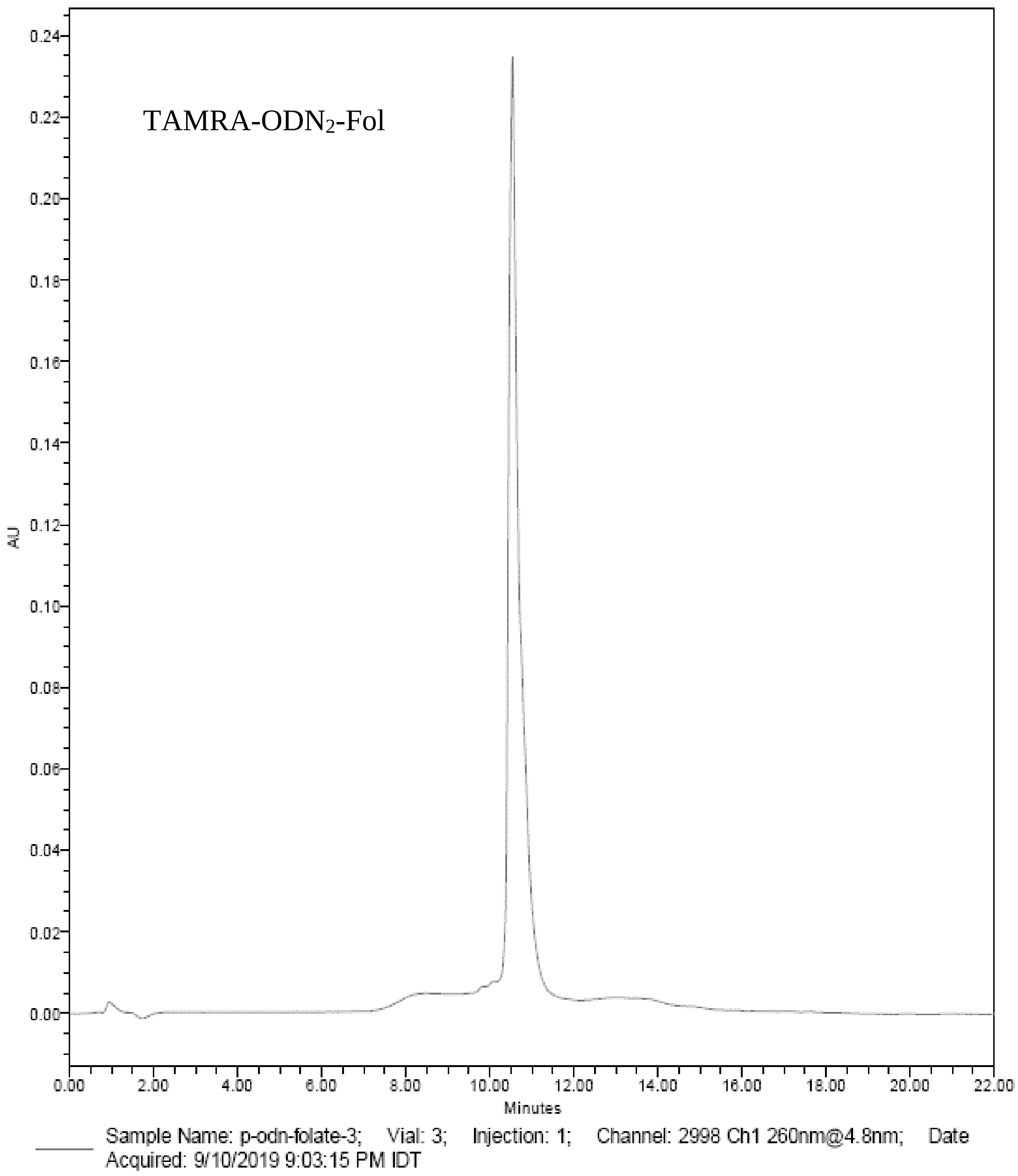
TAMRA-ODN2-Fol
AU
0.24
0.22
0.20
0.18
0.16
0.14
0.12
0.10
0.08
0.06
0.04
0.02
0.00
0.00 2.00 4.00 6.00 8.00 10.00 12.00 14.00 16.00 18.00 20.00 22.00
Minutes
Sample Name: p-odn-folate-3; Vial: 3; Injection: 1; Channel: 2998 Ch1 260nm@4.8nm; Date Acquired: 9/10/2019 9:03:15 PM IDT

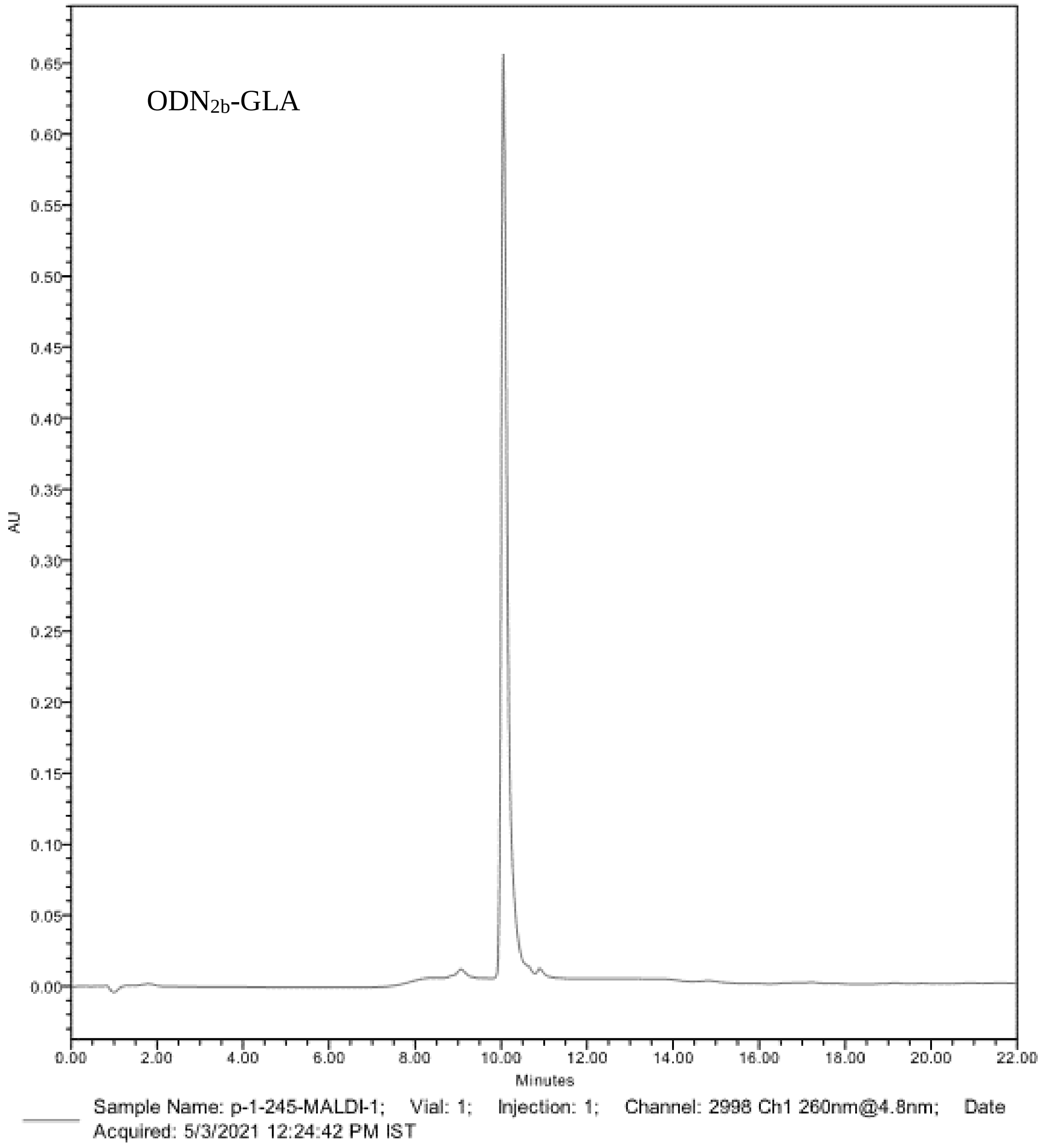

ODN2b-GLA
AU
Minutes
Sample Name: p-1-245-MALDI-1; Vial: 1; Injection: 1; Channel: 2998 Ch1 260nm@4.8nm; Date Acquired: 5/3/2021 12:24:42 PM IST

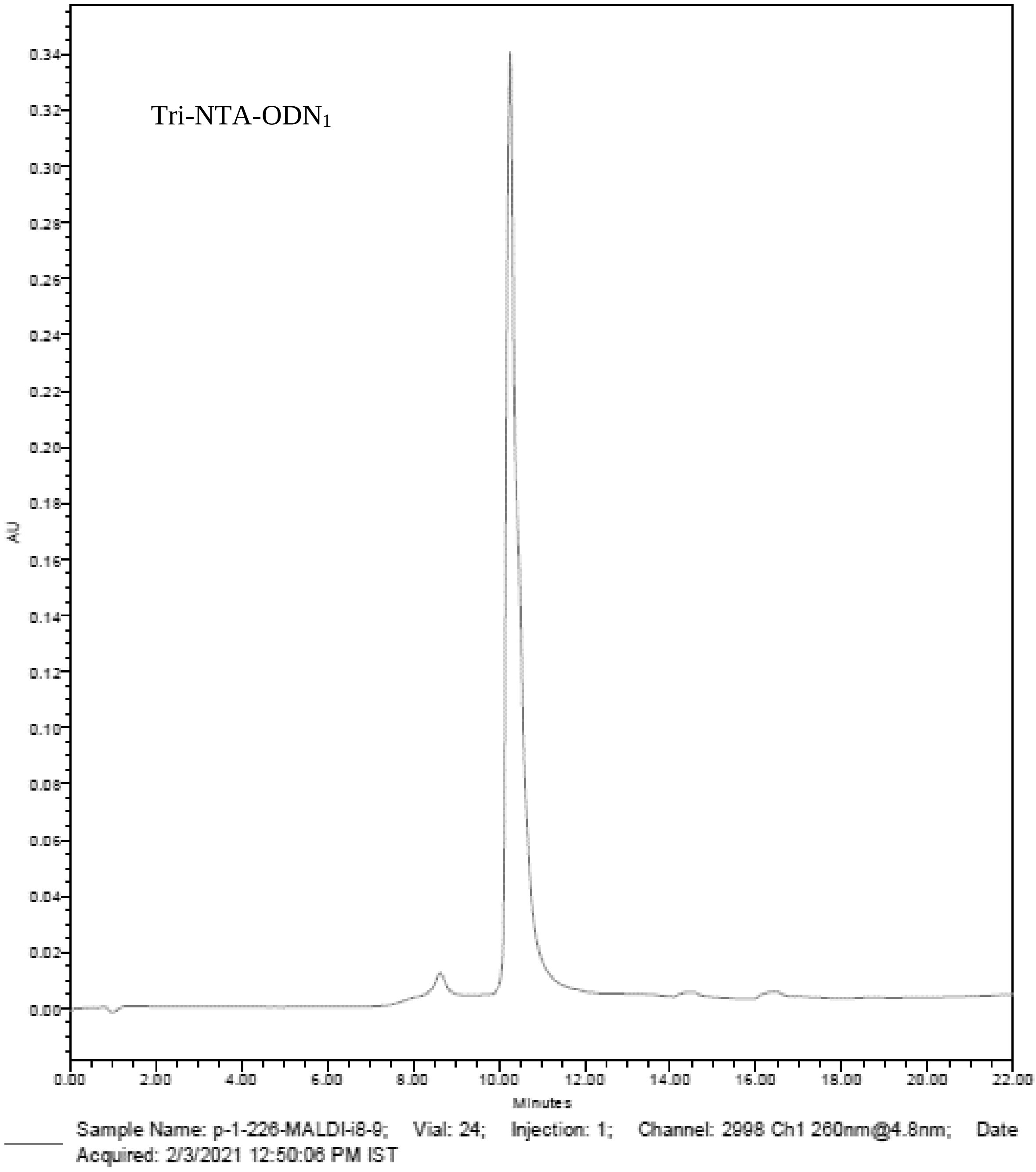

Tri-NTA-ODN1
AU
Minutes
0.34
0.32
0.30
0.28
0.26
0.24
0.22
0.20
0.18
0.16
0.14
0.12
0.10
0.08
0.06
0.04
0.02
0.00
0.00
2.00
4.00
6.00
8.00
10.00
12.00
14.00
16.00
18.00
20.00
22.00
Sample Name: p-1-226-MALDI-i8-9; Vial: 24; Injection: 1; Channel: 2998 Ch1 260nm@4.8nm; Date Acquired: 2/3/2021 12:50:06 PM IST

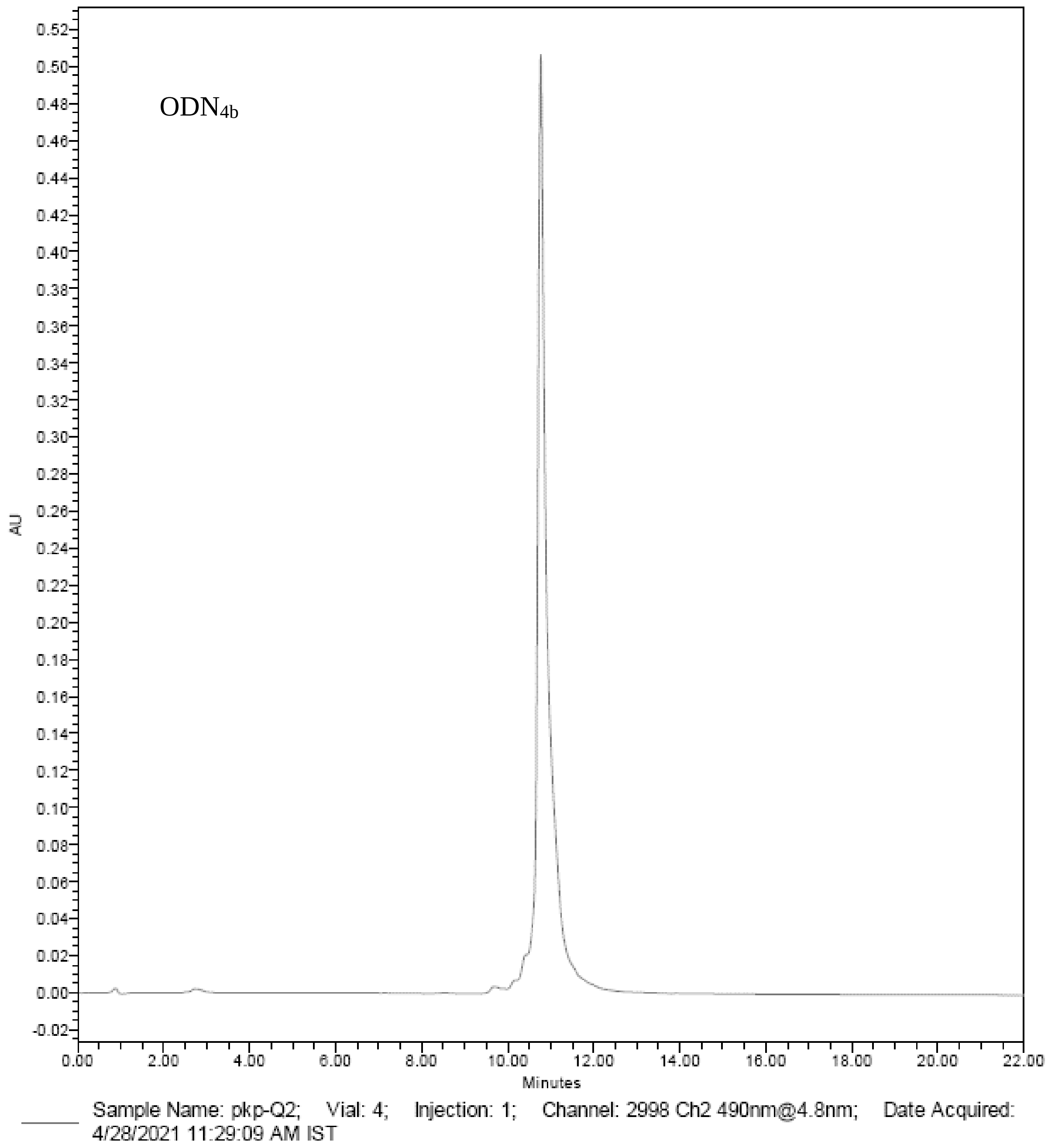
ODN4b
AU
Minutes
0.52
0.50
0.48
0.46
0.44
0.42
0.40
0.38
0.36
0.34
0.32
0.30
0.28
0.26
0.24
0.22
0.20
0.18
0.16
0.14
0.12
0.10
0.08
0.06
0.04
0.02
0.00
-0.02
0.00
2.00
4.00
6.00
8.00
10.00
12.00
14.00
16.00
18.00
20.00
22.00
Sample Name: pkp-Q2; Vial: 4; Injection: 1; Channel: 2998 Ch2 490nm@4.8nm; Date Acquired: 4/28/2021 11:29:09 AM IST

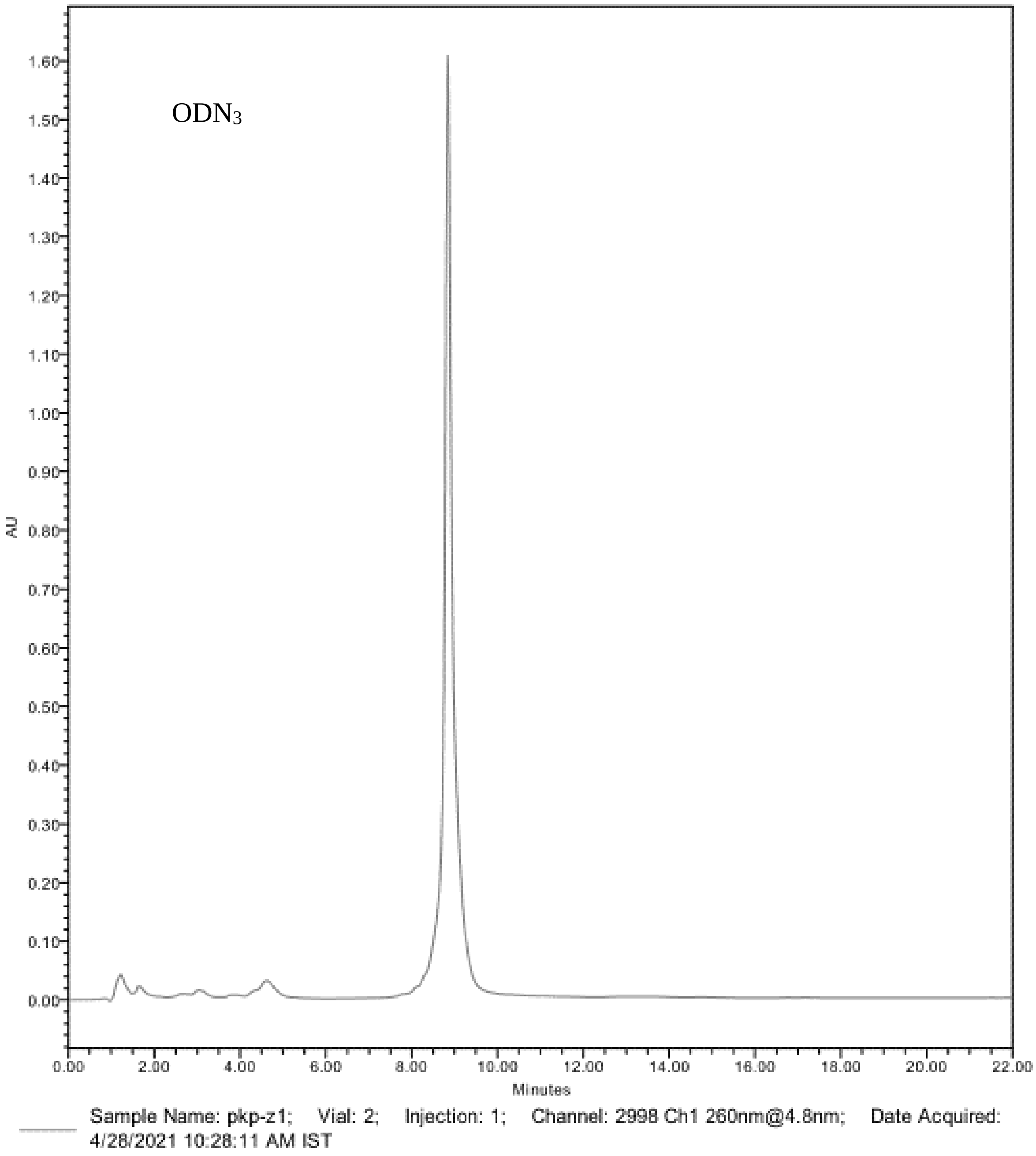
ODN3
1.60
1.50
1.40
1.30
1.20
1.10
1.00
0.90
0.80
0.70
0.60
0.50
0.40
0.30
0.20
0.10
0.00
AU
0.00
2.00
4.00
6.00
8.00
10.00
12.00
14.00
16.00
18.00
20.00
22.00
Minutes
Sample Name: pkp-z1; Vial: 2; Injection: 1; Channel: 2998 Ch1 260nm@4.8nm; Date Acquired: 4/28/2021 10:28:11 AM IST

MS-Chromatograms of ODNs used.

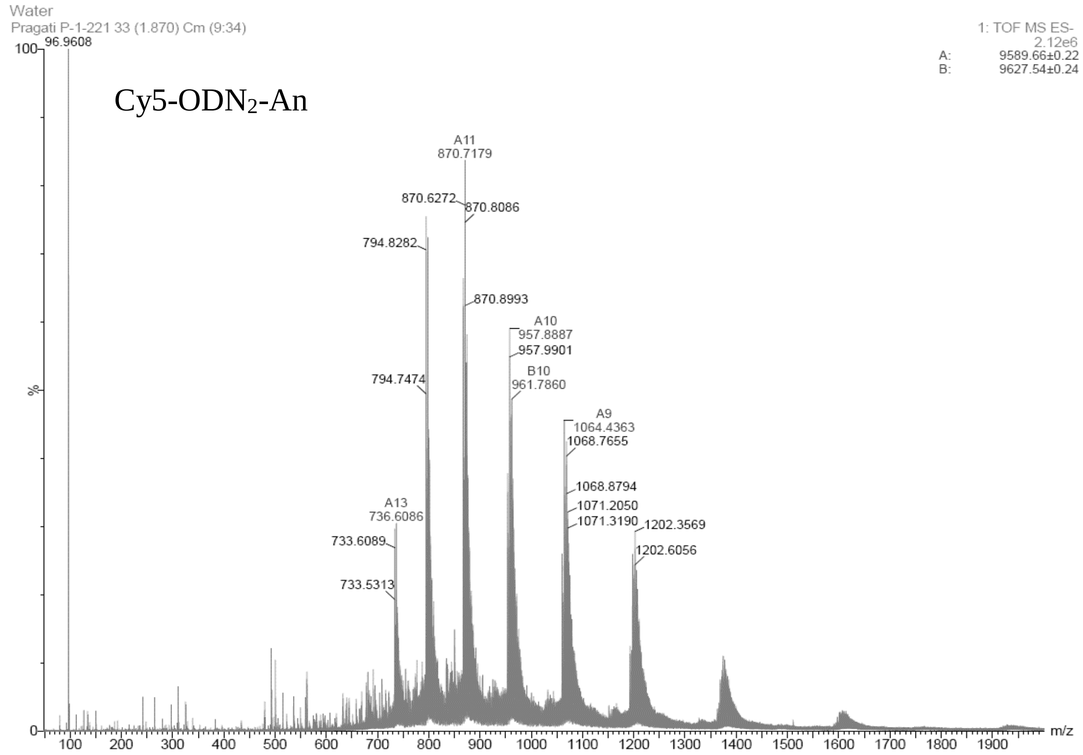

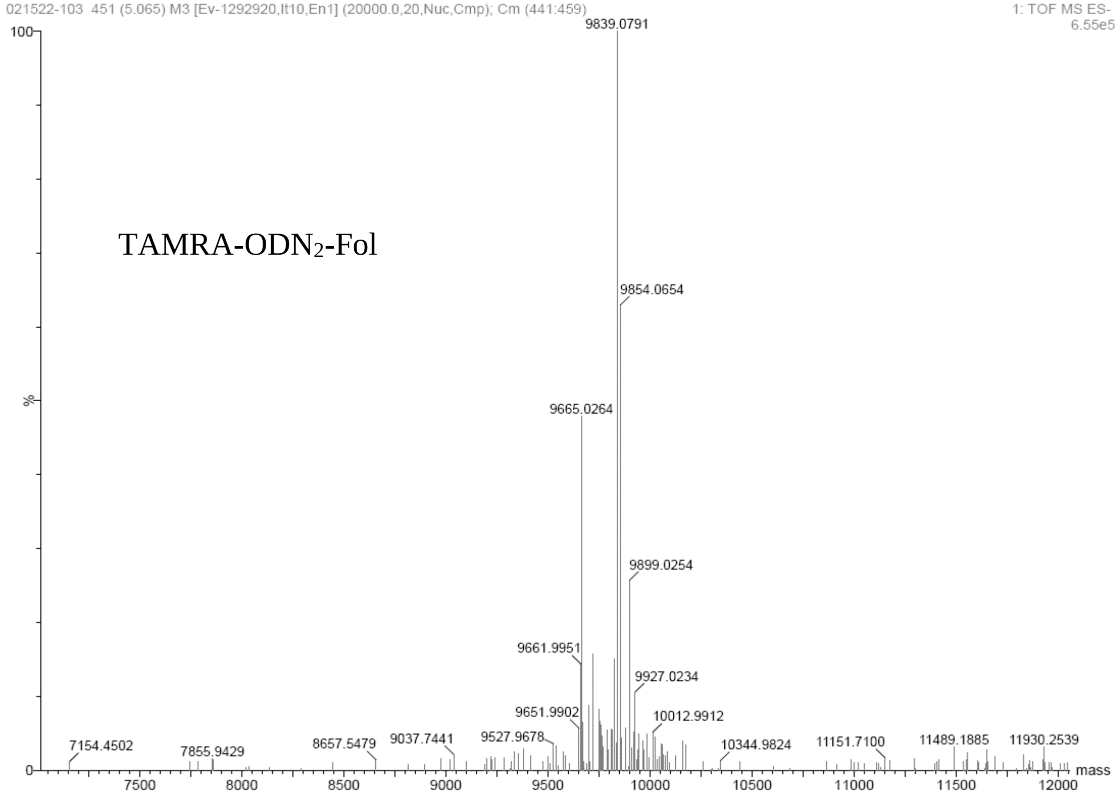

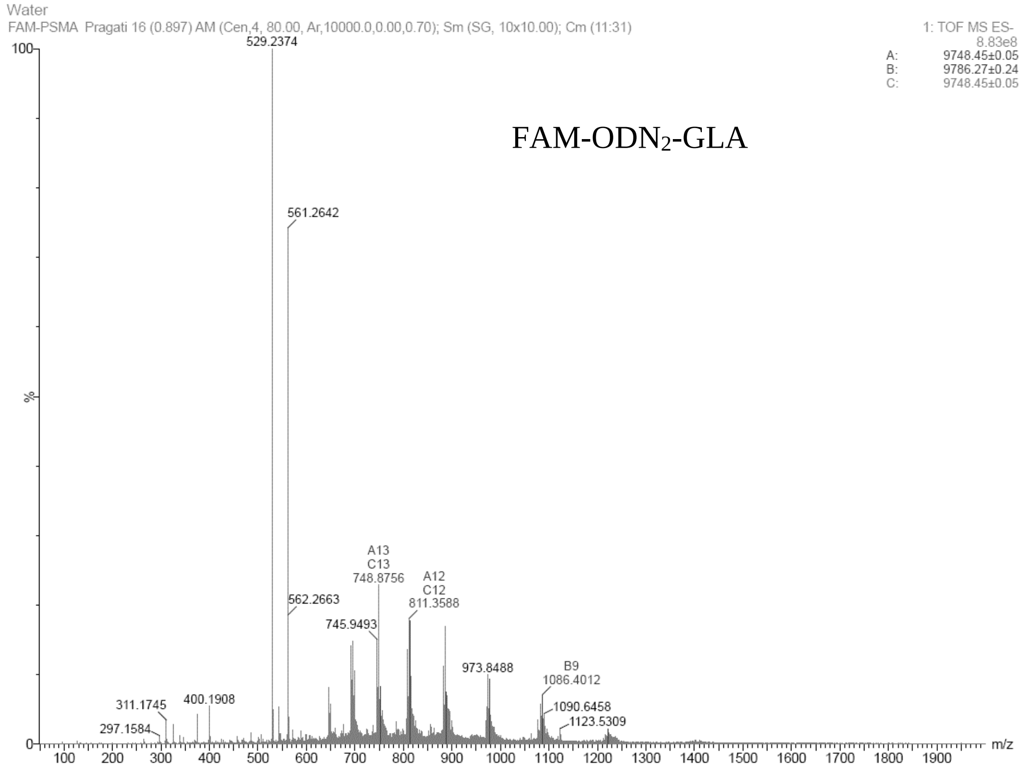
Water
FAM-PSMA Pragati 16 (0.897) AM (Cen,4, 80.00, Ar,10000.0,0.00,0.70); Sm (SG, 10x10.00); Cm (11:31)
1: TOF MS ES-
8.83e8
A: 9748.45±0.05
B: 9786.27±0.24
C: 9748.45±0.05
FAM-ODN2-GLA
529.2374
561.2642
562.2663
A13
C13
748.8756
745.9493
A12
C12
811.3588
973.8488
B9
1086.4012
1090.6458
1123.5309
400.1908
311.1745
297.1584
%
m/z

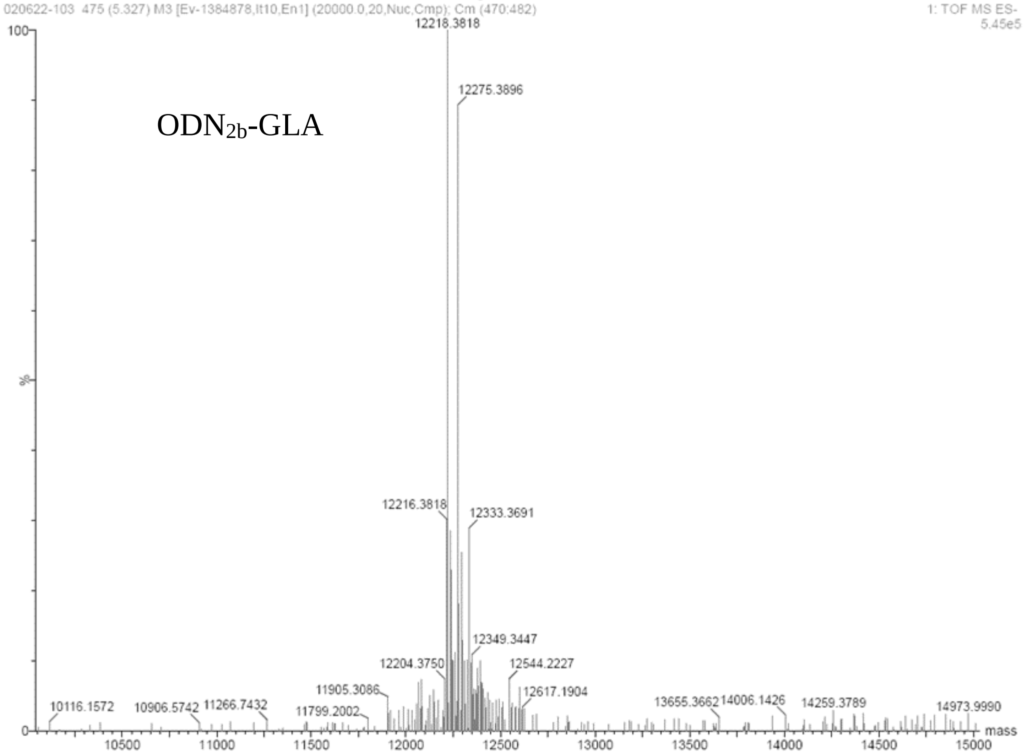
020622-103 475 (5.327) M3 [Ev-1384878,It10,En1] (20000.0,20,Nuc,Cmp); Cm (470:482)
1: TOF MS ES-
5.45e5
12218.3818
12275.3896
ODN2b-GLA
12216.3818
12333.3691
12349.3447
12204.3750
12544.2227
11905.3086
12617.1904
10116.1572
10906.5742
11266.7432
11799.2002
13655.3662
14006.1426
14259.3789
14973.9990
%
mass

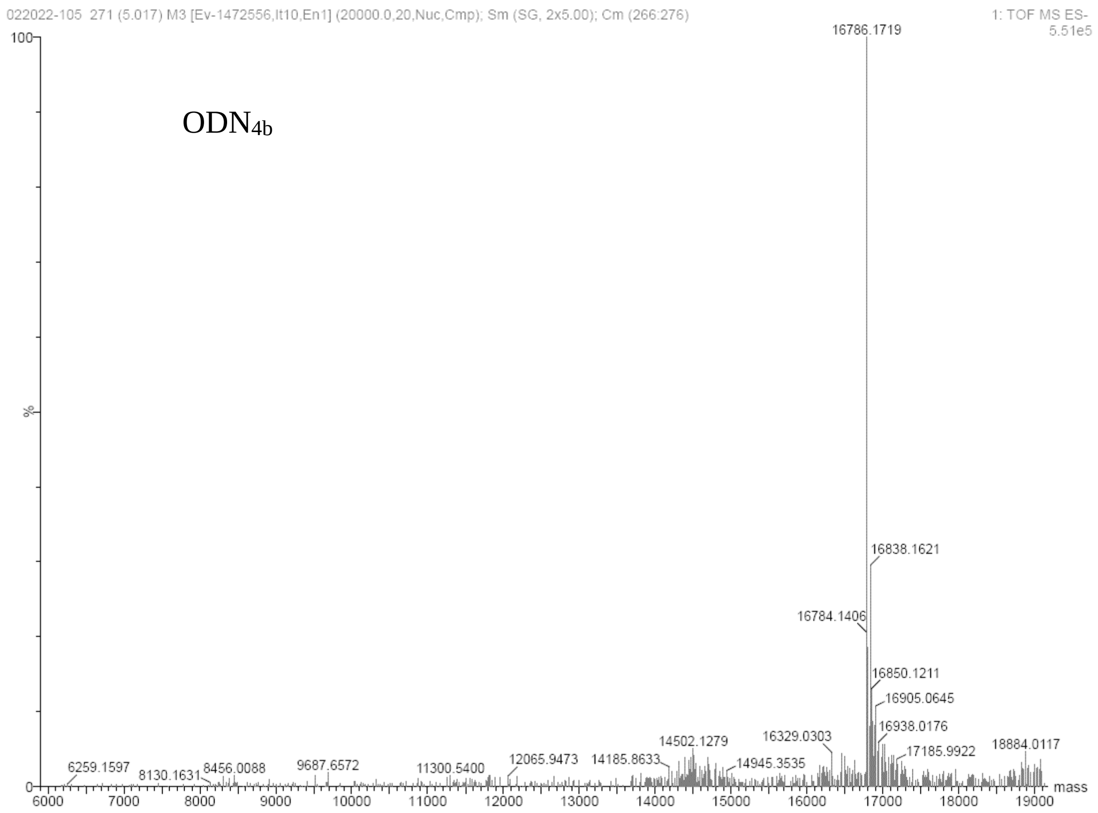
022022-105 271 (5.017) M3 [Ev-1472556,It10,En1] (20000.0,20,Nuc,Cmp); Sm (SG, 2x5.00); Cm (266:276)
1: TOF MS ES-
5.51e5
ODN4b
16786.1719
16838.1621
16784.1406
16850.1211
16905.0645
16938.0176
17185.9922
18884.0117
16329.0303
14502.1279
14945.3535
14185.8633
12065.9473
11300.5400
9687.6572
8456.0088
8130.1631
6259.1597
%
mass

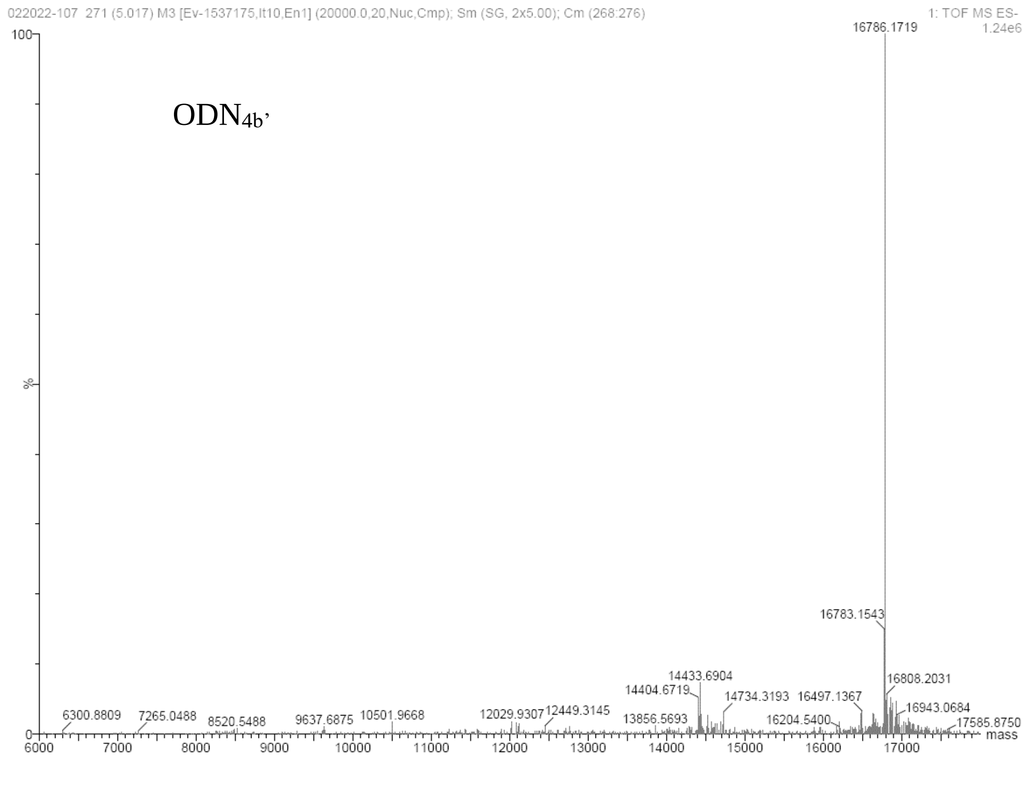
022022-107 271 (5.017) M3 [Ev-1537175,It10,En1] (20000.0,20,Nuc,Cmp); Sm (SG, 2x5.00); Cm (268:276)
1: TOF MS ES-
1.24e6
ODN4b'
16786.1719
16783.1543
16808.2031
16943.0684
17585.8750
16497.1367
16204.5400
14734.3193
14433.6904
14404.6719
13856.5693
12449.3145
12029.9307
10501.9668
9637.6875
8520.5488
7265.0488
6300.8809
%
mass

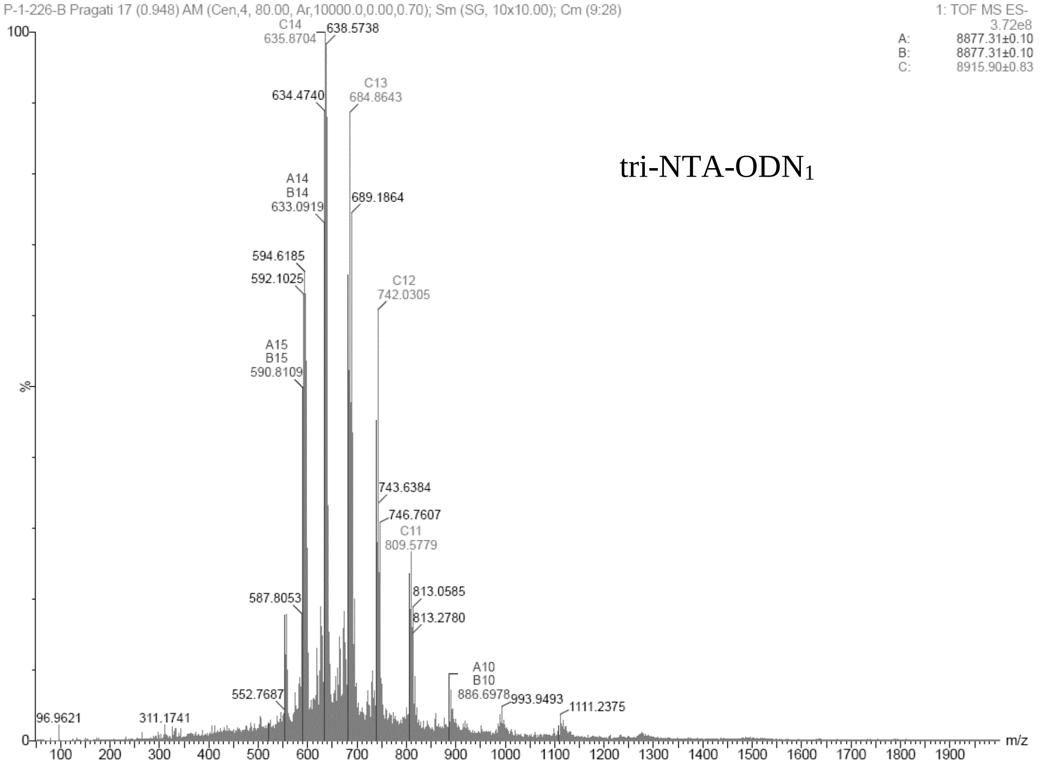
P-1-226-B Pragati 17 (0.948) AM (Cen,4, 80.00, Ar,10000.0,0.00,0.70); Sm (SG, 10x10.00); Cm (9:28)
1: TOF MS ES-
3.72e8
A: 8877.31±0.10
B: 8877.31±0.10
C: 8915.90±0.83
tri-NTA-ODN1
C14 635.8704
638.5738
634.4740
C13 684.8643
A14 B14 633.0919
689.1864
594.6185
592.1025
C12 742.0305
A15 B15 590.8109
743.6384
746.7607
C11 809.5779
813.0585
587.8053
813.2780
A10 B10 886.6978
552.7687
993.9493
1111.2375
96.9621
311.1741
%
m/z

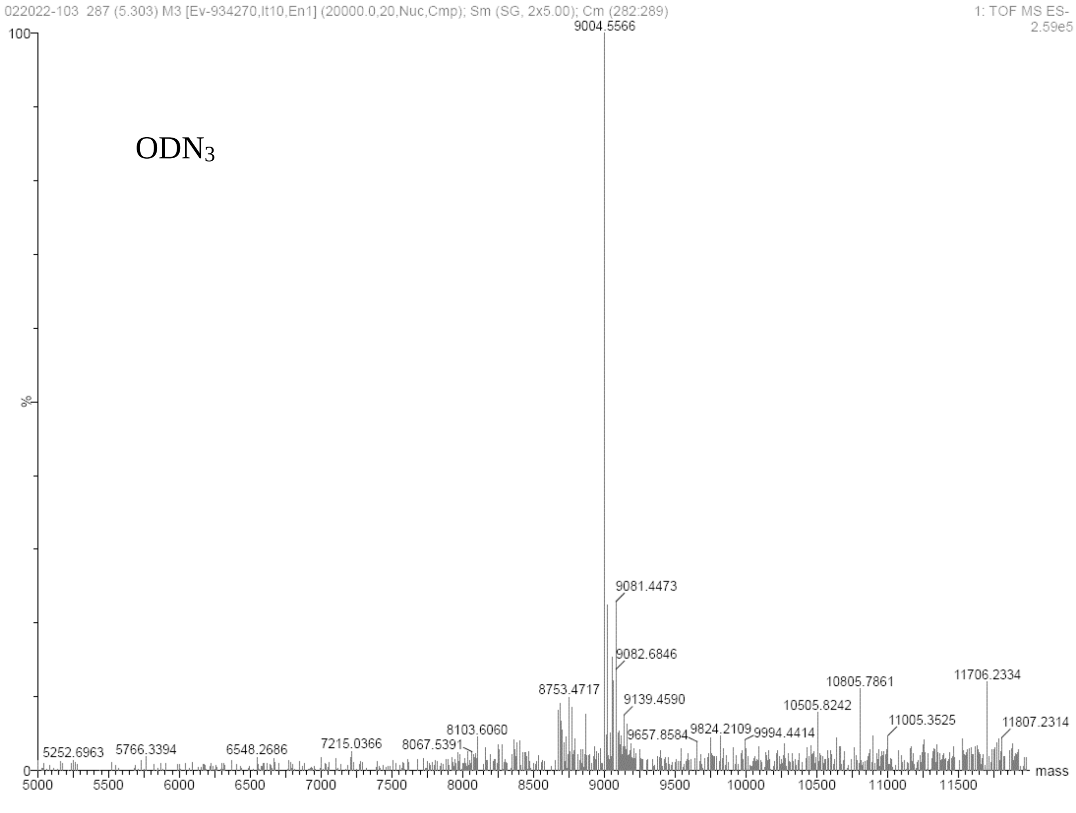
022022-103 287 (5.303) M3 [Ev-934270,It10,En1] (20000.0,20,Nuc,Cmp); Sm (SG, 2x5.00); Cm (282:289)
1: TOF MS ES-
2.59e5
9004.5566
ODN3
%
9081.4473
9082.6846
8753.4717
9139.4590
10805.7861
11706.2334
10505.8242
11005.3525
11807.2314
8103.6060
9657.8584
9824.2109
9994.4414
5252.6963
5766.3394
6548.2686
7215.0366
8067.5391
mass

^{1}H and ^{13}C NMR spectra of final compounds

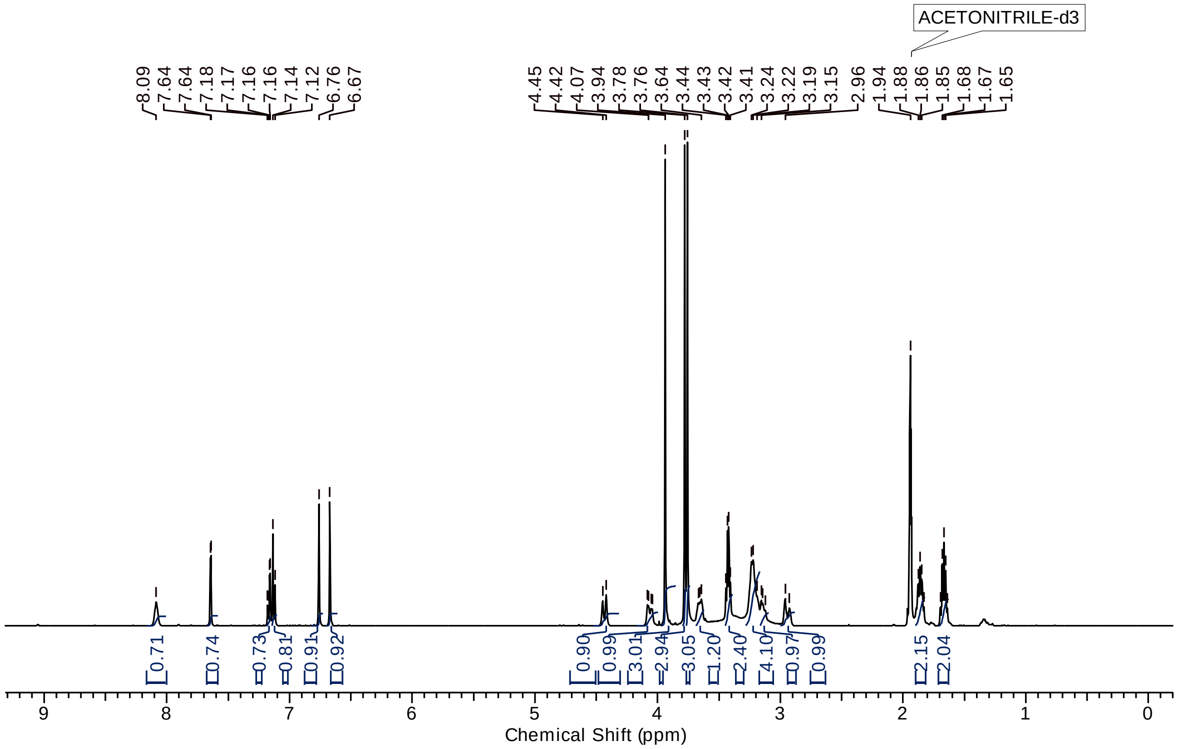

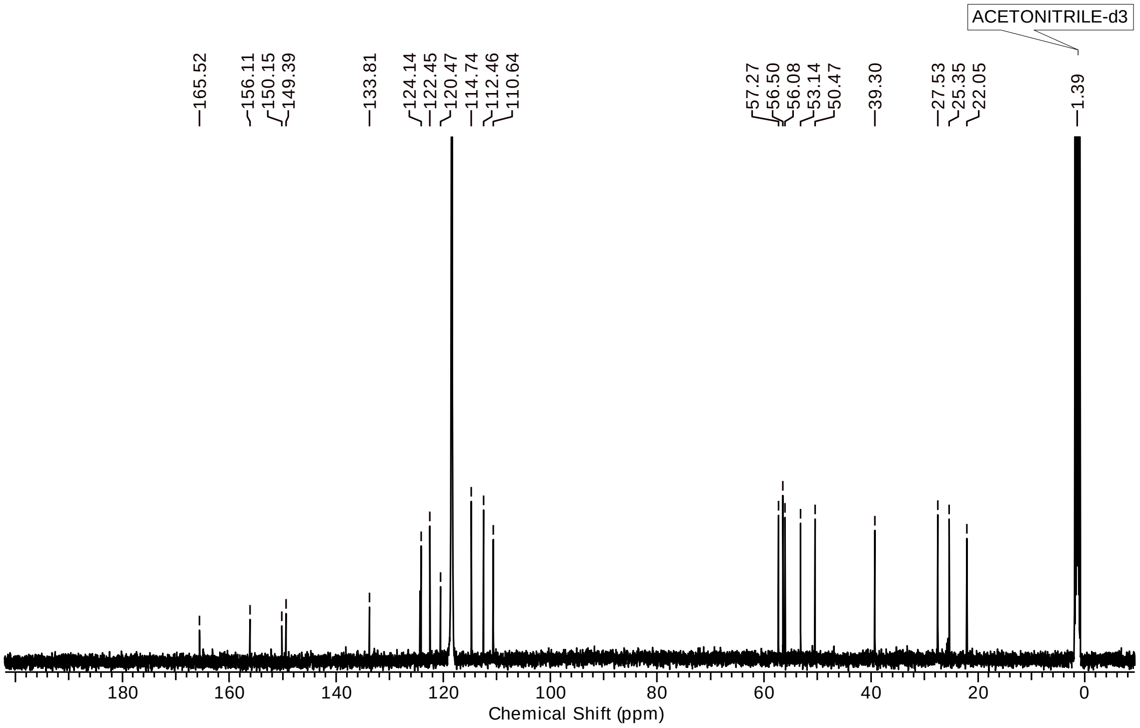

^{1}H and ^{13}C NMR spectra of **1**

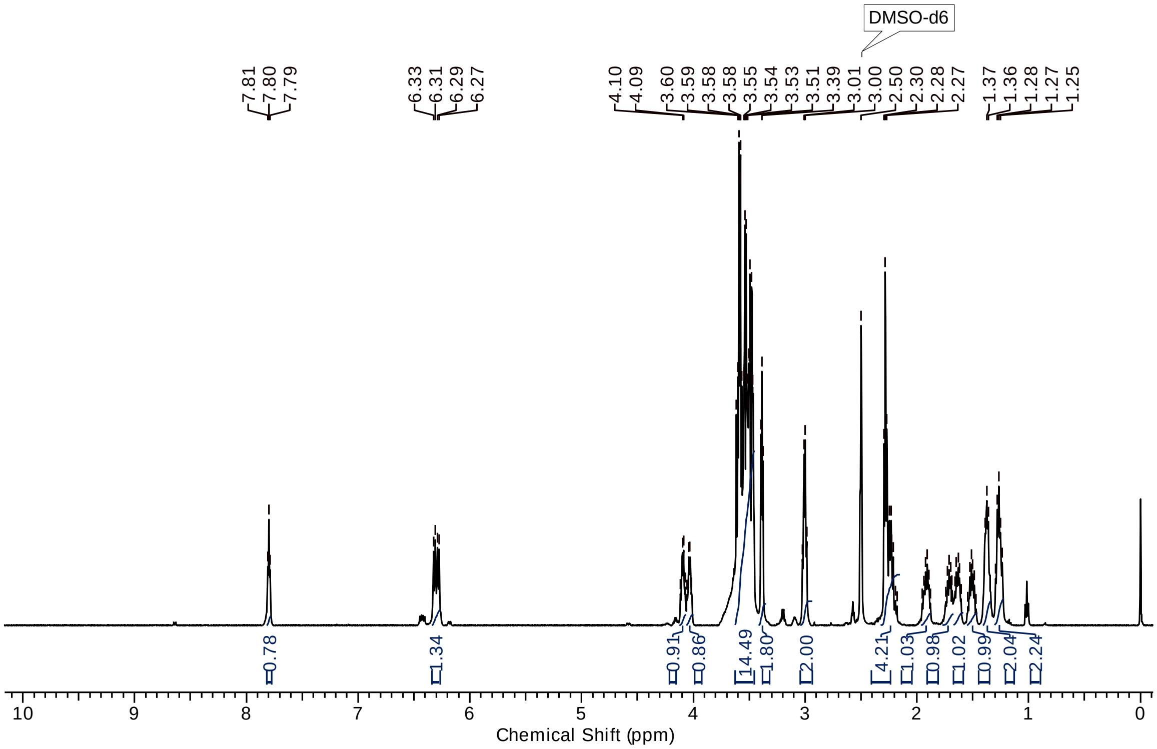

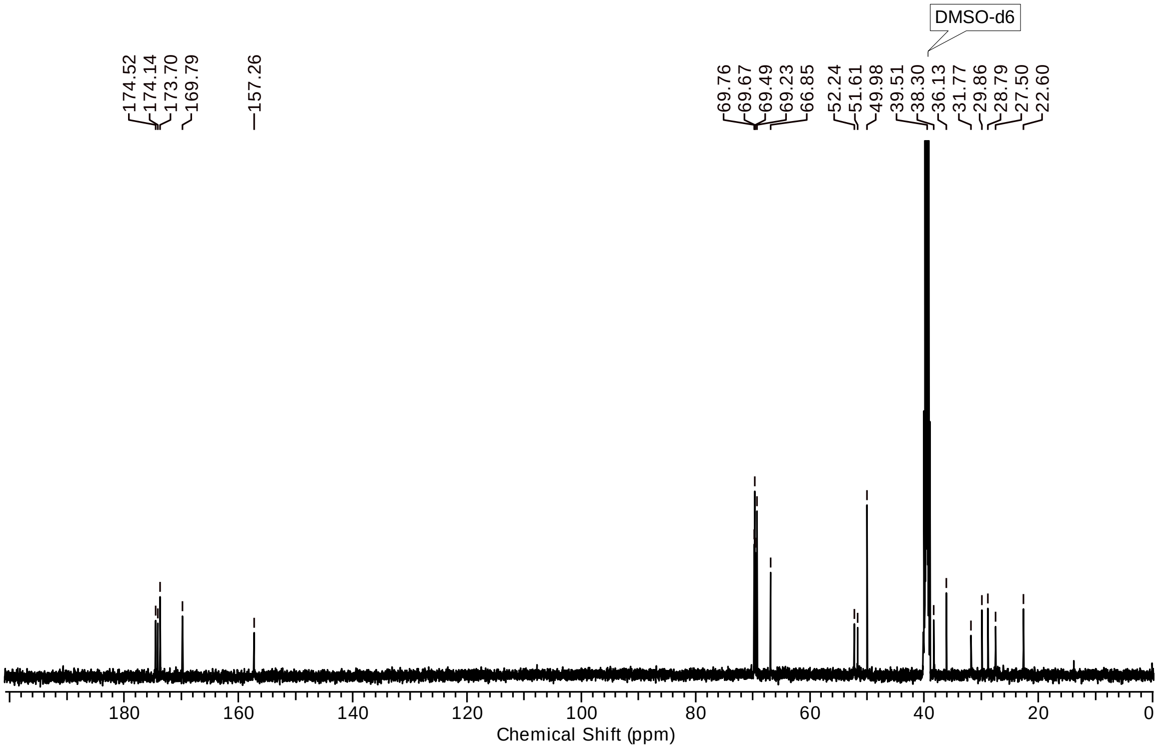

^{1}H and ^{13}C NMR spectra of **2**

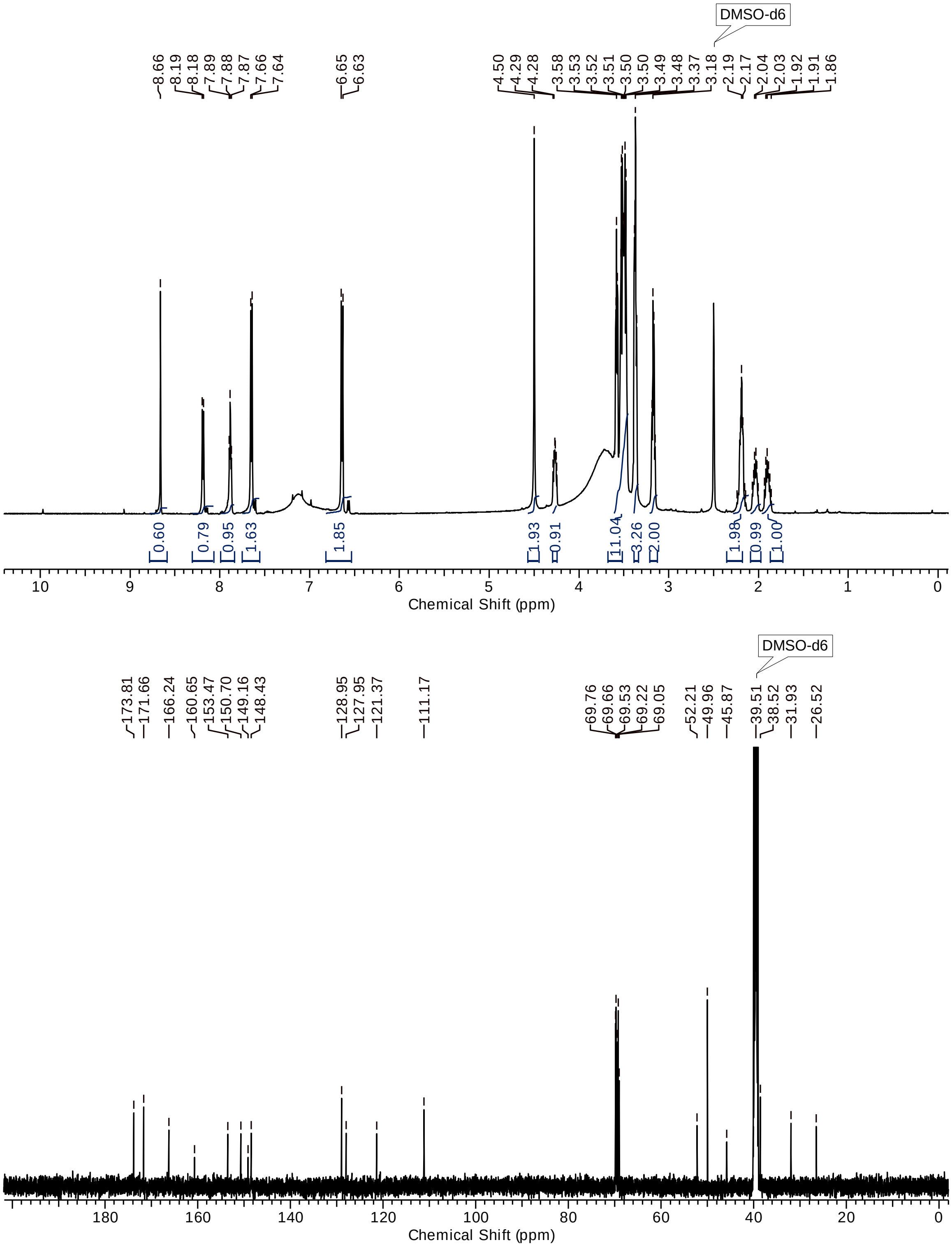

^{1}H and ^{13}C NMR spectra of **3**

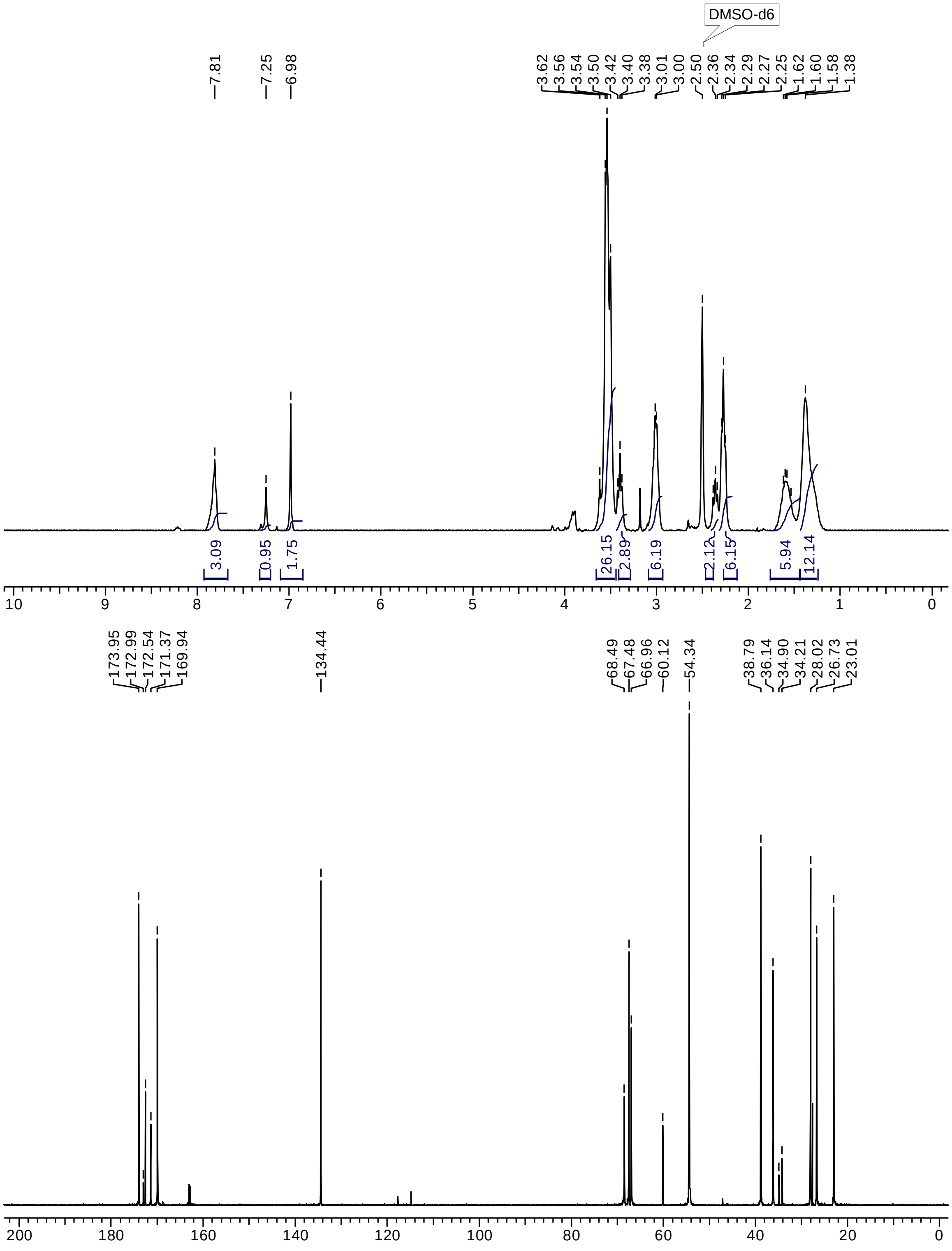

^{1}H and ^{13}C NMR spectra of **4**

References:

1. Adler, S.; Motiei, L.; Mankovski, N.; Cohen, H.; Margulies, D. Fluorescent labelling of cell surface proteins on a solid support. *Isr. J. Chem.* **2021**, *61*, 239-243.
2. Mankovski, N.; Prasad, P. K.; Oppenheimer, N.; Raviv, G.; Dadosh, T.; Unger, T.; Salame, T.; Motiei, L.; Margulies, D. Decorating bacteria with self-assembled synthetic receptors. *Nat. Commun.* **2020**, *11*, 1299-1310.
3. Paulos, C. M.; Reddy, J. A.; Leamon, C. P.; Turk, M. J.; Low P. S. Ligand binding and kinetics of folate receptor recycling in vivo: impact on receptor-mediated drug delivery. *Mol. Pharmacol.* **2004**, *66*, 1406-1414.
